# Organocatalytic asymmetric Henry reaction of 1*H*-pyrrole-2,3-diones with bifunctional amine-thiourea catalysts bearing multiple hydrogen-bond donors

**DOI:** 10.3762/bjoc.12.31

**Published:** 2016-02-16

**Authors:** Ming-Liang Zhang, Deng-Feng Yue, Zhen-Hua Wang, Yuan Luo, Xiao-Ying Xu, Xiao-Mei Zhang, Wei-Cheng Yuan

**Affiliations:** 1 National Engineering Research Center of Chiral Drugs, Chengdu Institute of Organic Chemistry, Chinese Academy of Sciences, Chengdu 610041, China; 2 University of Chinese Academy of Sciences, Beijing 100049, China

**Keywords:** asymmetric catalysis, bifunctional catalysts, Henry reaction, organocatalysis, 1*H*-pyrrole-2,3-diones

## Abstract

For the first time, a catalytic asymmetric Henry reaction of 1*H*-pyrrole-2,3-diones was achieved with a chiral bifunctional amine-thiourea as a catalyst possessing multiple hydrogen-bond donors. With this developed method, a range of 3-hydroxy-3-nitromethyl-1*H*-pyrrol-2(3*H*)-ones bearing quaternary stereocenters were obtained in acceptable yield (up to 75%) and enantioselectivity (up to 73% ee).

## Introduction

Asymmetric organocatalysis has been demonstrated to be an effective and versatile strategy in facilitating a variety of organic transformations over the past decade, and numerous catalytic asymmetric reactions have been developed with various activation modes [1–6]. In this realm, chiral bifunctional catalysts, possessing two active sites, have captured tremendous attention in particular due to their unique ability of the simultaneous activation of the nucleophile and the electrophile in the same transition state [7–11]. Among them, chiral bifunctional thioureas bearing multiple hydrogen-bond donors have been successfully used as chiral organocatalysts for the asymmetric Michael addition and Mannich reactions [12–14]. Meanwhile, the Henry reaction is one of the most important carbon–carbon bond-forming reactions that provides straightforward access to β-nitroalcohols, which can be further transformed into amino-alcohols, amino acids and carbonyl compounds [15]. Much attention has been devoted to the development of an efficient catalytic asymmetric version of this reaction from readily accessible nitroalkanes and carbonyl compounds [16], such as aldehydes [17–19], α-ketoesters [20], α-ketophosphonates [21–22], fluoromethyl ketones [23–24] and isatins [25–26]. Despite these significant advances described above, the use of more challenging ketones with heterocyclic structures as Henry acceptors has remained relatively less explored. In this context, developing a new Henry reaction for the construction of the useful and versatile β-nitroalcohol scaffolds is still desirable.

**Scheme 1:**
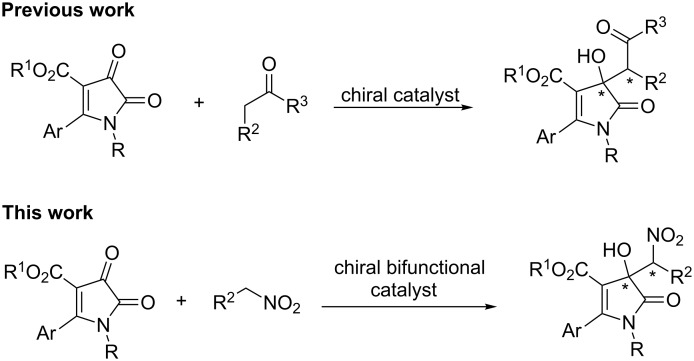
The strategy to construct chiral 3-substituted-3-hydroxy-1*H*-pyrrol-2(3*H*)-ones.

Pyrrole skeletons represent an important class of heterocycles and are frequently found in many biologically active molecules and natural products [27–28]. Particularly, 3-substituted-3-hydroxy-1*H*-pyrrol-2(3*H*)-one derivatives exhibit a wide spectrum of biological activities [29]. The reaction of 1*H*-pyrrole-2,3-diones with various nucleophiles should be a straightforward way to access diverse and interesting 3-substituted-3-hydroxy-1*H*-pyrrol-2(3*H*)-ones. A survey of the literature reveals that the study of 1*H*-pyrrole-2,3-diones is mainly focused on three-component spiro-heterocyclization reactions [30–32]. However, for the catalytic asymmetric transformation, only one example of an aldol reaction of 1*H*-pyrrole-2,3-diones with ketones has been reported so far (Scheme 1) [33]. Recently, our group developed a chiral bifunctional multiple hydrogen-bond amine-thiourea-catalyzed Michael reaction of acetyl phosphonates with nitroolefins, giving a series of β-substituted nitro compounds with excellent stereoselectivity [34]. Therefore, as part of our research program aimed at establishing new methods for the construction of quaternary stereocenters [35–37], we envisioned that the Henry reaction of nitroalkanes with 1*H*-pyrrole-2,3-diones should take place with a chiral bifunctional amine-thiourea catalyst, leading to 3-hydroxy-3-nitromethyl-1*H*-pyrrol-2(3*H*)-ones bearing quaternary stereocenters (Scheme 1) [14]. Notably, this work represents the first example of 1*H*-pyrrole-2,3-diones used as Henry acceptors for the asymmetric reaction. Herein, we report our preliminary results on this subject.

## Results and Discussion

We started our studies with the reaction of ethyl 1-benzyl-4,5-dioxo-2-phenyl-4,5-dihydro-1*H*-pyrrole-3-carboxylate (**1a**) and nitromethane (**2a**) in the presence of various chiral bifunctional organocatalysts **3a**–**e** in dichloromethane (Table 1). As expected, the reaction proceeded and gave the desired product **4a** in 18% yield and 28% ee with cinchonidine and L-valine-based catalyst **3a** (Table 1, entry 1). The bifunctional, thiourea-tertiary amine catalyst **3b**, derived from quinine and L-valine, furnished a similar result to catalyst **3a** (Table 1, entry 2). Next, the reaction was attempted with catalyst **3c** derived from L-phenylalanine and catalyst **3d** derived from L-phenylglycine, and improvements in enantioselectivity were observed (Table 1, entries 3 and 4). Changing L-phenylalanine to D-phenylalanine furnished catalyst **3e**, and the enantioselectivity was improved to 61% ee (Table 1, entry 5). Having identified **3e** as the best catalyst, we undertook a solvent screening for this transformation with 20 mol % **3e** at 30 °C (Table 1, entries 6–12). Arenes such as toluene and mesitylene gave relatively lower ee values (Table 1, entries 6 and 7). In contrast, the reactions in ethers gave improved yield with slightly increased enantioselectivity (Table 1, entries 8–10). However, strong polar solvents such as acetonitrile and ethyl acetate proved inferior to this Henry reaction (Table 1, entries 11 and 12). The solvent survey revealed that THF was the suitable solvent in terms of the yield and enantioselectivity (Table 1, entry 8).

**Table 1:** Screening of the catalysts and solvents.^a^

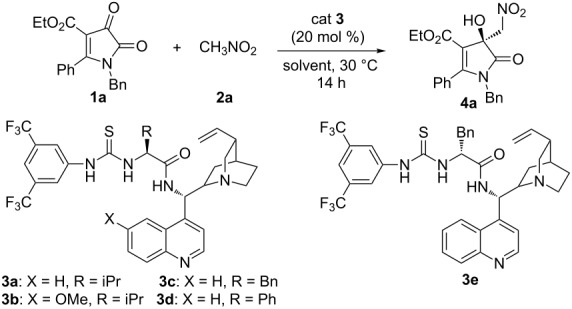				

Entry	Catalyst	Solvent	Yield (%)^b^	ee (%)^c^

1	**3a**	DCM	18	28
2	**3b**	DCM	25	27
3	**3c**	DCM	25	38
4	**3d**	DCM	29	41
5	**3e**	DCM	23	61
6	**3e**	toluene	24	46
7	**3e**	mesitylene	36	58
8	**3e**	THF	61	61
9	**3e**	dioxane	59	53
10	**3e**	Et_2_O	48	62
11	**3e**	CH_3_CN	14	40
12	**3e**	EtOAc	18	59

^a^Unless otherwise noted, the reactions were carried out with **1a** (0.2 mmol), **2a** (2.0 mmol), catalyst **3** (20 mol %) in solvent (2 mL) at 30 °C for 14 h. ^b^Isolated yield. ^c^Determined by chiral HPLC analysis.

To further optimize the reaction conditions, the substituent at the nitrogen atom in 1*H*-pyrrole-2,3-diones **1** and the substrate concentration were investigated. The results are summarized in Table 2. It was found that an isopropyl group furnished a better yield and ee value than a benzyl group (Table 2, entry 2 vs entry 1). Changing the isopropyl group to a methyl group decreased the enantioselectivity (Table 2, entry 3). To enhance the reactivity of the substrate, we also tried to introduce some electron-withdrawing substituents on the nitrogen atom. However, we could not obtain the desired substrates. Afterwards, upon the investigation of substrate concentration (Table 2, entries 4 and 5), it was found that increasing the substrate concentration could slightly increase the yield with unchanged enantioselectivity (Table 2, entry 4). Ultimately, the effects of the reaction temperature and the additive were also examined and no improved results were obtained (Table 2, entries 6–8). Based on these observations, the most appropriate conditions for the Henry reaction could be established: 1 equiv of **1a** and 10 equiv of **2a** with 20 mol % catalyst **3e** in 1.0 mL THF at 30 °C (Table 2, entry 4).

**Table 2:** Further optimization of conditions.^a^

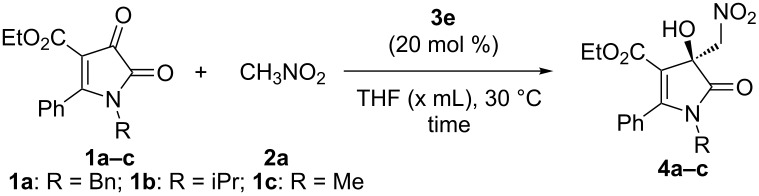						

Entry	**1**	x	Time (h)	**4**	Yield (%)^b^	ee (%)^c^

1	**1a**	2	14	**4a**	61	61
2	**1b**	2	14	**4b**	64	71
3	**1c**	2	14	**4c**	19	57
4	**1b**	1	14	**4b**	69	71
5	**1b**	3	14	**4b**	60	70
6^d^	**1b**	1	14	**4b**	64	46
7^e^	**1b**	1	62	**4b**	trace	ND
8^f^	**1b**	1	14	**4b**	75	66

^a^Unless otherwise noted, the reactions were carried out with **1a** (0.2 mmol), **2a** (2.0 mmol) and catalyst **3e** (20 mol %) in THF for the specified reaction time. ^b^Isolated yield. ^c^Determined by chiral HPLC analysis. ^d^Run at 50 °C; ^e^Run at 0 °C. ^f^4 Å MS was added. ND, not determined.

**Figure 1:**
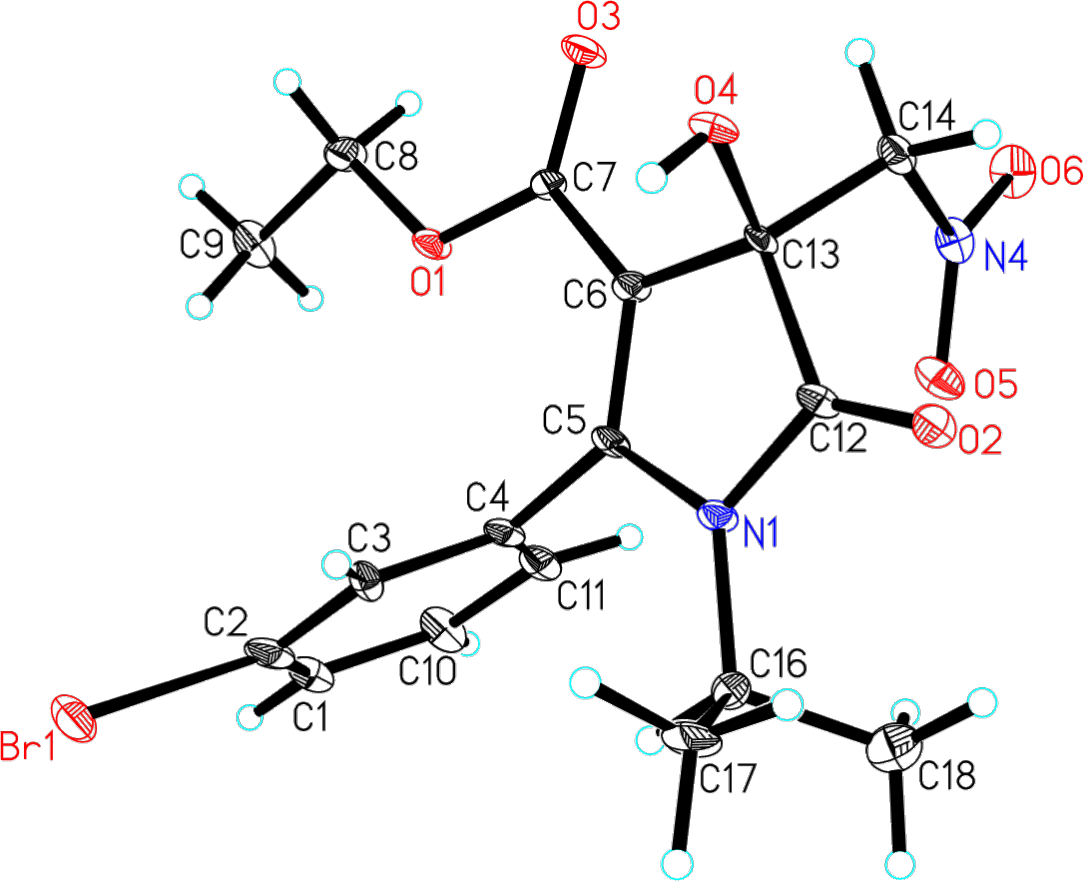
The X-ray structure of compound **4i**.

Under the optimal reaction conditions, the substrate scope and the limitation of this organocatalytic Henry reaction were explored. The results are summarized in Table 3. First, we investigated the effects of the alkyl ester of 1*H*-pyrrole-2,3-diones **1**. The replacement of the ethyl group by either a methyl or a butyl group had only a slight effect on the ee value but an obvious effect on the yield (Table 3, entries 1 and 2 vs Table 2, entry 4). After introducing different substituent groups on the phenyl ring of substrate **1**, it was observed that the reactions take place, and the corresponding products in 44–73% yield with up to 73% ee were furnished. This was true regardless of the electronic nature and position of the substituents on the phenyl ring (Table 3, entries 3–10). When the substituent was in the ortho-position of the phenyl ring of the R^2^ group, a diastereoselectivity was observed that may be due to the steric hindrance of ortho substituents led to the atropisomers (Table 3, entry 3). A similar result was observed for the bulky 1-naphthyl group (Table 3, entry 11). The reaction was also performed with a 2-thienyl residue and the desired product was obtained in 51% yield and 71% ee (Table 3, entry 10). In addition, substrate **1o** with no substituent on the nitrogen atom gave the desired product in 41% and 48% ee (Table 3, entry 12). The introduction of a phenyl group on the nitrogen atom had a negative effect on the reaction (Table 3, entry 13). Nitroethane was also used to react with 1*H*-pyrrole-2,3-dione **1b**, and a good diastereoselectivity was achieved with an unfavorable yield and ee value (Table 3, entry 14). For the substrates with methoxy groups on the phenyl rings, the reaction gave relatively better results (Table 3, entries 4 and 7). We also tested the substrates **1q** and **1r** containing a methyl ester, and acceptable results were obtained (Table 3, entries 15 and 16). The absolute configuration of the major isomer **4i** was unambiguously determined to be *S* by single-crystal X-ray analysis (Figure 1) [38]. The configurations of the other products were assigned by analogy.

**Table 3:** Scope of the reaction.^a^

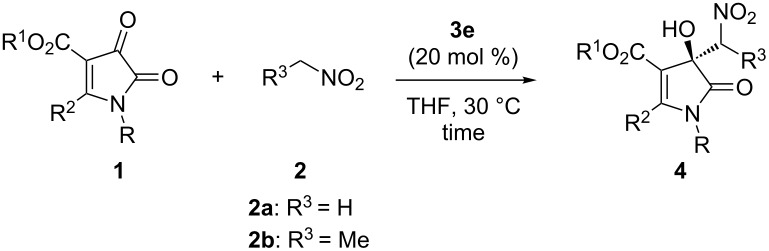						

Entry	R/ R^1^/R^2^	**2**	Time (h)	**4**	Yield (%)^b^	ee (%)^c^

1	iPr/Me/Ph (**1d**)	**2a**	14	**4d**	55	65
2	iPr/*n*-Bu/Ph (**1e**)	**2a**	70	**4e**	52	70
3	iPr/Et/2-ClC_6_H_4_ (**1f**)	**2a**	62	**4f**	53	73^d^
4	iPr/Et/3-MeOC_6_H_4_ (**1g**)	**2a**	62	**4g**	73	71
5	iPr/Et/3-ClC_6_H_4_ (**1h**)	**2a**	120	**4h**	47	71
6	iPr/Et/3-BrC_6_H_4_ (**1i**)	**2a**	120	**4i**	44	70
7	iPr/Et/4-MeOC_6_H_4_ (**1j**)	**2a**	62	**4j**	61	72
8	iPr/Et/4-MeC_6_H_4_ (**1k**)	**2a**	48	**4k**	64	69
9	iPr/Et/4-FC_6_H_4_ (**1l**)	**2a**	62	**4l**	73	70
10	iPr/Et/2-thienyl (**1m**)	**2a**	120	**4m**	51	71
11	iPr/Et/1-naphthyl (**1n**)	**2a**	120	**4n**	75	51^e^
12	H/Me/Ph (**1o**)	**2a**	120	**4o**	41	48
13	Ph/Et/Ph (**1p**)	**2a**	120	**4p**	42	29
14	iPr/Et/Ph (**1b**)	**2b**	24	**4q**	23	22^f^
15	iPr/Me/4-MeOC_6_H_4_ (**1q**)	**2a**	84	**4r**	51	69
16	iPr/Me/3-MeOC_6_H_4_ (**1r**)	**2a**	120	**4s**	71	58

^a^Unless otherwise noted, the reactions were carried out with **1** (0.2 mmol), **2** (2.0 mmol). ^b^Isolated yield. ^c^Determined by chiral HPLC analysis. ^d^ee for the major isomer and ee of another isomer is 60%, and 54:46 dr was observed. ^e^ee for the major isomer and ee of another isomer is 49%, and 53:47 dr was observed. ^f^ee for the major isomer, and 94:6 dr was observed.

**Figure 2:**
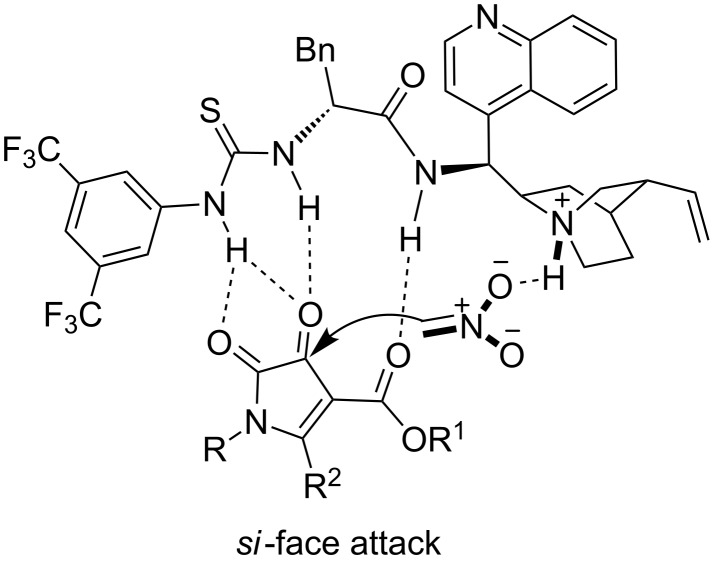
A proposed transition state for the asymmetric Henry reaction.

On the basis of our experimental results and the related reports about the bifunctional activation mode of nitromethane with different electrophiles [14,17,39], we propose a possible model to explain the stereochemistry of this transformation. As shown in Figure 2, nitromethane is activated by the tertiary amine to form the nitro enolate. Simultaneously, the 1*H*-pyrrole-2,3-diones **1** are orientated by the multiple hydrogen bonds of the catalyst. Thus, the nitro enolate attacks the keto carbonyl group of 1*H*-pyrrole-2,3-diones (to the *si-*face) to furnish the corresponding product with *S*-configuration (Figure 2).

## Conclusion

In conclusion, we have developed an asymmetric Henry reaction of 1*H*-pyrrole-2,3-diones with a chiral bifunctional amine-thiourea possessing multiple hydrogen-bond donors as the catalyst. With the developed protocol, a range of 3-hydroxy-3-nitromethyl-1*H*-pyrrol-2(3*H*)-ones bearing quaternary stereocenters were obtained in good yield (up to 75%) and with moderate to good enantioselectivity (up to 73% ee). A possible transition-state model, characterized by the bifunctional catalyst acting as a multiple hydrogen-bond donor, is also proposed. The application of 1*H*-pyrrole-2,3-diones in the catalytic asymmetric reactions for the preparation of biologically relevant compounds is currently underway.

## Supporting Information

File 1 General procedure, analytical data and spectra of all compounds, methods for conversion.

File 2 Single-crystal X-ray analysis of **4i**.
