# Supplementary material for: Organocatalytic asymmetric Henry reaction of 1H-pyrrole-2,3-diones with bifunctional amine-thiourea catalysts bearing multiple hydrogen-bond donors
Source: Beilstein J Org Chem. 2016 Feb 16;12:295–300. doi: 10.3762/bjoc.12.31 (PMC4778496; doi:10.3762/bjoc.12.31)
Supplement: File 1 — General procedure, analytical data and spectra of all compounds, methods for conversion. [file Beilstein_J_Org_Chem-12-295-s001.pdf]

Supporting Information

for

Organocatalytic asymmetric Henry reaction of 1*H*-pyrrole-2,3-diones with bifunctional amine-thiourea catalysts bearing multiple hydrogen-bond donors

Ming-Liang Zhang[1,2], Deng-Feng Yue[1,2], Zhen-Hua Wang[1,2], Yuan Luo[1,2], Xiao-Ying Xu*,[1], Xiao-Mei Zhang[1] and Wei-Cheng Yuan*,[1]

Address: [1]National Engineering Research Center of Chiral Drugs, Chengdu Institute of Organic Chemistry, Chinese Academy of Sciences, Chengdu 610041, China and [2]University of Chinese Academy of Sciences, Beijing 100049, China

Email: Xiao-Ying Xu*-xuxy@cioc.ac.cn; Wei-Cheng Yuan* - yuanwc@cioc.ac.cn

*Corresponding author

General procedure, analytical data and spectra of all compounds, methods for conversion

Table of Contents

General Methods	S2
General Procedure for Synthesis of compounds **4**	S2
Analytical data for compounds **4**	S2–S9
Crystal data for **4i**	S9–S10
References	S10
NMR and HPLC Spectra for compounds **4**	S11–S49

General methods

Reagents were purchased from commercial sources and were used as received unless otherwise mentioned. Reactions were monitored by TLC. ^{1}H NMR spectra were recorded on Bruker 300 (300 MHz) spectrophotometers. Chemical shifts (δ) are reported from the solvent resonance as the internal standard ($CDCl_3$: δ 7.26, DMSO-d_6: δ 2.50). Data are given as follow: s (singlet), d (doublet), t (triplet), q (quadruple) or m (multiples), coupling constants (Hz) and integration. ^{13}C NMR spectra were recorded in ppm from tetramethylsilane (TMS) with the solvent resonance as the internal standard ($CDCl_3$ at 77.20 ppm, DMSO-d_6 at 39.51 ppm). Mass spectra were recorded on Bruker micrOTOF–Q II mass spectrometer. HPLC data was acquired using a Shimadzu LC-20A with Daicel AD-H, Daicel OD- H, Daicel IC column.

1*H*-Pyrrole-2,3-diones (**1a**–**s**) were prepared according to the reported procedures [1,2]. All reactions have been carried out with distilled and degassed solvents in oven-dried glassware. All the solvents were treated according to general methods. Column chromatography was performed using silica gel H.

General procedure for synthesis of compounds 4

A solution of compounds **1** (0.2 mmol), compounds **2** (2 mmol) and catalyst **3e** (20 mol %) in THF was stirred at 30 °C for the indicated time. Then the reaction mixture was directly charged onto a silica gel column and purified through flash chromatography to furnish the corresponding products **4**.

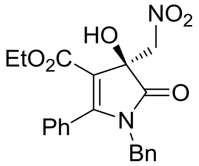

(*S*)-Ethyl 1-benzyl-4-hydroxy-4-(nitromethyl)-5-oxo-2-phenyl-4,5-dihydro-1*H*-pyrrole-3-carboxylate (**4a**). Light yellow oil; 48.3 mg, 61% yield; 61% ee; **^{1}H NMR (300 MHz, $CDCl_3$)** δ 7.43-7.40 (m, 1H), 7.33 (t, J = 7.6 Hz, 2H), 7.22-7.13 (m, 3H), 7.08 (d, J = 7.2 Hz, 2H), 6.90-6.85 (m, 2H), 5.26 (d, J = 13.0 Hz, 1H), 5.09 (d, J = 13.0 Hz, 1H), 4.56 (s, 2H), 4.18 (s, 1H), 4.01-3.92 (m, 2H), 0.91 (t, J = 7.1 Hz, 3H); **^{13}C NMR (75 MHz, $CDCl_3$)** δ 175.5, 162.7, 159.8, 135.5, 130.0, 128.5, 128.4, 128.3, 127.9, 127.6, 127.4, 107.4, 77.2, 74.0, 60.3, 44.6, 13.5; HPLC (Chiralpak OD-H column, EtOH/*n*-hexane = 10/90, flow rate 1.0 mL/min, λ = 254 nm, t_{major} = 9.21 min, t_{minor} = 8.06 min); $[\alpha]_D^{20}$ = +21.5 (*c* 2.195, $CHCl_3$); HRMS (ESI-TOF) calcd for $C_{21}H_{20}N_2NaO_6$ $[M + Na]^+$ 419.1214; found: 419.1206.

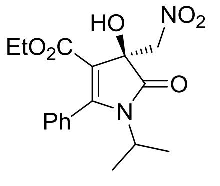

(*S*)-Ethyl 4-hydroxy-1-isopropyl-4-(nitromethyl)-5-oxo-2-phenyl-4,5-dihydro-1*H*-pyrrole-3-carboxylate (**4b**). Light yellow oil; 48.0 mg, 69% yield; 71% ee; **^{1}H NMR (300 MHz, $CDCl_3$)** δ 7.49-7.45 (m, 3H), 7.30 (d, *J* = 5.5 Hz, 1H), 7.23 (d, *J* = 6.1 Hz, 1H), 5.19 (d, *J* = 12.8 Hz, 1H), 4.99 (d, *J* = 12.8 Hz, 1H), 4.00 – 3.89 (m, 3H), 3.63-3.54 (m,1H), 1.37 (d, *J* = 6.9 Hz, 3H), 1.33 (d, *J* = 6.9 Hz, 3H), 0.90 (t, *J* = 7.1 Hz, 3H); **^{13}C NMR (75 MHz, $CDCl_3$)** δ 175.5, 162.7, 161.0, 130.0, 129.4, 128.5, 128.3, 128.3, 127.5, 106.6, 77.5, 73.7, 60.2, 47.3, 19.9, 19.0, 13.5; HPLC (Chiralpak OD-H column, *i*-PrOH/*n*-hexane = 10/90, flow rate 1.0 mL/min, λ = 254 nm, t_{major} = 7.56 min, t_{minor} = 6.27 min); $[\alpha]_D^{20}$ = +18.0 (*c* 2.200, $CHCl_3$); HRMS (ESI-TOF) calcd for $C_{17}H_{20}N_2NaO_6$ $[M + Na]^+$ 371.1214; found: 371.1204.

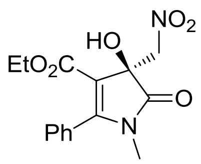

(*S*)-Ethyl 4-hydroxy-1-methyl-4-(nitromethyl)-5-oxo-2-phenyl-4,5-dihydro-1*H*-pyrrole-3-carboxylate (**4c**). Light yellow oil; 12.2 mg, 19% yield; 57% ee; **^{1}H NMR (300 MHz, $CDCl_3$)** δ 7.52-7.45 (m, 3H), 7.33-7.30 (m, 2H), 5.17 (d, *J* = 12.8 Hz, 1H), 5.04 (d, *J* = 12.8 Hz, 1H), 4.11-3.93 (m, 2H), 3.72 (s, 1H), 2.91 (s, 3H), 0.97 (t, *J* = 7.1 Hz, 3H); **^{13}C NMR (75 MHz, $CDCl_3$)** δ 175.1, 162.8, 160.0, 130.5, 128.4, 128.4, 128.3, 106.5, 77.8, 74.0, 60.4, 28.0, 13.6; HPLC (Chiralpak OJ-H column, *i*-PrOH/*n*-hexane = 20/80, flow rate 1.0 mL/min, λ = 254 nm, t_{major} = 10.79 min, t_{minor} = 9.93 min); $[\alpha]_D^{20}$ = +20.6 (*c* 0.990, $CHCl_3$); HRMS (ESI-TOF) calcd for $C_{15}H_{16}N_2NaO_6$ $[M + Na]^+$ 343.0901; found: 343.0897.

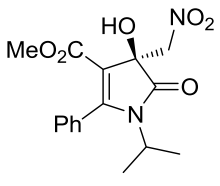

(*S*)-Methyl 4-hydroxy-1-isopropyl-4-(nitromethyl)-5-oxo-2-phenyl-4,5-dihydro-1*H*-pyrrole-3-carboxylate (**4d**). Light yellow oil; 36.7 mg, 55% yield; 65% ee; **^{1}H NMR (300 MHz, $CDCl_3$)** δ 7.52-7.47 (m, 3H), 7.30 (d, *J* = 3.6 Hz, 1H), 7.24 (d, *J* = 5.8 Hz, 1H), 5.17 (d, *J* = 12.8 Hz, 1H), 4.99 (d, *J* = 12.8 Hz, 1H), 3.77 (s, 1H), 3.62-3.55 (m, 1H), 3.52 (s, 3H), 1.38 (d, *J* = 6.9 Hz, 3H), 1.34 (d, *J* = 6.9 Hz, 3H). **^{13}C NMR (75 MHz, $CDCl_3$)** δ 175.5, 163.0, 161.5, 130.2, 129.1, 128.7, 128.5, 128.2, 127.4, 106.2, 77.6, 73.7, 51.3, 47.4, 19.9, 19.0; HPLC (Chiralpak AD-H column, *i*-PrOH/*n*-hexane = 10/90, flow rate 1.0 mL/min, λ = 254 nm, t_{major}= 16.06 min, t_{minor}= 14.68 min); $[\alpha]_D^{20}$ = +21.5 (*c* 2.195, $CHCl_3$)**;** $[\alpha]_D^{20}$ = +2.4 (*c* 2.150, $CHCl_3$); HRMS (ESI-TOF) calcd for $C_{16}H_{18}N_2NaO_6$ $[M + Na]^+$ 357.1057; found: 357.1056.

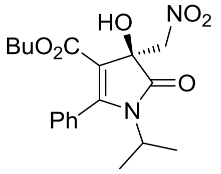

(*S*)-Butyl 4-hydroxy-1-isopropyl-4-(nitromethyl)-5-oxo-2-phenyl-4,5-dihydro-1*H*-pyrrole-3-carboxylate (**4e**). Colourless oil; 39.1 mg, 52% yield; 70% ee; **^{1}H NMR (300 MHz, DMSO)** δ 7.52 (s, 3H), 7.38 (s, 1H), 7.10 (s, 1H), 6.75 (s, 1H), 5.13 (d, *J* = 12.8 Hz, 1H), 5.05 (d, *J* = 12.8 Hz, 1H), 3.80 (t, *J* = 6.1 Hz, 2H), 3.47-3.37 (m, 1H), 1.29 (d, *J* = 6.8 Hz, 3H), 1.22-1.14 (m, 5H), 0.99-0.89 (m, 2H), 0.72 (t, *J* = 7.1 Hz, 3H). **^{13}C NMR (75 MHz, DMSO)** δ 176.3, 161.8, 159.4, 130.0, 129.7, 128.6 (d, *J* = 6.6 Hz), 127.8 (d, *J* = 10.1 Hz), 107.3, 77.7, 73.4, 62.9, 46.3, 29.9, 19.6, 19.0, 18.5, 13.5; HPLC (Chiralpak OD-H column, EtOH/*n*-hexane = 10/90, flow rate 1.0 mL/min, λ = 254 nm, t_{major} = 6.16 min, t_{minor}= 5.22 min); $[\alpha]_D^{20}$ = +11.3 (*c* 1.995, $CHCl_3$)**;** HRMS (ESI-TOF) calcd for $C_{19}H_{24}N_2NaO_6$ $[M + Na]^+$ 399.1527; found: 399.1521.

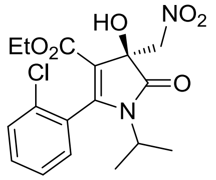

(*S*)-Ethyl 2-(2-chlorophenyl)-4-hydroxy-1-isopropyl-4-(nitromethyl)-5-oxo-4,5-dihydro-1*H*-pyrrole-3-carboxylate (**4f**). Light yellow oil; 40.5 mg, 53% yield; 54:46 dr, 73/60% ee; **^{1}H NMR (300 MHz, $CDCl_3$)** δ 7.52-7.38 (m, 3H), 7.29-7.26 (m, 1H), 5.12 (d, *J* = 12.3 Hz, 1H), 4.99 (d, *J* = 12.3 Hz, 1H), 3.98 (q, *J* = 7.1 Hz, 2H), 3.72 (s, 1H), 3.49-3.39 (m, 1H), 1.47 (d, *J* = 6.9 Hz, 3H), 1.32 (d, *J* = 6.9 Hz, 3H), 0.91 (t, *J* = 7.1 Hz, 3H). **^{13}C NMR (75 MHz, $CDCl_3$)** δ 175.1, 162.3, 157.8, 133.2, 131.4, 129.8, 129.4, 129.1, 126.8, 107.6, 77.4, 73.9, 60.3, 47.9, 20.0, 19.3, 13.6; HPLC (Chiralpak AD-H column, *i*-PrOH/*n*-hexane = 10/90, flow rate 1.0 mL/min, λ = 220 nm, t_{major} = 16.63 min, t_{minor} = 15.88 min; t_{major} = 13.63 min, t_{minor} = 14.88 min); $[\alpha]_D^{20}$ = +37.4 (*c* 1.345, $CHCl_3$); HRMS (ESI-TOF) calcd for $C_{17}H_{19}ClN_2NaO_6$ $[M + Na]^+$ 405.0824; found: 405.0819.

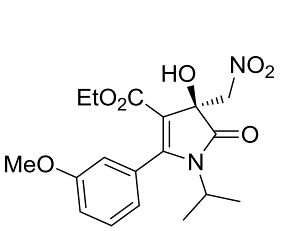

(*S*)-Ethyl 4-hydroxy-1-isopropyl-2-(3-methoxyphenyl)-4-(nitromethyl)-5-oxo-4,5-dihydro-1*H*-pyrrole-3-carboxylate (**4g**). Light yellow oil; 55.2 mg, 73% yield; 71% ee; **^{1}H NMR (300 MHz, $CDCl_3$)** δ 7.42-7.35 (m, 1H), 7.04-7.00 (m, 1H), 6.88-6.74 (m, 2H), 5.16 (d, *J* = 12.8 Hz, 1H), 4.98 (d, *J* = 12.8 Hz, 1H), 4.00-3.94 (m, 2H), 3.83 (s, 3H), 3.72 (s, 1H), 3.64-3.55 (m, 1H), 1.38 (d, *J* = 6.9 Hz, 3H), 1.35 (d, *J* = 6.9 Hz, 3H), 0.95 (t, *J* = 7.1 Hz, 3H). **^{13}C NMR (75 MHz, $CDCl_3$)** δ 175.4, 162.7, 160.8, 159.5 (d, *J* = 8.2 Hz), 130.5, 129.7 (d, *J* = 11.3 Hz), 120.1 (d, *J* = 60.4 Hz), 115.6 (d, *J* = 17.1 Hz), 113.5 (d, *J* = 40.7 Hz), 106.4, 77.6, 73.7, 60.2, 55.4, 47.4, 20.0,

19.1, 13.6. **^{13}C NMR (100 MHz, DMSO, 60 ^{o}C)** δ 176.6, 162.0, 159.7, 131.7, 130.4, 130.4, 120.4, 115.6, 114.1, 108.0, 78.1, 73.9, 59.5, 55.8, 46.8, 20.0, 19.5, 14.0; HPLC (Chiralpak OD-H column, EtOH/*n*-hexane = 10/90, flow rate 1.0 mL/min, λ = 254 nm, t_{major} = 7.87 min, t_{minor} = 6.55 min); $[\alpha]_D^{20}$ = +14.8 (*c* 2.510, $CHCl_3$); HRMS (ESI-TOF) calcd for $C_{18}H_{22}N_2NaO_7$ $[M + Na]^+$ 401.1319; found: 401.1313.

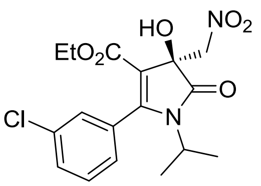

(*S*)-Ethyl 2-(3-chlorophenyl)-4-hydroxy-1-isopropyl-4-(nitromethyl)-5-oxo-4,5-dihydro-1*H*-pyrrole-3-carboxylate (**4h**). Light yellow oil; 35.9 mg, 47% yield; 71% ee; **^{1}H NMR (300 MHz, $CDCl_3$)** δ 7.51-7.43 (m, 2H), 7.30 (d, *J* = 22.4 Hz, 1H), 7.17 (dd, *J* = 18.9, *J* =7.3 Hz, 1H), 5.17 (d, *J* = 12.9 Hz, 1H), 4.98 (d, *J* = 12.9 Hz, 1H), 4.02-3.93 (m, 2H), 3.70 (d, *J* = 5.6 Hz, 1H), 3.62-3.53 (m, 1H), 1.39 (d, *J* = 6.9 Hz, 3H), 1.36 (d, *J* = 6.9 Hz, 3H), 0.95 (t, *J* = 7.1 Hz, 3H). **^{13}C NMR (75 MHz, $CDCl_3$)** δ 175.2, 162.5, 159.2, 134.7 (d, *J* = 13.7 Hz), 131.0 (d, *J* = 3.9 Hz), 130.3 (d, *J* = 1.9 Hz), 129.9 (d, *J* = 13.7 Hz), 128.2 (d, *J* = 58.4 Hz), 126.1 (d, *J* = 56.8 Hz), 107.08, 77.5, 73.7, 60.4, 47.6, 20.0, 19.2 (d, *J* = 3.7 Hz), 13.6; HPLC (Chiralpak OD-H column, EtOH/*n*-hexane = 10/90, flow rate 1.0 mL/min, λ = 254 nm, t_{major} = 7.64 min, t_{minor} = 6.58 min); $[\alpha]_D^{20}$ = +15.6 (*c* 1.985, $CHCl_3$); HRMS (ESI-TOF) calcd for $C_{17}H_{19}ClN_2NaO_6$ $[M + Na]^+$ 405.0824; found: 405.0817.

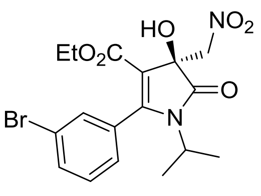

(*S*)-Ethyl 2-(3-bromophenyl)-4-hydroxy-1-isopropyl-4-(nitromethyl)-5-oxo-4,5-dihydro-1*H*-pyrrole-3-carboxylate (**4i**). Light yellow oil; 37.5 mg, 44% yield; 70% ee; **^{1}H NMR (300 MHz, $CDCl_3$)** δ 7.64 (d, *J* = 8.1 Hz, 1H), 7.49 (s, 1H), 7.40-7.33 (m, 1H), 7.21 (dd, *J* = 21.2, *J* = 7.7 Hz, 1H), 5.18 (d, *J* = 13.0 Hz, 1H), 4.98 (d, *J* = 13.0 Hz, 1H), 4.03-3.92 (m, 2H), 3.86 (s, 1H), 3.62- 3.53 (m, 1H) 1.39 (d, *J* = 6.9 Hz, 3H), 1.35 (d, *J* = 6.9 Hz, 3H), 0.94 (t, *J* = 7.1 Hz, 3H). **^{13}C NMR (75 MHz, $CDCl_3$)** δ 175.6, 162.3, 158.9, 133.0 (d, *J* = 4.2 Hz), 131.3 (d, *J* = 5.3 Hz), 130.9 (d, *J* = 33.3 Hz), 130.0 (d, *J* = 11.9 Hz), 126.4 (d, *J* = 35.5 Hz), 122.3 (d, *J* = 11.8 Hz), 107.3, 77.2, 73.6, 60.2, 47.4, 19.8 (d, *J* = 5.0 Hz), 19.0 (d, *J* = 5.0 Hz), 13.4; HPLC (Chiralpak AD-H column, EtOH/*n*-hexane = 10/90, flow rate 1.0 mL/min, λ = 254 nm, t_{major} = 8.24 min, t_{minor} = 7.59 min); $[\alpha]_D^{20}$ = +10.8 (*c* 1.845, $CHCl_3$); HRMS (ESI-TOF) calcd for $C_{17}H_{19}BrN_2NaO_6$ $[M + Na]^+$ 449.0319; found: 449.0305.

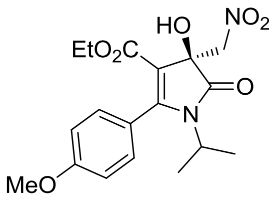

(*S*)-Ethyl 4-hydroxy-1-isopropyl-2-(4-methoxyphenyl)-4-(nitromethyl)-5-oxo-4,5-dihydro-1*H*-pyrrole-3-carboxylate (**4j**). Light yellow oil; 46.1 mg, 61% yield; 72% ee; **^{1}H NMR (300 MHz, $CDCl_3$)** δ 7.26-7.18 (m, 2H), 6.99 (d, *J* = 8.2 Hz, 2H), 5.14 (d, *J* = 12.6 Hz, 1H), 4.98 (d, *J* = 12.6 Hz, 1H), 4.07-3.95 (m, 2H), 3.86 (s, 3H), 3.70- 3.61 (m, 2H), 1.39 (d, *J* = 6.9 Hz, 3H), 1.34 (d, *J* = 6.9 Hz, 3H), 1.00 (t, *J* = 7.1 Hz, 3H). **^{13}C NMR (75 MHz, $CDCl_3$)** δ 175.4, 162.9, 161.3, 161.0, 130.2, 129.1, 121.0, 113.9, 106.3, 77.8, 73.7, 60.2, 55.3, 47.2, 19.9, 19.0, 13.8; HPLC (Chiralpak AD-H column, EtOH/*n*-hexane = 10/90, flow rate 1.0 mL/min, λ = 254 nm, t_{major}= 10.51 min, t_{minor}= 12.42 min); $[\alpha]_D^{20}$ = +6.0 (*c* 2.250, $CHCl_3$); HRMS (ESI-TOF) calcd for $C_{18}H_{22}N_2NaO_7$ $[M + Na]^+$ 401.1319; found: 401.1311.

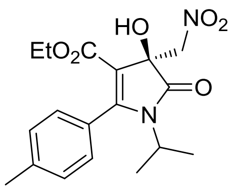

(*S*)-Ethyl 4-hydroxy-1-isopropyl-4-(nitromethyl)-5-oxo-2-(*p*-tolyl)-4,5-dihydro-1*H*-pyrrole-3-carboxylate (**4k**). Colourless oil; 46.3 mg, 64% yield; 69% ee; **^{1}H NMR (300 MHz, $CDCl_3$)** δ 7.29- 7.26 (m, 2H), 7.19-7.12 (m, 2H), 5.15 (d, *J* = 12.7 Hz, 1H), 4.98 (d, *J* = 12.7 Hz, 1H), 4.05-3.94 (m, 2H), 3.66- 3.57 (m, 2H), 2.42 (s, 3H), 1.38 (d, *J* = 6.9 Hz, 3H), 1.34 (d, *J* = 6.9 Hz, 3H), 0.97 (t, *J* = 7.1 Hz, 3H). **^{13}C NMR (75 MHz, $CDCl_3$)** δ 175.4, 162.8, 161.6, 140.4, 129.2, 129.1, 128.4, 127.4, 126.2, 106.3, 77.7, 73.7, 60.2, 47.2, 21.5, 19.9, 19.0, 13.7; HPLC (Chiralpak OD-H column, *i*-PrOH/*n*-hexane = 10/90, flow rate 1.0 mL/min, λ = 254 nm, t_{major} = 6.89 min, t_{minor} = 5.92 min); $[\alpha]_D^{20}$ = +11.3 (*c* 2.595, $CHCl_3$); HRMS (ESI-TOF) calcd for $C_{18}H_{22}N_2NaO_6$ $[M + Na]^+$ 385.1370; found: 385.1363.

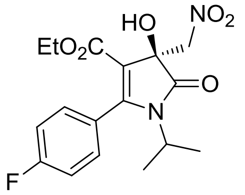

(*S*)-Ethyl 2-(4-fluorophenyl)-4-hydroxy-1-isopropyl-4-(nitromethyl)-5-oxo-4,5-dihydro-1*H*-pyrrole-3-carboxylate (**4l**). Light yellow oil; 53.4 mg, 73% yield; 70% ee; **^{1}H NMR (300 MHz, $CDCl_3$)** δ 7.30-7.15 (m, 4H), 5.17 (d, *J* = 12.8 Hz, 1H), 4.98 (d, *J* = 12.8 Hz, 1H), 4.04-3.93 (m, 2H), 3.78 (s, 1H), 3.60-3.54 (m, 1H), 1.38 (d, *J* = 6.9 Hz, 3H), 1.34 (d, *J* = 6.9 Hz, 3H), 0.96 (t, *J* = 7.1 Hz, 3H). **^{13}C NMR (75 MHz, $CDCl_3$)** δ 175.35, 164.0 (d, *J* = 204.4 Hz), 162.0, 160.0, 130.7 (d, *J* = 8.5 Hz), 129.8 (d, *J* = 8.6 Hz), 125.2 (d, *J* = 3.7 Hz), 116.0 (d, *J* = 9.7 Hz), 115.7 (d, *J* = 9.8 Hz), 107.1, 77.6, 73.7, 60.3, 47.3, 20.0, 19.1, 13.6; HPLC (Chiralpak OD-H column, EtOH/*n*-hexane = 10/90, flow rate 1.0 mL/min, λ = 254 nm, t_{major}= 6.24 min, t_{minor}=

5.69 min); $[\alpha]_D^{20}$ = +12.4 (*c* 1.320, $CHCl_3$); HRMS (ESI-TOF) calcd for $C_{17}H_{19}FN_2NaO_6$ $[M + Na]^+$ 389.1119; found: 389.1114.

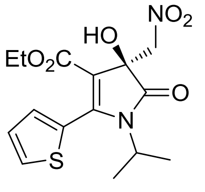

(*S*)-Ethyl 4-hydroxy-1-isopropyl-4-(nitromethyl)-5-oxo-2-(thiophen-2-yl)-4,5-dihydro-1*H*-pyrrole-3-carboxylate (**4m**). Light yellow oil; 36.1 mg, 51% yield; 71% ee; **^{1}H NMR (300 MHz, $CDCl_3$)** δ 7.59 (dd, *J* = 4.2 Hz, *J* =2.1 Hz, 1H), 7.17-7.14 (m, 2H), 5.13 (d, *J* = 12.9 Hz, 1H), 4.98 (d, *J* = 12.9 Hz, 1H), 4.09-4.00 (m, 2H), 3.77-3.70 (m, 1H), 3.52 (s, 1H), 1.43 (d, *J* = 6.9 Hz, 3H), 1.39 (d, *J* = 6.9 Hz, 3H), 1.03 (t, *J* = 7.1 Hz, 3H). **^{13}C NMR (75 MHz, $CDCl_3$)** δ 174.7, 162.6, 154.0, 130.3, 129.1 (d, *J* = 2.6 Hz), 127.7, 127.3 (d, *J* = 2.1 Hz), 109.5, 77.6, 73.8, 60.5, 47.5, 20.0, 19.1, 13.7; HPLC (Chiralpak AD-H column, EtOH/*n*-hexane = 10/90, flow rate 1.0 mL/min, λ = 254 nm, t_{major} = 8.78 min, t_{minor} = 8.03 min); $[\alpha]_D^{20}$ = -3.9 (*c* 1.000, $CHCl_3$); HRMS (ESI-TOF) calcd for $C_{15}H_{18}N_2NaO_6S$ $[M + Na]^+$ 377.0778; found: 377.0772.

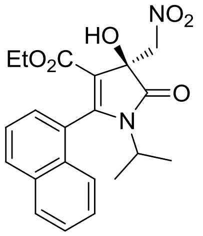

(*S*)-Ethyl 4-hydroxy-1-isopropyl-2-(naphthalen-1-yl)-4-(nitromethyl)-5-oxo-4,5-dihydro-1*H*-pyrrole-3-carboxylate (**4n**). Light yellow oil; 59.7 mg, 75% yield; 53:47 dr, 51/49% ee; **^{1}H NMR (300 MHz, $CDCl_3$)** δ 8.00- 7.92 (m, 2H), 7.75-7.72 (m, 1H), 7.58-7.53 (m, 3H), 7.37-7.35 (m, 1H), 5.25 (d, *J* = 12.9 Hz, 1H), 5.07 (d, *J* = 12.9 Hz, 1H), 3.85 (s, 1H), 3.82-3.72 (m, 2H), 3.37-3.33 (m, 1H), 1.33 (d, *J* = 6.9 Hz, 3H), 1.29 (d, *J* = 6.9 Hz, 3H), 0.53 (t, *J* = 7.1 Hz, 3H). **^{13}C NMR (75 MHz, $CDCl_3$)** δ 175.4, 162.8, 160.1, 133.1, 130.4, 130.3, 128.6, 127.4, 127.0, 126.7, 126.4, 125.1, 124.1, 108.2, 77.5, 73.9, 60.0, 47.7, 20.0, 19.1, 13.1; HPLC (Chiralpak OD-H column, EtOH/*n*-hexane = 3/97, flow rate 1.0 mL/min, λ = 254 nm, t_{major} = 14.89 min, t_{minor} = 11.64 min; t_{major} = 17.12 min, t_{minor} = 13.33 min); $[\alpha]_D^{20}$ = +6.0 (*c* 1.955, $CHCl_3$); HRMS (ESI-TOF) calcd for $C_{21}H_{22}N_2NaO_6$ $[M + Na]^+$ 421.1370; found: 421.1358.

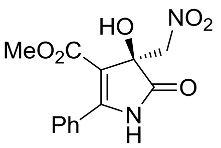

(*S*)-Methyl 4-hydroxy-4-(nitromethyl)-5-oxo-2-phenyl-4,5-dihydro-1*H*-pyrrole-3-carboxylate (**4o**). Light yellow oil; 23.9 mg, 41% yield; 48% ee; **^{1}H NMR (300 MHz, $CDCl_3$)** δ 8.10 (s, 1H), 7.56 -7.43 (m, 5H), 5.14 (d, *J* = 12.9 Hz, 1H), 4.98 (d, *J* = 12.9 Hz, 1H), 3.94 (s, 1H), 3.67 (s, 3H). **^{13}C NMR (75 MHz, $CDCl_3$)** δ 175.2, 163.3, 156.2, 131.7, 128.6, 128.5, 128.4, 105.2, 77.5, 75.3, 51.6; HPLC (Chiralpak AD-H column,

i-PrOH/*n*-hexane = 20/80, flow rate 1.0 mL/min, λ = 254 nm, t_{major} = 10.15 min, t_{minor} = 9.29 min); $[\alpha]_D^{20}$ = +12.0 (*c* 1.135, $CHCl_3$); HRMS (ESI-TOF) calcd for $C_{13}H_{12}N_2NaO_6$ $[M + Na]^+$ 315.0588; found: 315.0584.

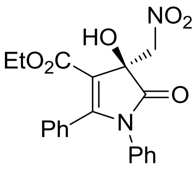

(*S*)-Ethyl 4-hydroxy-4-(nitromethyl)-5-oxo-1,2-diphenyl-4,5-dihydro-1*H*-pyrrole-3-carboxylate (**4p**). Light yellow oil; 32.1 mg, 42% yield; 29% ee; **^{1}H NMR (300 MHz, $CDCl_3$)** δ 7.30-7.22 (m, 6H), 7.18 -7.15 (m, 2H), 7.05-7.02 (m, 2H), 5.29 (d, *J* = 13.0 Hz, 1H), 5.12 (d, *J* = 13.0 Hz, 1H), 4.09 (m, 2H), 3.76 (s, 1H), 1.03 (t, *J* = 7.1 Hz, 3H). **^{13}C NMR (75 MHz, $CDCl_3$)** δ 174.3, 163.1, 159.6, 133.1, 130.2, 129.2, 129.1, 128.4, 128.2, 127.8, 127.7, 106.7, 78.0, 74.2, 60.7, 13.7; HPLC (Chiralpak OD-H column, EtOH/*n*-hexane = 10/90, flow rate 1.0 mL/min, λ = 254 nm, t_{major} = 9.95 min, t_{minor} = 8.77 min); $[\alpha]_D^{20}$ = +8.7 (*c* 1.645, $CHCl_3$); HRMS (ESI-TOF) calcd for $C_{20}H_{18}N_2NaO_6$ $[M + Na]^+$ 405.1057; found: 405.1045.

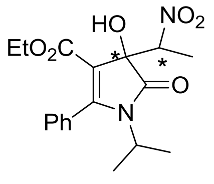

4r

Ethyl 4-hydroxy-1-isopropyl-4-(1-nitroethyl)-5-oxo-2-phenyl-4,5-dihydro-1*H*-pyrrole-3-carboxylate (**4q**). Light yellow oil; 16.7 mg, 23% yield; 94:6 dr, 22% ee; **^{1}H NMR (300 MHz, $CDCl_3$)** δ 7.50-7.44 (m, 3H), 7.26 -7.20 (m, 2H), 5.37 (q, *J* = 7.0 Hz, 1H), 3.98-3.91 (m, 2H), 3.80 (s, 1H), 3.60-3.50 (m, 1H), 1.87 (d, *J* = 7.0 Hz, 3H), 1.36 (d, *J* = 0.9 Hz, 3H), 1.34 (d, *J* = 0.9 Hz, 3H), 0.88 (t, *J* = 7.1 Hz, 3H). **^{13}C NMR (75 MHz, $CDCl_3$)** δ 174.1, 163.0, 160.2, 130.0, 129.4, 128.6, 128.5, 128.3, 127.2, 106.7, 86.8, 75.2, 60.2, 47.2, 20.0, 19.0, 13.5, 12.6; HPLC (Chiralpak AD-H column, EtOH/*n*-hexane = 10/90, flow rate 1.0 mL/min, λ = 254 nm, t_{major} = 7.74 min, t_{minor} = 5.56 min); $[\alpha]_D^{20}$ = -2.5 (*c* 1.470, $CHCl_3$); HRMS (ESI-TOF) calcd for $C_{18}H_{22}N_2NaO_6$ $[M + Na]^+$ 385.1370; found: 385.1373.

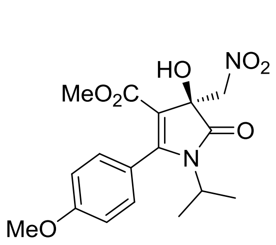

(*S*)-Methyl 4-hydroxy-1-isopropyl-2-(4-methoxyphenyl)-4-(nitromethyl)-5-oxo-4,5-dihydro-1*H*-pyrrole-3-carboxylate (**4r**). Light yellow oil; 37.1 mg, 51% yield; 69% ee; **^{1}H NMR (300 MHz, $CDCl_3$)** δ 7.26-7.17 (m, 2H), 6.99 (d, *J* = 8.4 Hz, 2H), 5.14 (d, *J* = 12.7 Hz, 1H), 4.97 (d, *J* = 12.7 Hz, 1H), 3.86 (s, 3H), 3.69 (s, 1H), 3.68-3.60 (m, 1H), 3.56 (s, 3H), 1.38 (d, *J* = 6.9 Hz, 3H), 1.34 (d, *J* = 6.9 Hz, 3H). **^{13}C NMR (75 MHz, $CDCl_3$)** δ 175.5, 163.2, 161.7, 161.0, 130.1, 129.1, 120.8, 114.0, 106.1, 77.7, 73.7, 55.3, 51.3, 47.2, 19.9, 19.0; HPLC (Chiralpak

AD-H column, EtOH/*n*-hexane = 10/90, flow rate 1.0 mL/min, λ = 254 nm, t_{major} = 10.22 min, t_{minor}= 12.03 min); $[\alpha]_D^{20}$ = +8.2 (*c* 1.780, $CHCl_3$); HRMS (ESI-TOF) calcd for $C_{17}H_{20}N_2NaO_7$ $[M + Na]^+$ 387.1163; found: 387.1159.

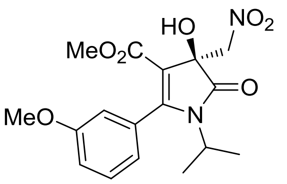

(*S*)-Methyl 4-hydroxy-1-isopropyl-2-(3-methoxyphenyl)-4-(nitromethyl)-5-oxo-4,5-dihydro-1*H*-pyrrole-3-carboxylate (**4s**). Light yellow oil; 51.7 mg, 71% yield; 58% ee; **^{1}H NMR (300 MHz, $CDCl_3$)** δ 7.40-7.37 (m, 1H), 7.05-7.01 (m, 1H), 6.88-6.74 (m, 2H), 5.16 (d, *J* = 12.8 Hz, 1H), 4.98 (d, *J* = 12.8 Hz, 1H), 3.84 (s, 3H), 3.67 (s, 1H), 3.62-3.53 (m, 4H), 1.36 (t, *J* = 7.6 Hz, 6H). **^{13}C NMR (75 MHz, $CDCl_3$)** δ 175.4, 163.0, 161.2, 159.6 (d, *J* = 9.4 Hz), 130.3, 129.9 (d, *J* = 11.7 Hz), 120.0 (d, *J* = 57.2 Hz), 115.7 (d, *J* = 16.0 Hz), 113.4 (d, *J* = 36.5 Hz), 106.1, 77.6, 73.7, 55.4, 51.4, 47.4, 19.9, 19.1 **^{13}C NMR (101 MHz, DMSO, 60 ^{o}C)** δ 176.6, 162.4, 159.9, 159.6, 131.5, 130.5, 120.4, 115.7, 114.0, 107.7, 78.2, 73.9, 55.8, 51.1, 46. 8, 20.0, 19.5; HPLC (Chiralpak OD-H column, EtOH/*n*-hexane = 10/90, flow rate 1.0 mL/min, λ = 254 nm, t_{major}= 7.86 min, t_{minor}= 7.19 min); $[\alpha]_D^{20}$ = +16.4 (*c* 1.340, $CHCl_3$); HRMS (ESI-TOF) calcd for $C_{17}H_{20}N_2NaO_7$ $[M + Na]^+$ 387.1163; found: 387.1130.

Crystal data for 4i

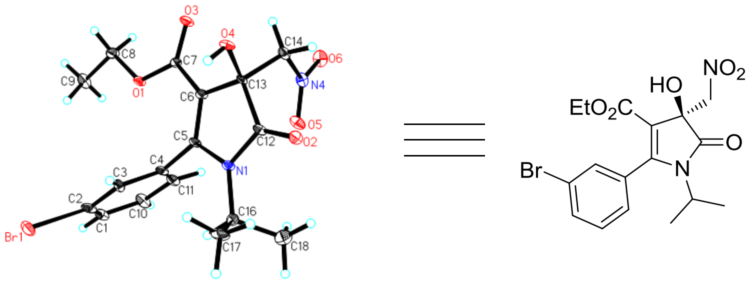

Crystal data and structure refinement for **4i**: (CDCC number: CCDC 1435541)

Identification code mo_zm12_0m

Empirical formula C17 H19 Br N2 O6

Formula weight 427.25

Temperature 100(2) K

Wavelength 0.71073 A

Crystal system, space group Triclinic, P 1

Unit cell dimensions a = 9.380(4) A alpha = 94.969(6) deg.

b = 9.756(4) A beta = 90.378(6) deg.

c = 10.518(5) A gamma = 103.743(6) deg.

Volume 931.1(7) A^3

Z, Calculated density 2, 1.524 Mg/m^3

Absorption coefficient 2.243 mm^-1

F(000) 436

Crystal size 0.74 x 0.26 x 0.05 mm

Theta range for data collection 1.94 to 28.35 deg.

Limiting indices -12<=h<=12, -12<=k<=12, -13<=l<=14

Reflections collected / unique 11667 / 8465 [R(int) = 0.0527]

Completeness to theta = 28.35 98.4 %

Absorption correction Semi-empirical from equivalents

Max. and min. transmission 0.8961 and 0.2876

Refinement method Full-matrix least-squares on F^2

Data / restraints / parameters 8465 / 27 / 476

Goodness-of-fit on F^2 1.004

Final R indices [I>2sigma(I)] R1 = 0.0742, wR2 = 0.1870

R indices (all data) R1 = 0.1035, wR2 = 0.2053

Absolute structure parameter 0.054 (13)

Largest diff. peak and hole 2.249 and -1.301 e.A^-3

References

1. Sano, T.; Horiguchi, Y.; Toda, J.; Imafuku, K.; Tsuda, Y. *Chem. Pharm. Bull.* **1984**, *32*, 497-503. doi.org/10.1248/cpb.32.497

2. Zheng, H.-J.; Chen, W.-B.; Wu, Z.-J.; Deng, J.-G.; Lin, W.-Q.; Yuan, W.-C.; Zhang, X.-M. *Chem. - Eur. J.* **2008**, *14*, 9864-9867. doi: 10.1002/chem.200801582

Copies of NMR and HPLC Spectra for compounds 4

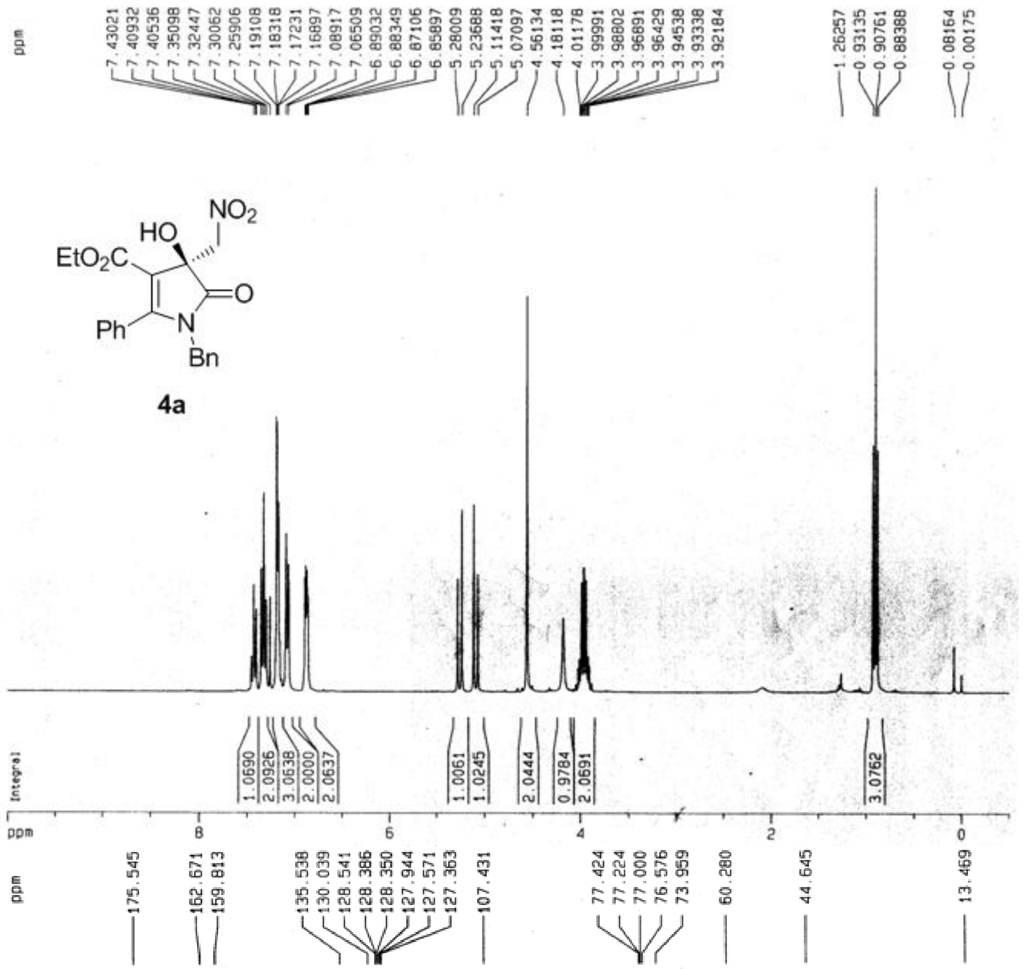

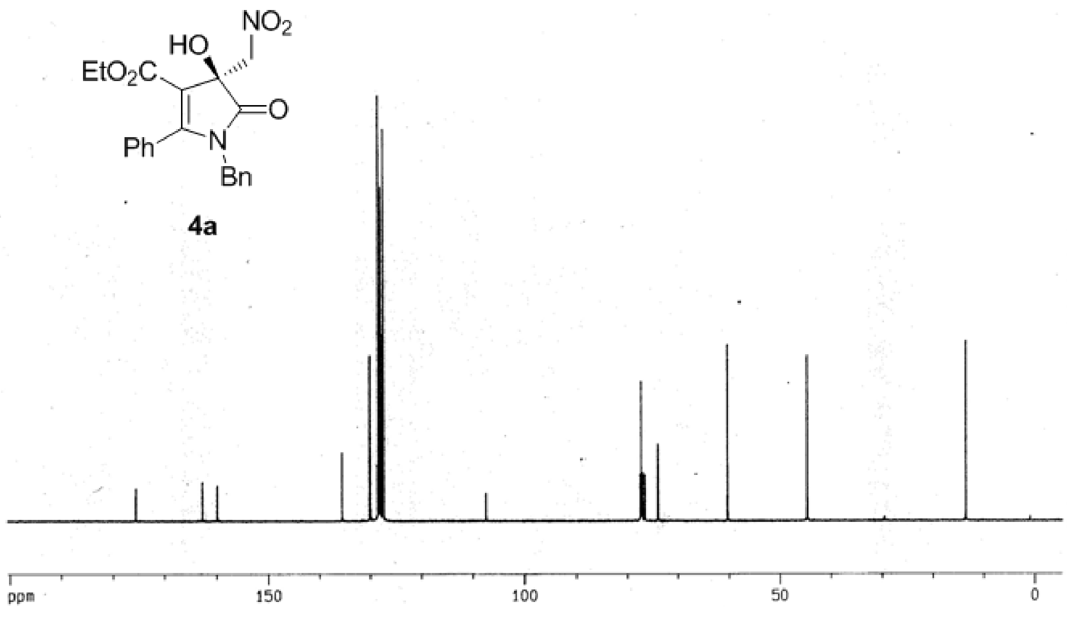

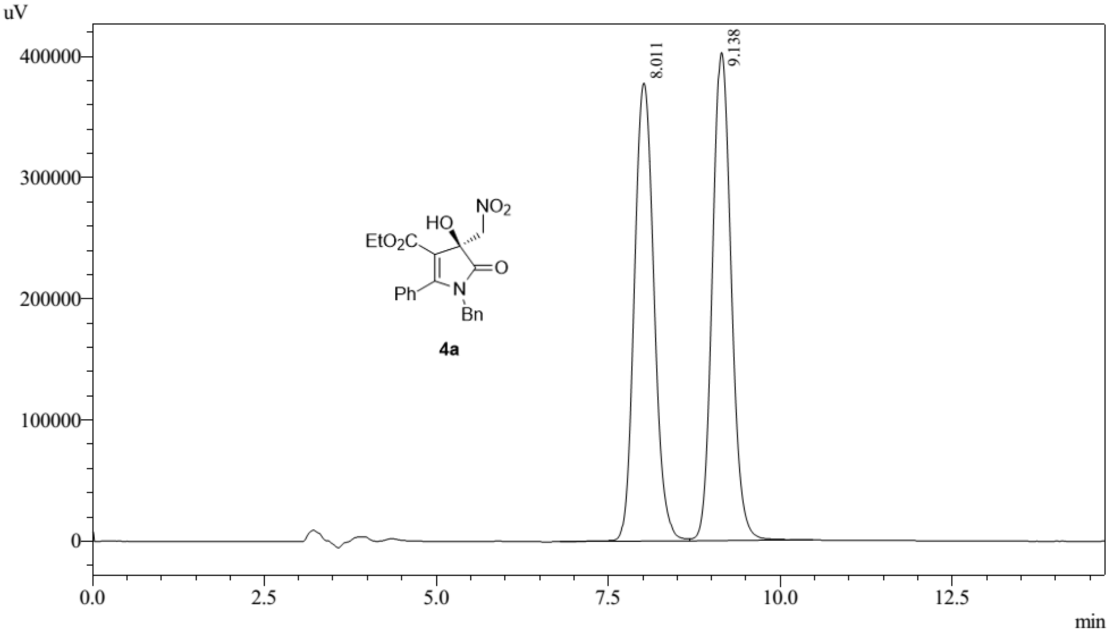

1 Det.A Ch1 / 254nm

Detector A Ch1 254nm

Peak#	Ret. Time	Area	Height	Area %	Height %
1	8.011	7596927	378452	49.819	48.420
2	9.138	7652217	403147	50.181	51.580
Total		15249144	781599	100.000	100.000

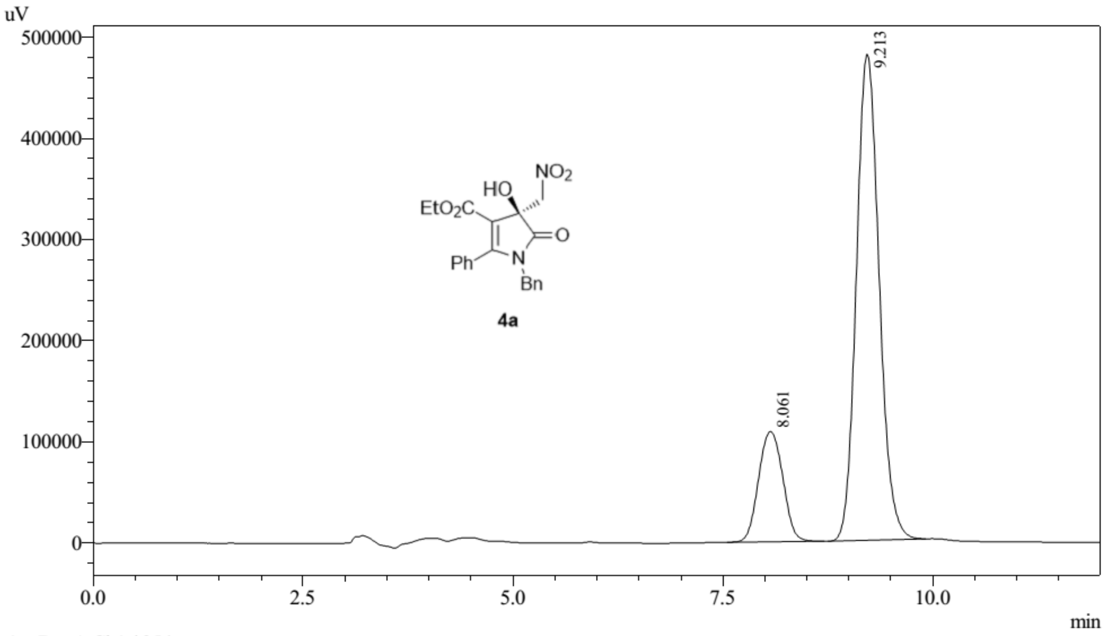

1 Det.A Ch1 / 254nm

Detector A Ch1 254nm

Peak#	Ret. Time	Area	Height	Area %	Height %
1	8.061	2187534	109009	19.457	18.480
2	9.213	9055527	480864	80.543	81.520
Total		11243061	589873	100.000	100.000

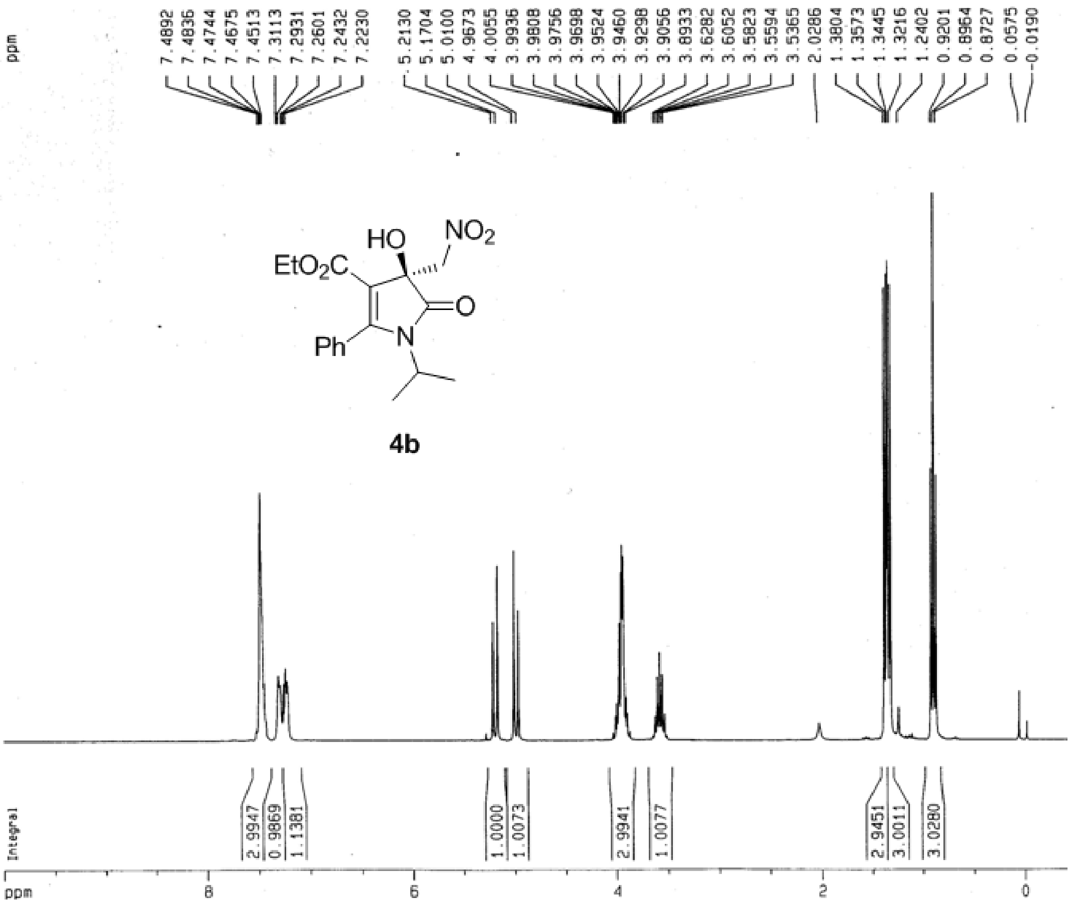
HO
NO2
EtO2C
O
Ph
N
4b
Integral
ppm

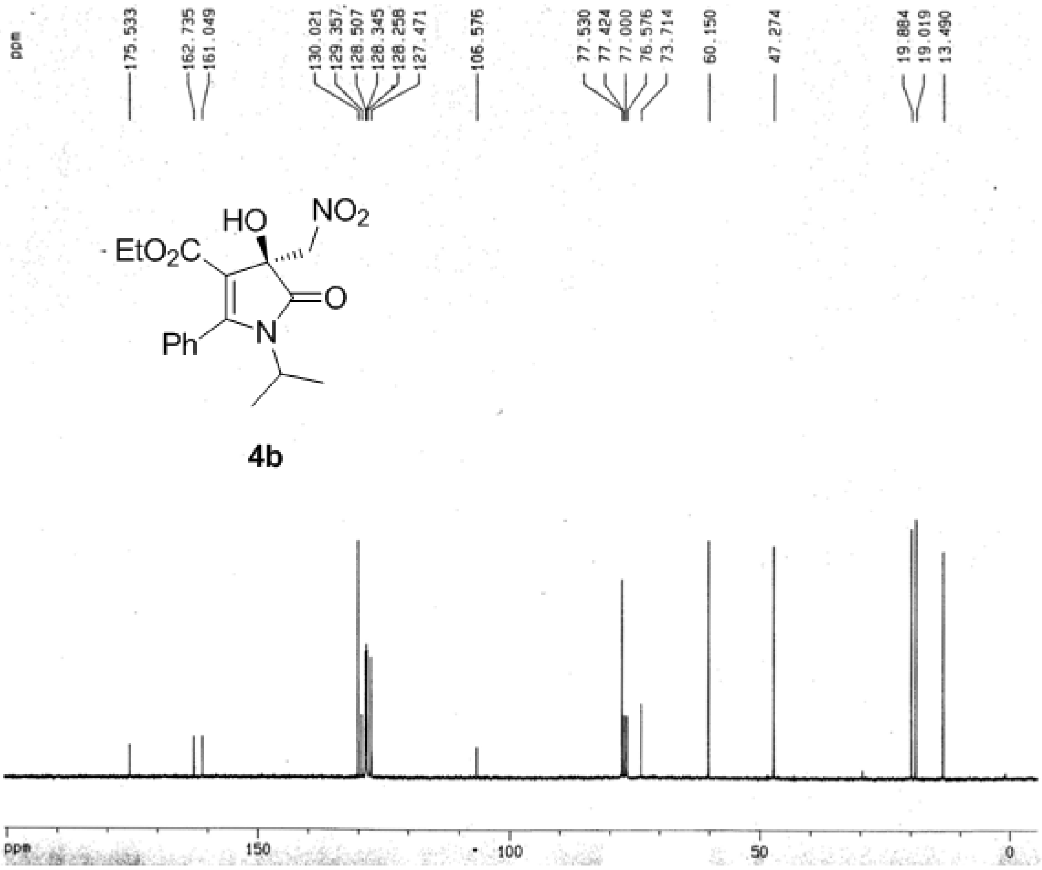
HO
NO2
EtO2C
O
Ph
N
4b
ppm

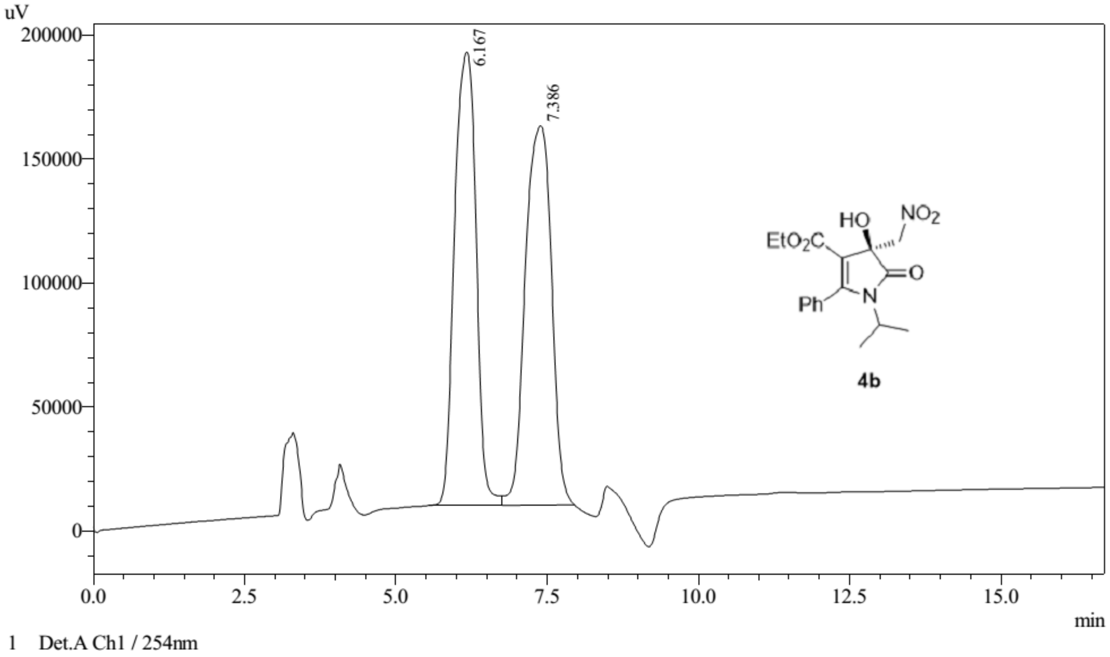

Detector A Ch1 254nm

Peak#	Ret. Time	Area	Height	Area %	Height %
1	6.167	4547674	182845	49.658	54.443
2	7.386	4610324	153004	50.342	45.557
Total		9157998	335849	100.000	100.000

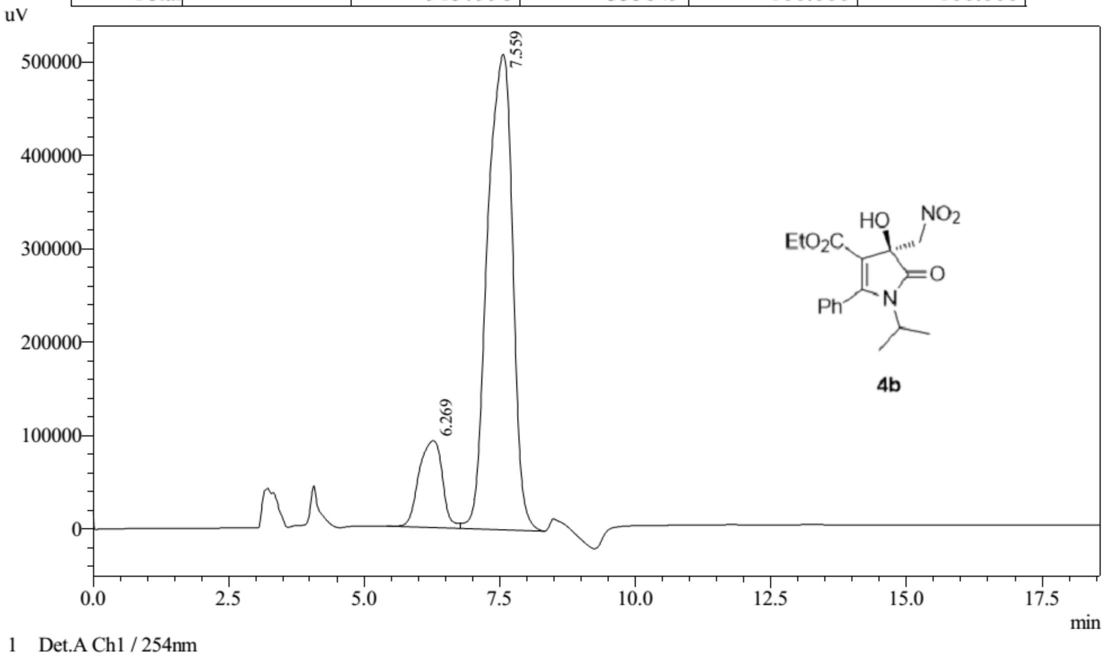

Detector A Ch1 254nm

Peak#	Ret. Time	Area	Height	Area %	Height %
1	6.269	2800755	93113	14.524	15.451
2	7.559	16483100	509522	85.476	84.549
Total		19283855	602636	100.000	100.000

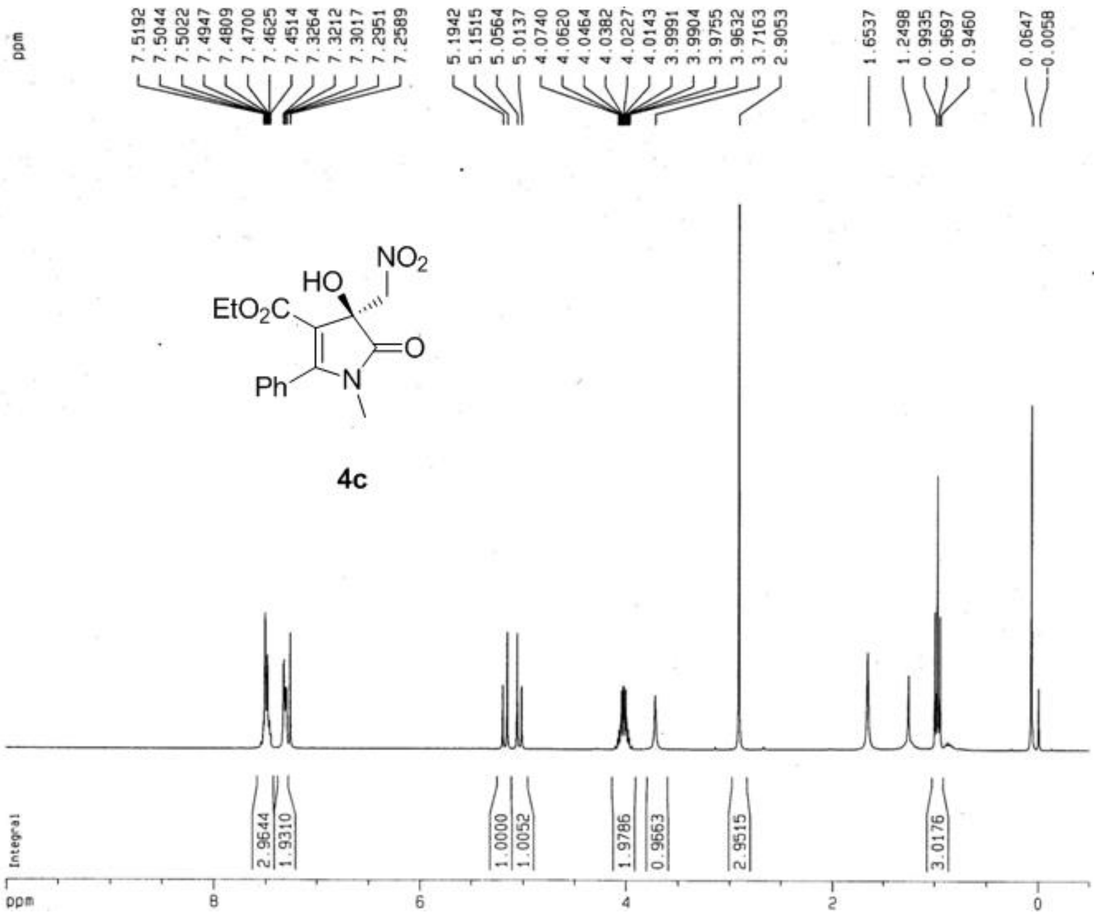
ppm
7.5192 7.5044 7.5022 7.4947 7.4809 7.4700 7.4625 7.4514 7.3264 7.3212 7.3017 7.2951 7.2589
5.1942 5.1515 5.0564 5.0137 4.0740 4.0620 4.0464 4.0382 4.0227 4.0143 3.9991 3.9904 3.9755 3.9632 3.7163 2.9053
1.6537 1.2498 0.9935 0.9697 0.9460 0.0647 -0.0058
EtO2C
HO
NO2
O
Ph
N
4c
Integral
2.9644 1.9310 1.0000 1.0052 1.9786 0.9663 2.9515 3.0176
ppm
8
6
4
2
0

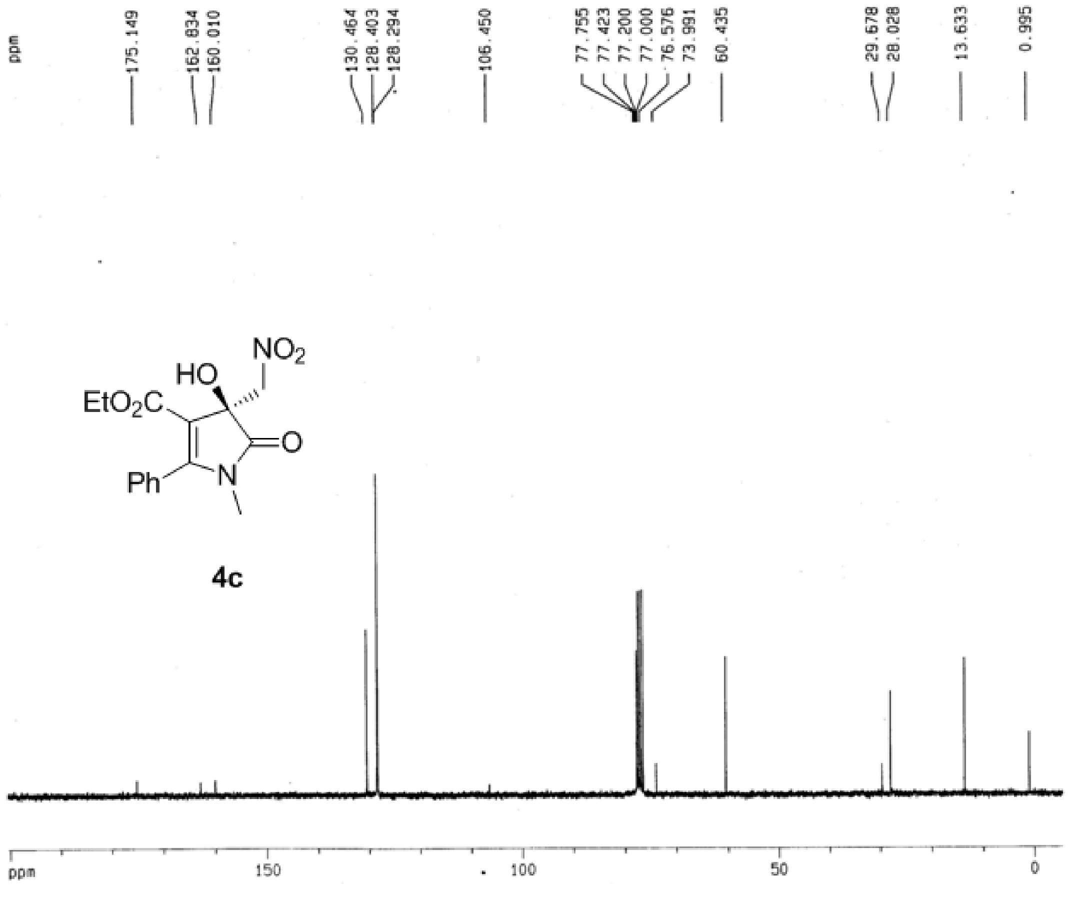
ppm
175.149 162.834 160.010 130.464 128.403 128.294 106.450 77.755 77.423 77.200 77.000 76.576 73.991 60.435 29.678 28.028 13.633 0.995
EtO2C
HO
NO2
O
Ph
N
4c
ppm
150
100
50
0

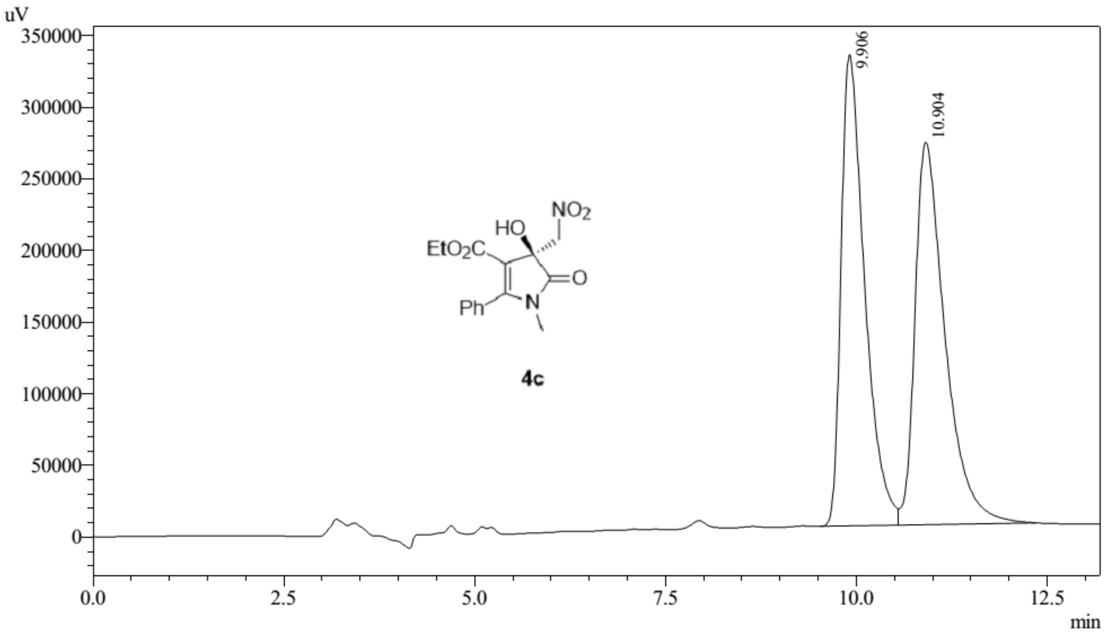

1 Det.A Ch1 / 254nm

Detector A Ch1 254nm

Peak#	Ret. Time	Area	Height	Area %	Height %
1	9.906	7095968	329036	49.288	55.213
2	10.904	7301042	266907	50.712	44.787
Total		14397010	595943	100.000	100.000

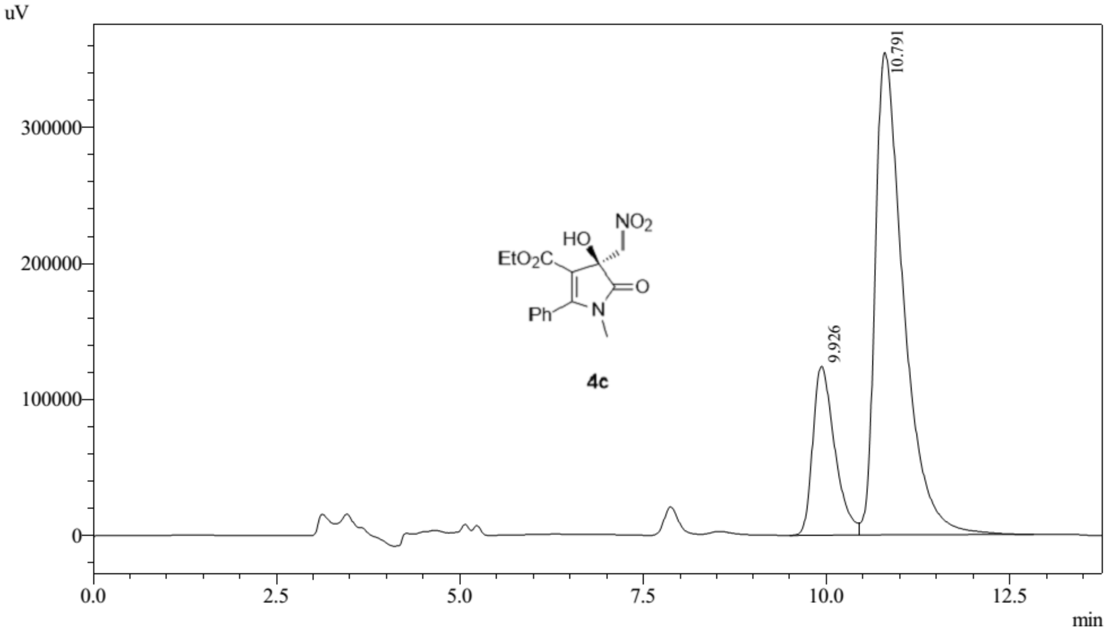

1 Det.A Ch1 / 254nm

Detector A Ch1 254nm

Peak#	Ret. Time	Area	Height	Area %	Height %
1	9.926	2594358	124350	21.323	25.938
2	10.791	9572698	355066	78.677	74.062
Total		12167057	479416	100.000	100.000

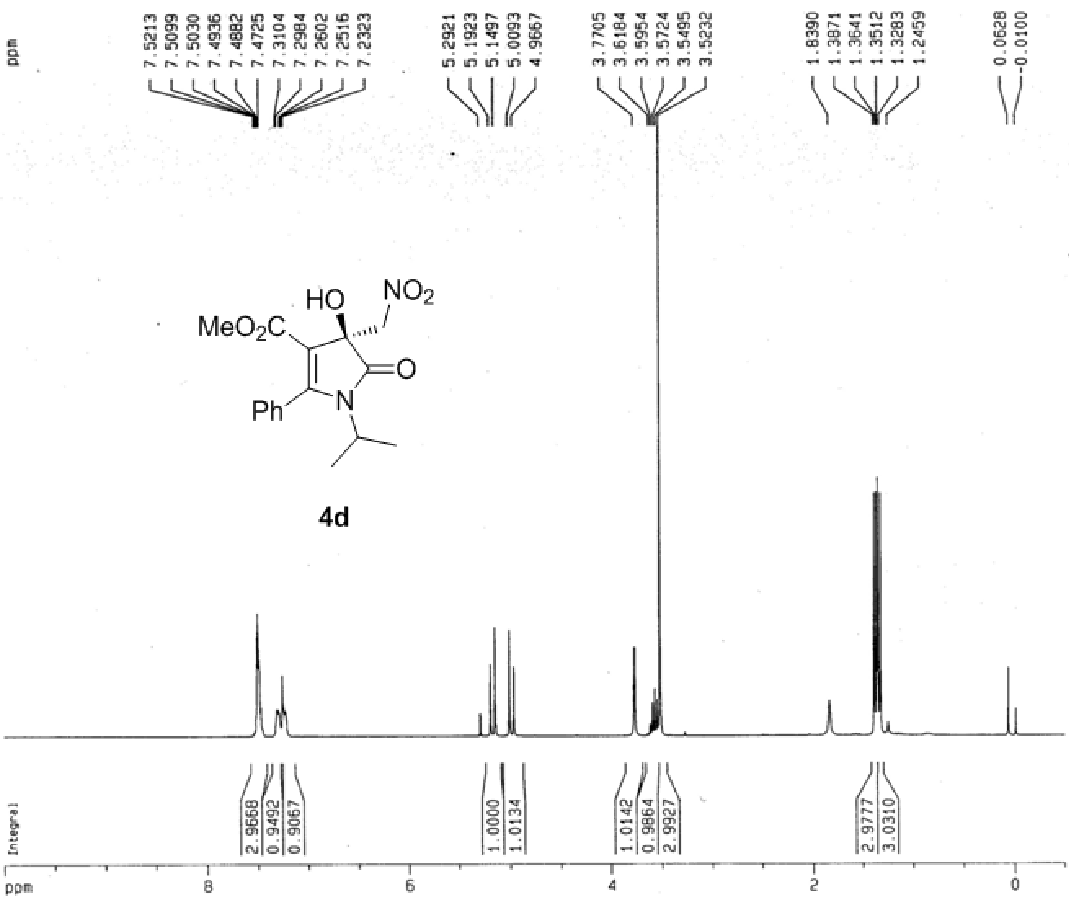
ppm
7.5213
7.5099
7.5030
7.4936
7.4882
7.4725
7.3104
7.2984
7.2602
7.2516
7.2323
5.2921
5.1923
5.1497
5.0093
4.9667
3.7705
3.6184
3.5954
3.5724
3.5495
3.5232
1.8390
1.3871
1.3641
1.3512
1.3283
1.2459
0.0628
-0.0100
HO
NO2
MeO2C
O
N
Ph
4d
Integral
2.9668
0.9492
0.9067
1.0000
1.0134
1.0142
0.9864
2.9927
2.9777
3.0310
ppm
8
6
4
2
0

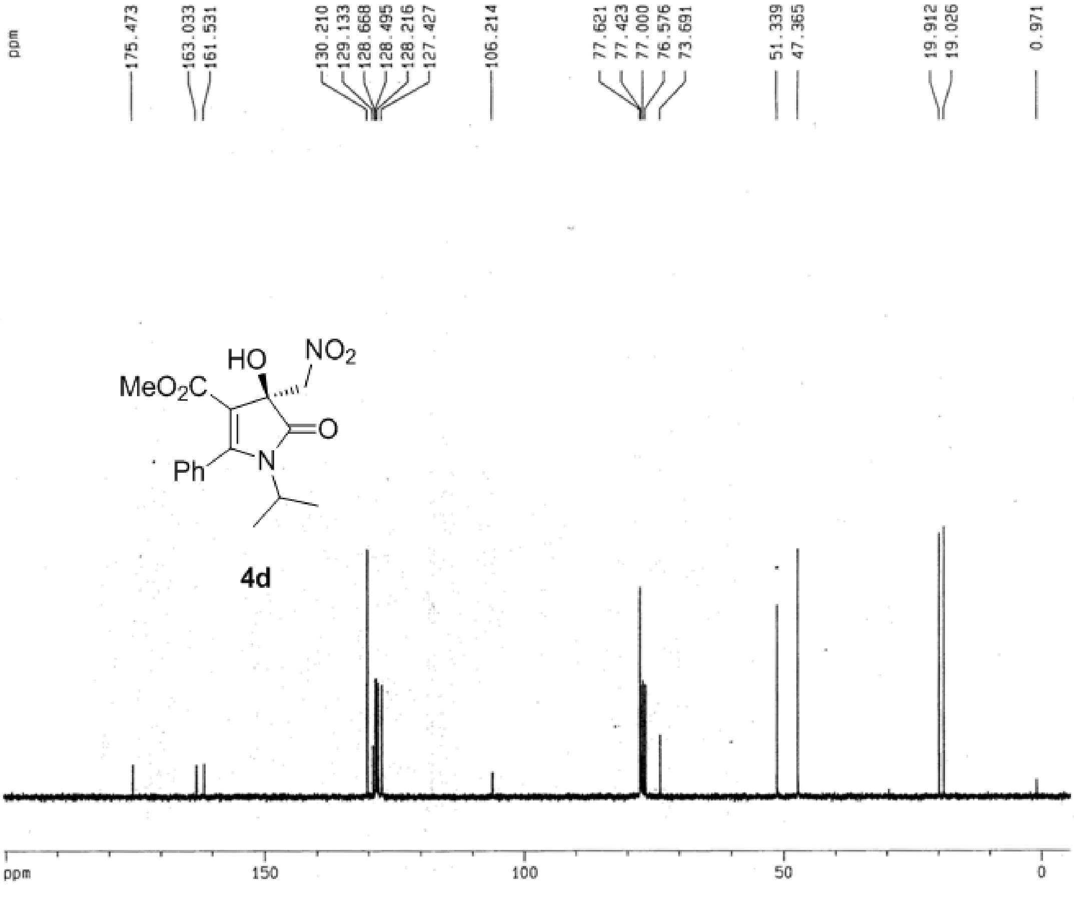
ppm
175.473
163.033
161.531
130.210
129.133
128.668
128.495
128.216
127.427
106.214
77.621
77.423
77.000
76.576
73.691
51.339
47.365
19.912
19.026
0.971
HO
NO2
MeO2C
O
N
Ph
4d
ppm
150
100
50
0

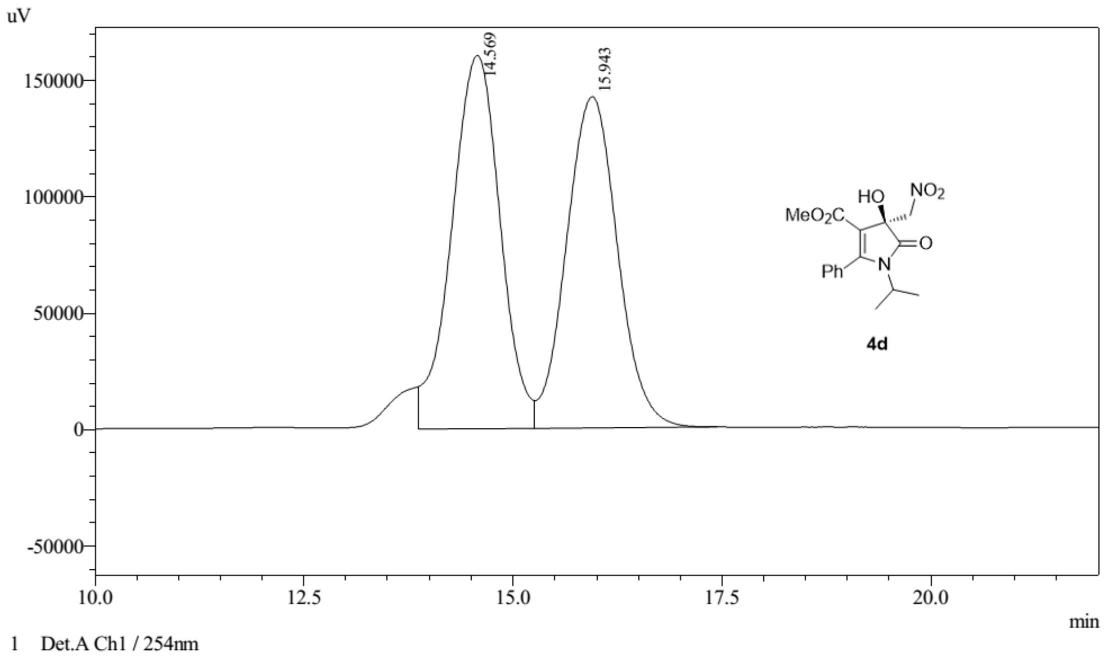

Detector A Ch1 254nm

Peak#	Ret. Time	Area	Height	Area %	Height %
1	14.569	6483423	160396	51.700	52.976
2	15.943	6057078	142375	48.300	47.024
Total		12540500	302772	100.000	100.000

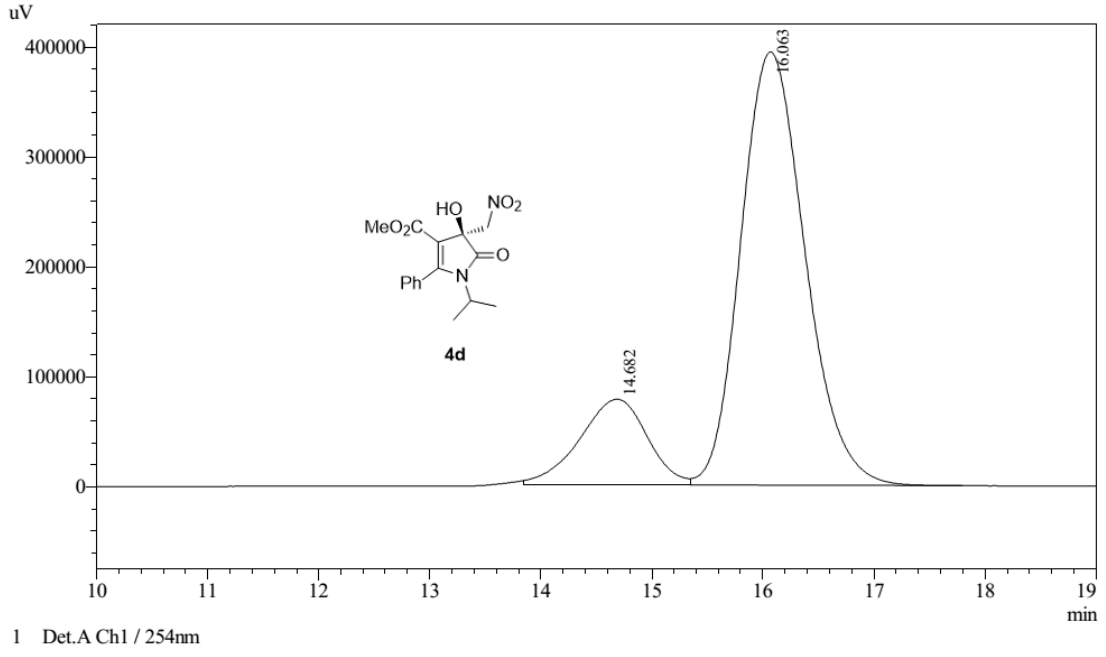

Detector A Ch1 254nm

Peak#	Ret. Time	Area	Height	Area %	Height %
1	14.682	3317243	78064	17.290	16.527
2	16.063	15868387	394267	82.710	83.473
Total		19185630	472331	100.000	100.000

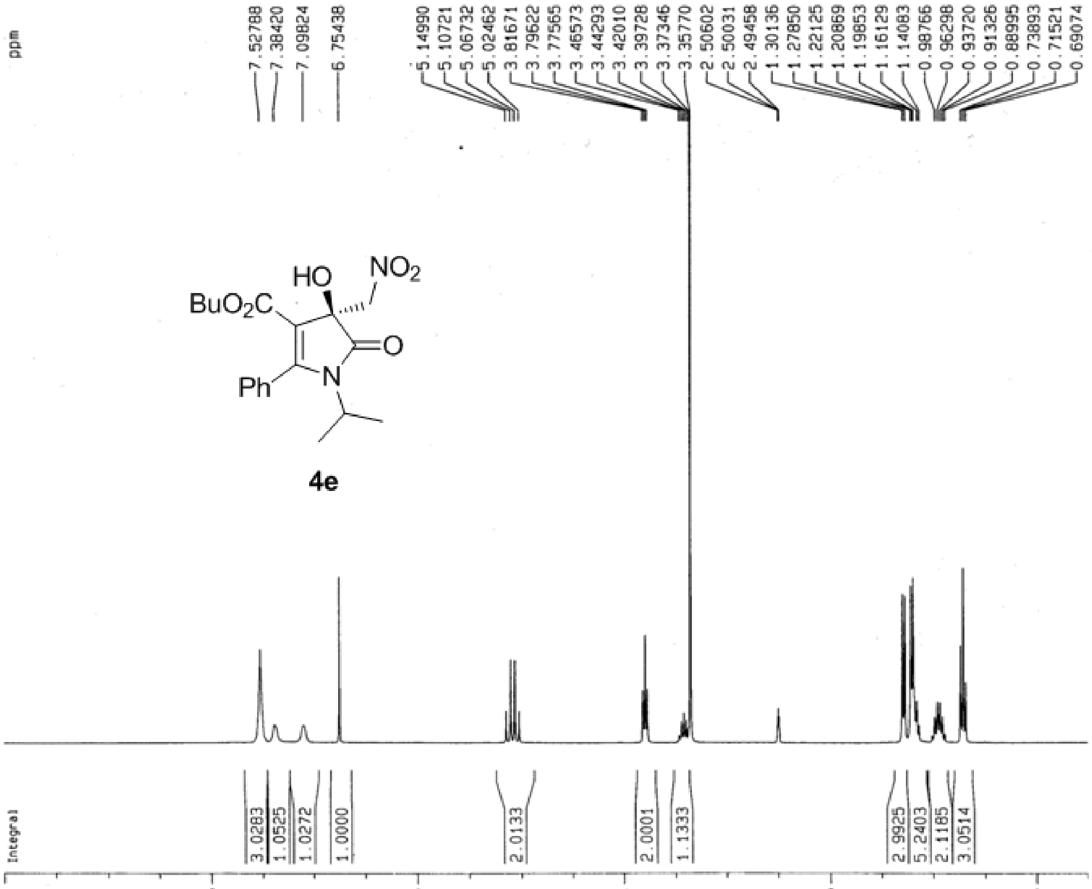
ppm
7.52788
7.38420
7.09824
6.75438
5.14990
5.10721
5.06732
5.02462
3.81671
3.79622
3.77565
3.46573
3.44293
3.42010
3.39728
3.37346
3.35770
2.50602
2.50031
2.49458
1.30136
1.27850
1.22125
1.20869
1.19853
1.16129
1.14083
0.98766
0.96298
0.93720
0.91326
0.88995
0.73893
0.71521
0.69074
4e
Integral
3.0283
1.0525
1.0272
1.0000
2.0133
2.0001
1.1333
2.9925
5.2403
2.1185
3.0514
ppm
8
6
4
2
0

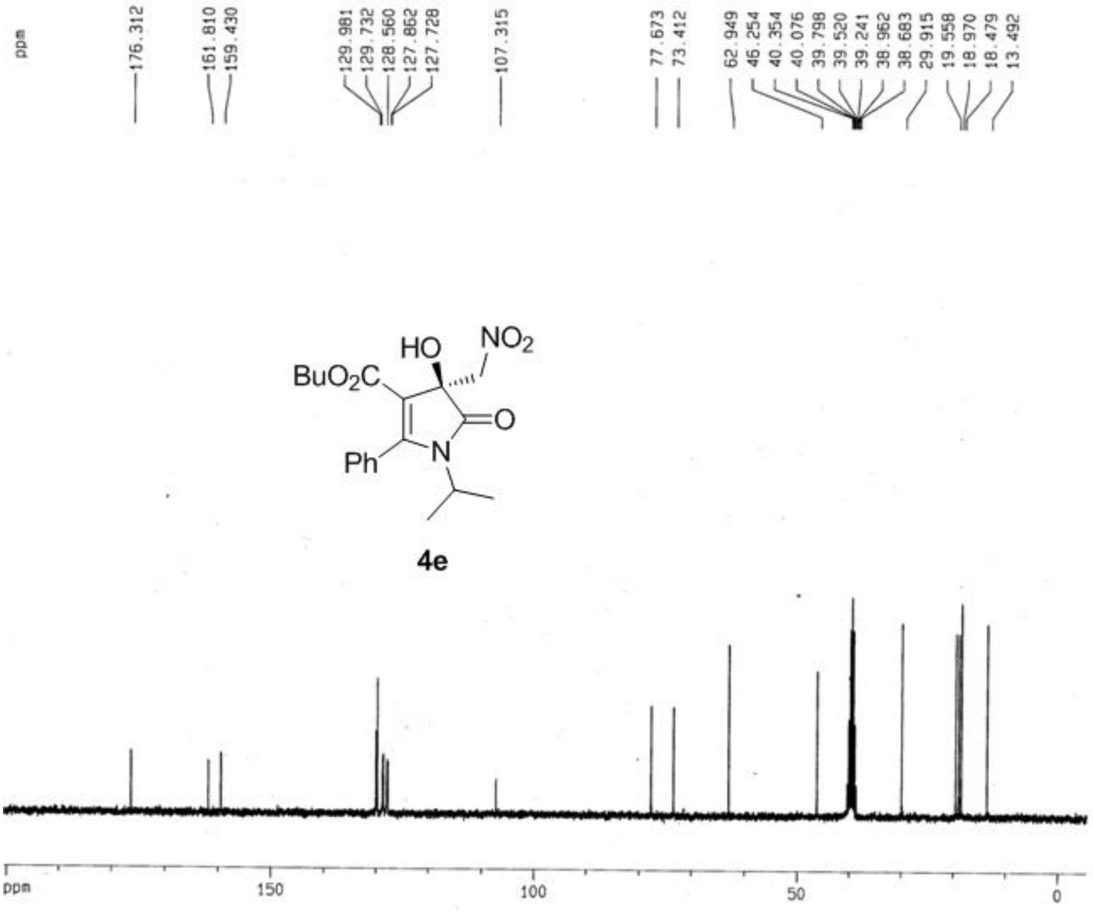
ppm
176.312
161.810
159.430
129.981
129.732
128.560
127.862
127.728
107.315
77.673
73.412
62.949
46.254
40.354
40.076
39.798
39.520
39.241
38.962
38.683
29.915
19.558
18.970
18.479
13.492
4e
ppm
150
100
50
0

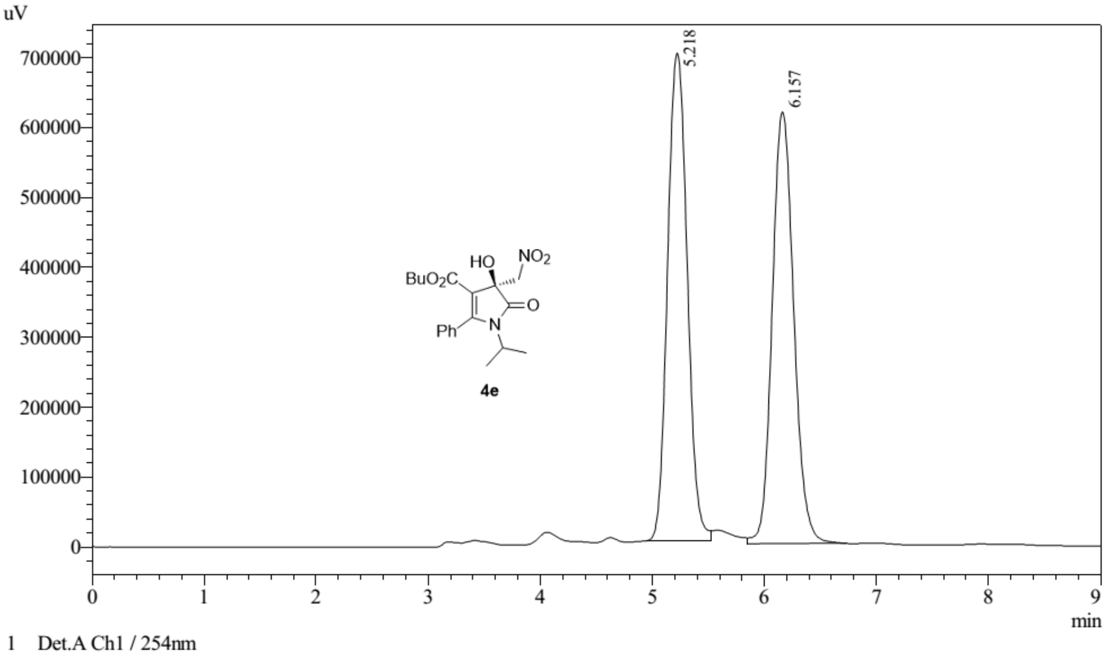

Detector A Ch1 254nm

Peak#	Ret. Time	Area	Height	Area %	Height %
1	5.218	8514398	698407	50.772	53.043
2	6.157	8255408	618271	49.228	46.957
Total		16769806	1316678	100.000	100.000

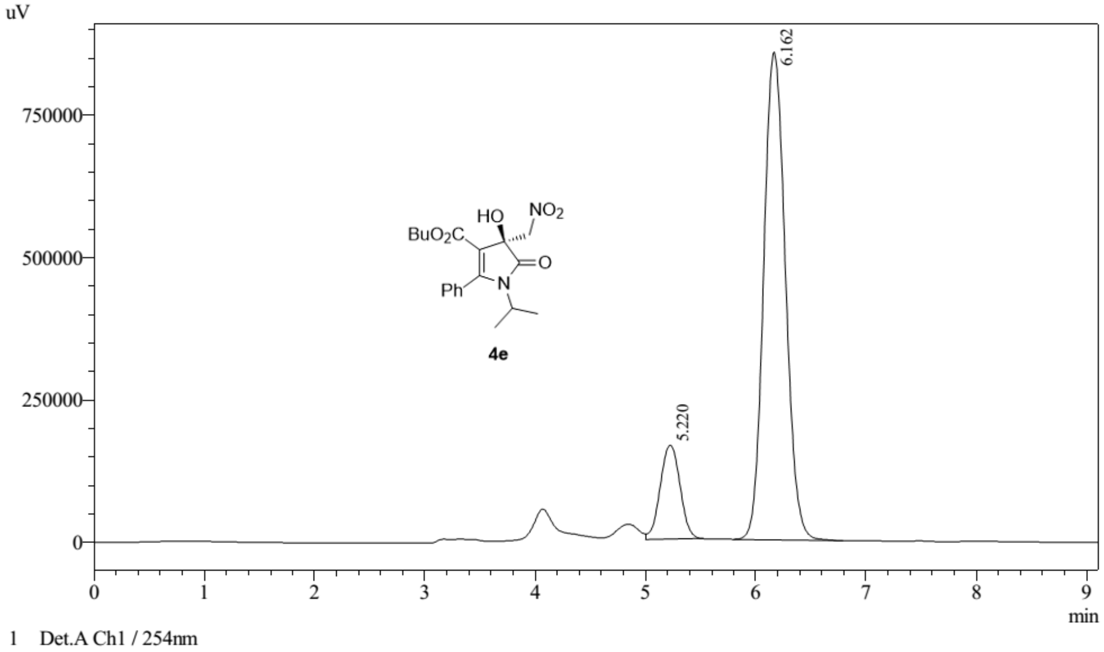

Detector A Ch1 254nm

Peak#	Ret. Time	Area	Height	Area %	Height %
1	5.220	2065178	164749	14.971	16.119
2	6.162	11729794	857350	85.029	83.881
Total		13794972	1022099	100.000	100.000

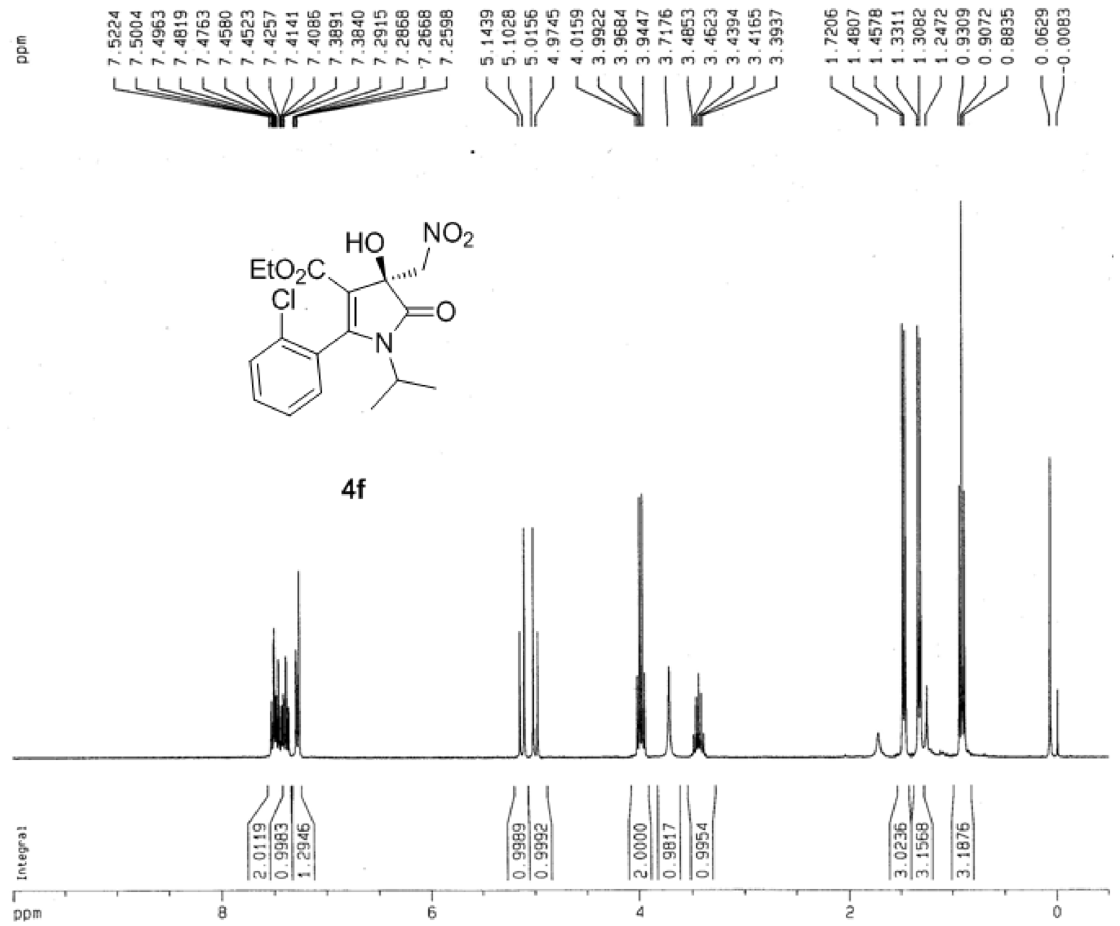
ppm
7.5224
7.5004
7.4963
7.4819
7.4763
7.4580
7.4523
7.4257
7.4141
7.4086
7.3891
7.3840
7.2915
7.2868
7.2668
7.2598
5.1439
5.1028
5.0156
4.9745
4.0159
3.9922
3.9684
3.9447
3.7176
3.4853
3.4623
3.4394
3.4165
3.3937
1.7206
1.4807
1.4578
1.3311
1.3082
1.2472
0.9309
0.9072
0.8835
0.0629
-0.0083
HO
NO_2
EtO_2C
Cl
O
N
4f
Integral
2.0119
0.9983
1.2946
0.9989
0.9992
2.0000
0.9817
0.9954
3.0236
3.1568
3.1876
ppm
8
6
4
2
0

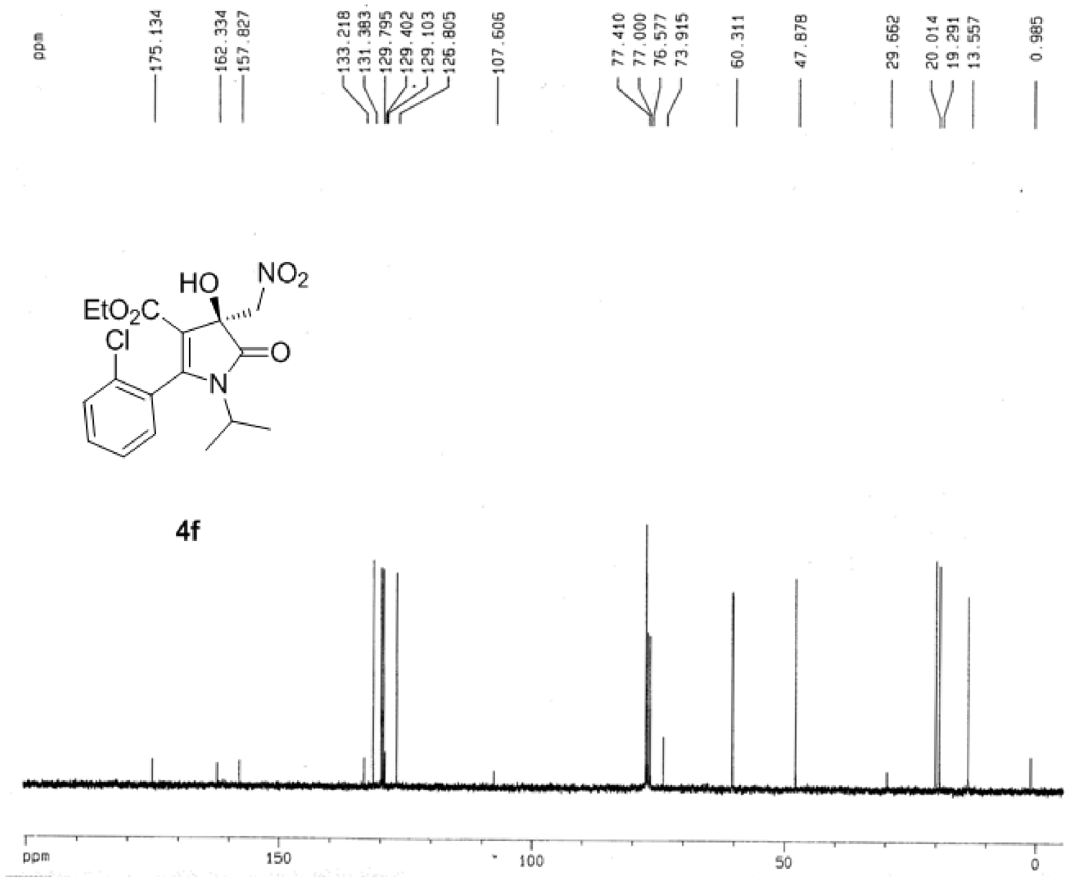
ppm
175.134
162.334
157.827
133.218
131.383
129.795
129.402
129.103
126.805
107.506
77.410
77.000
76.577
73.915
60.311
47.878
29.662
20.014
19.291
13.557
0.985
HO
NO_2
EtO_2C
Cl
O
N
4f
ppm
150
100
50
0

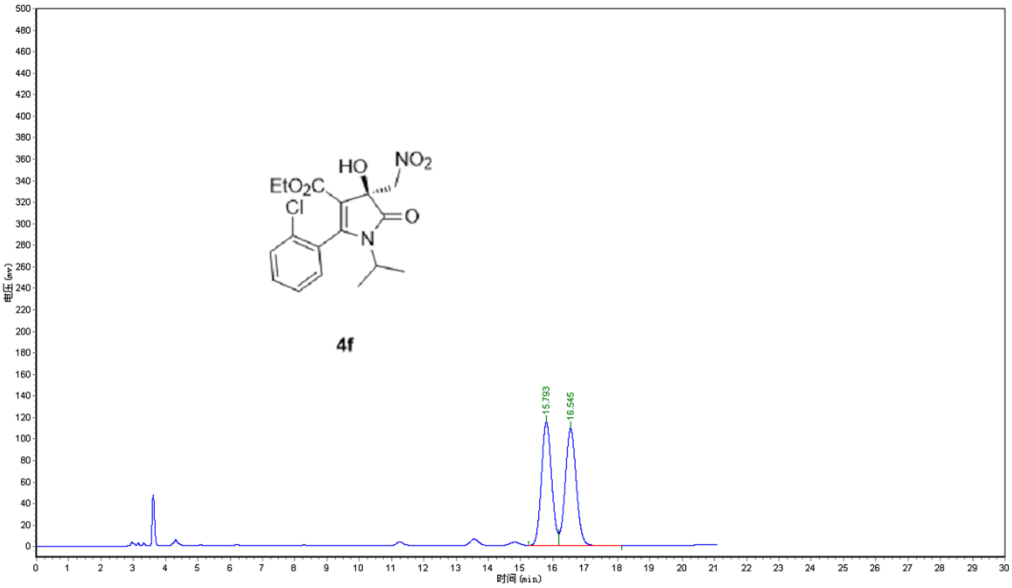

Peak#	Ret. Time	Area	Height	Area %	Height %
1	15.793	2526267	114312	49.797	51.276
2	16.545	2546849	108624	50.203	48.724
Total		5073116	222936	100.000	100.000

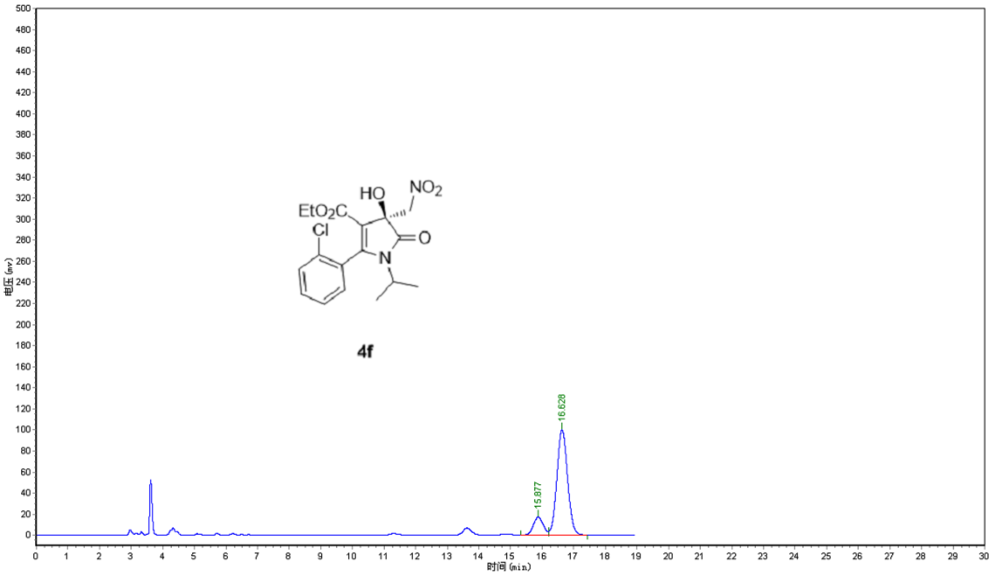

Peak#	Ret. Time	Area	Height	Area %	Height %
1	15.877	369338	17009	13.662	14.572
2	16.628	2334019	99716	86.338	85.428
Total		2703357	116725	100.000	100.000

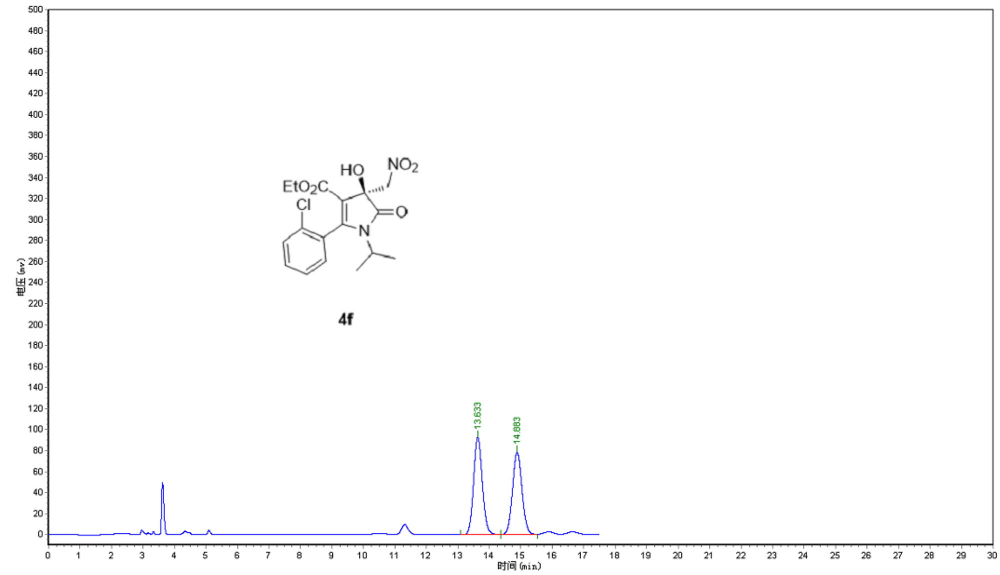

Peak#	Ret. Time	Area	Height	Area %	Height %
1	13.633	1790298	92735	52.005	54.220
2	14.883	1652242	78301	47.995	45.780
Total		3442540	171036	100.000	100.000

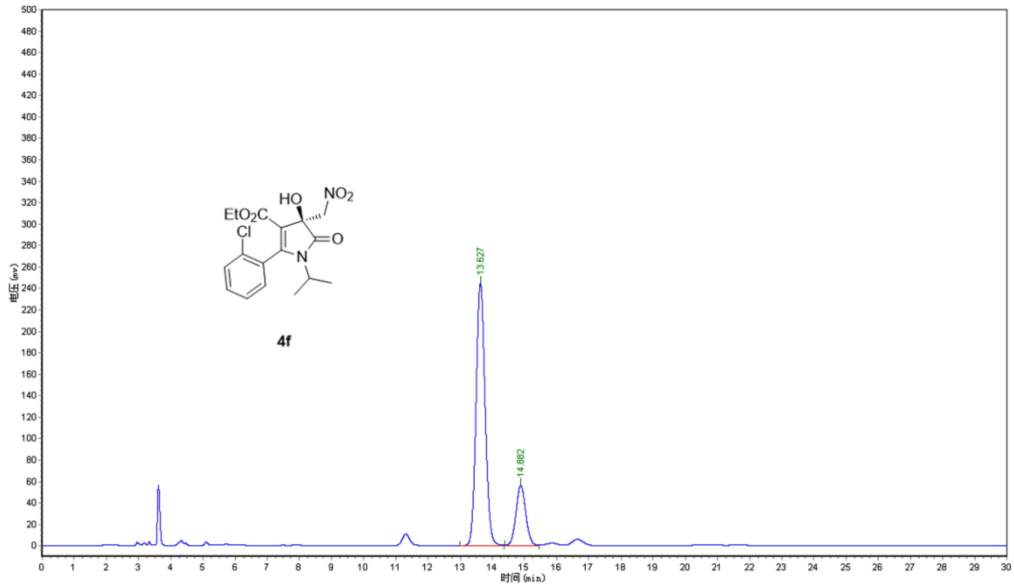

Peak#	Ret. Time	Area	Height	Area %	Height %
1	13.627	4764456	244531	80.111	81.390
2	14.882	1182840	55913	19.889	18.610
Total		5947296	300444	100.000	100.000

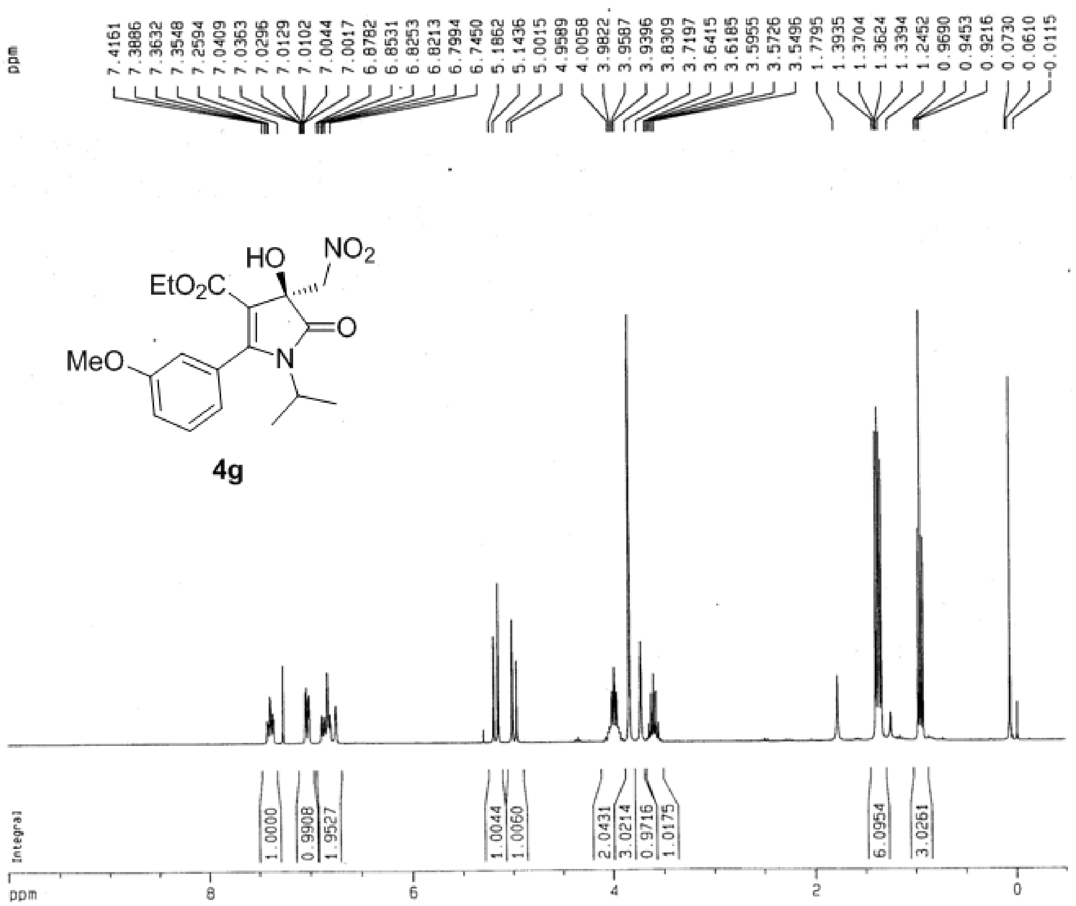
ppm
7.4161
7.3886
7.3632
7.3548
7.2594
7.0409
7.0363
7.0296
7.0129
7.0102
7.0044
7.0017
6.8782
6.8531
6.8253
6.8213
6.7994
6.7450
5.1862
5.1436
5.0015
4.9589
4.0058
3.9822
3.9587
3.9396
3.8309
3.7197
3.6415
3.6185
3.5955
3.5726
3.5496
1.7795
1.3935
1.3704
1.3624
1.3394
1.2452
0.9690
0.9453
0.9216
0.0730
0.0610
-0.0115
HO
NO2
EtO2C
O
MeO
N
4g
Integral
1.0000
0.9908
1.9527
1.0044
1.0060
2.0431
3.0214
0.9716
1.0175
6.0954
3.0261
ppm
8
6
4
2
0

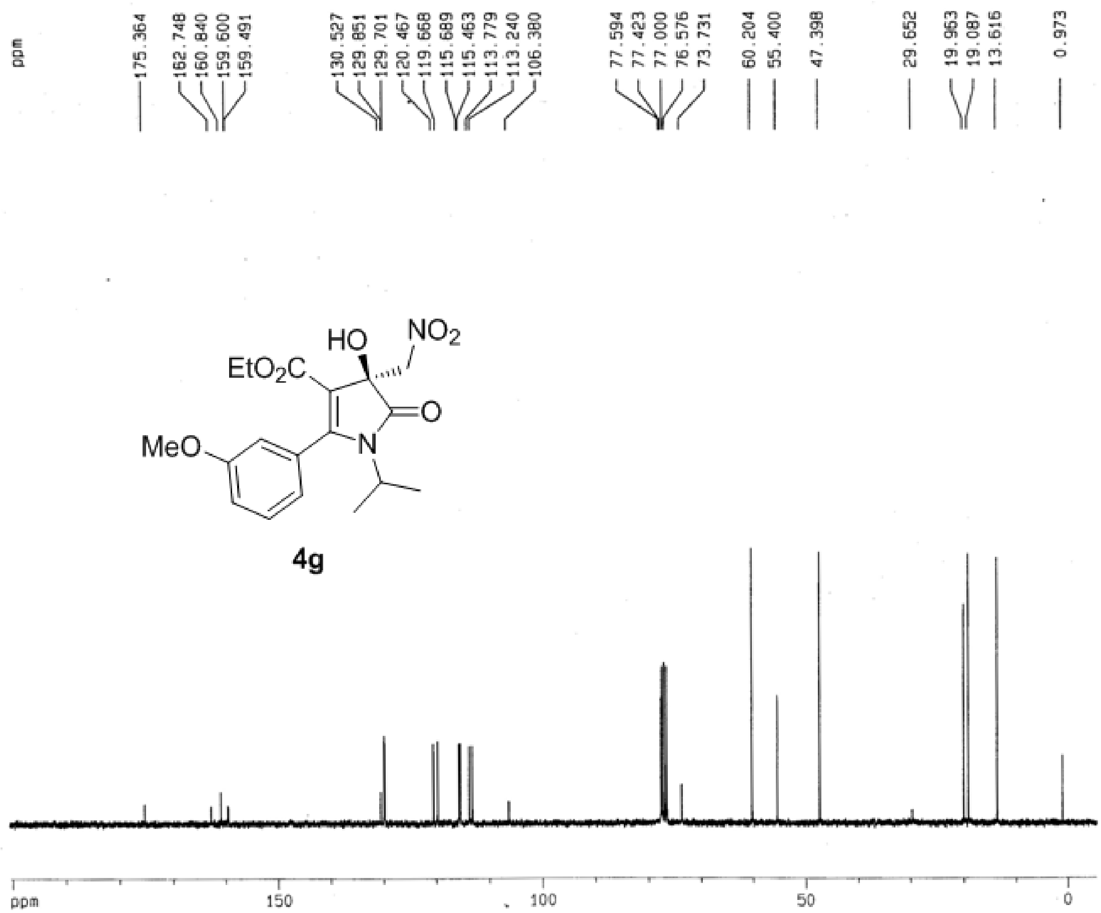
ppm
175.364
162.748
160.840
159.600
159.491
130.527
129.851
129.701
120.467
119.668
115.689
115.463
113.779
113.240
106.380
77.594
77.423
77.000
76.576
73.731
60.204
55.400
47.398
29.652
19.963
19.087
13.616
0.973
HO
NO2
EtO2C
O
MeO
N
4g
ppm
150
100
50
0

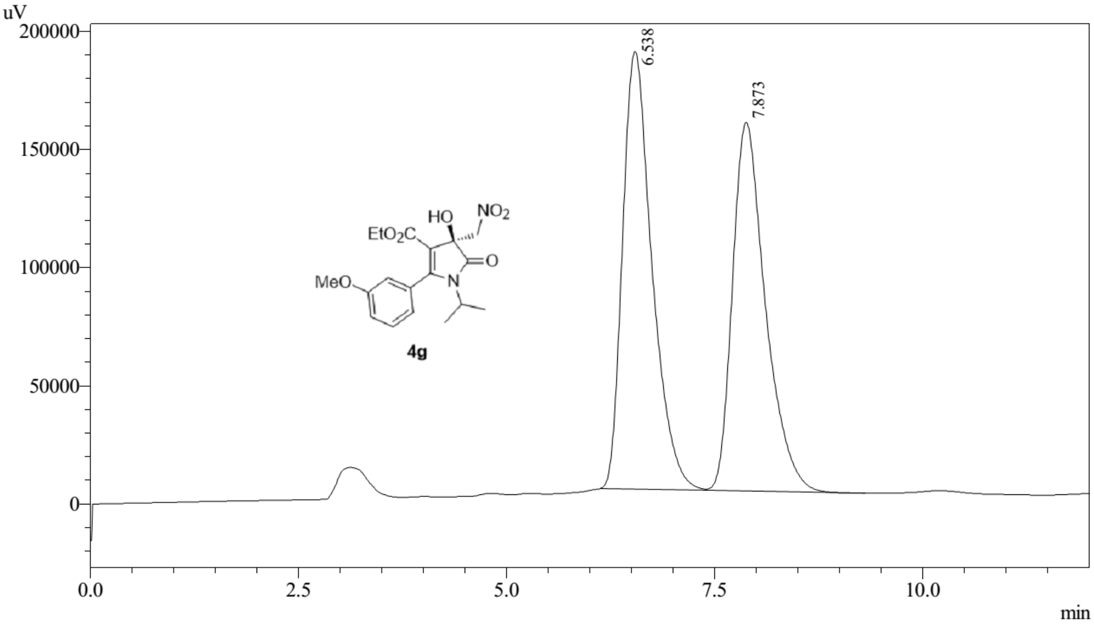

1 Det.A Ch1 / 254nm

Detector A Ch1 254nm

Peak#	Ret. Time	Area	Height	Area %	Height %
1	6.538	4554844	185183	51.450	54.287
2	7.873	4298119	155938	48.550	45.713
Total		8852963	341122	100.000	100.000

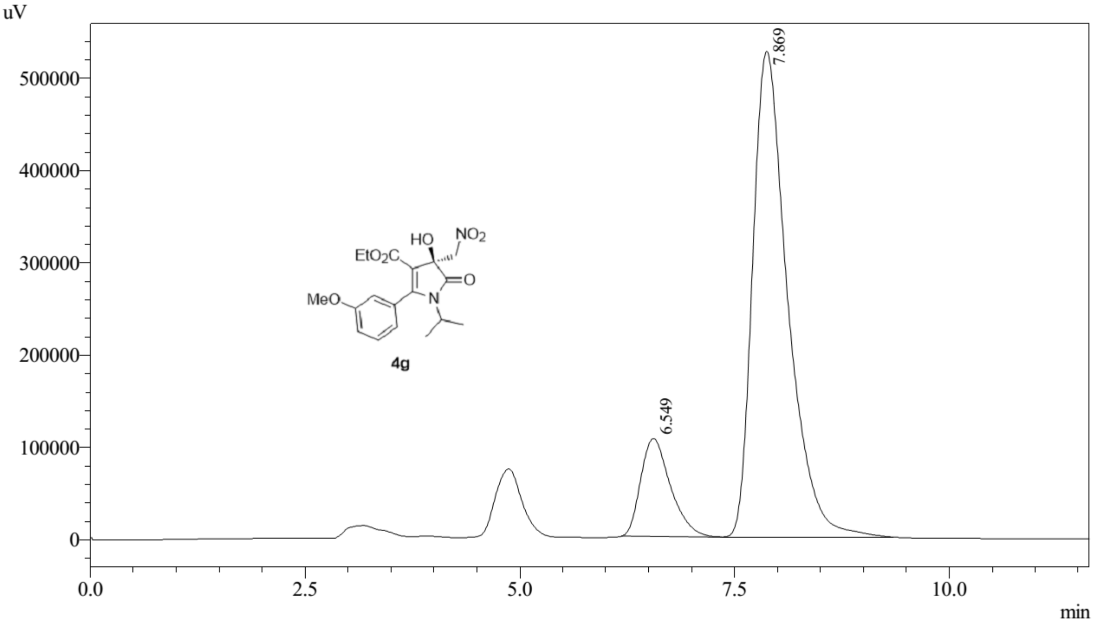

1 Det.A Ch1 / 254nm

Detector A Ch1 254nm

Peak#	Ret. Time	Area	Height	Area %	Height %
1	6.549	2558160	105999	14.672	16.761
2	7.869	14877166	526411	85.328	83.239
Total		17435326	632409	100.000	100.000

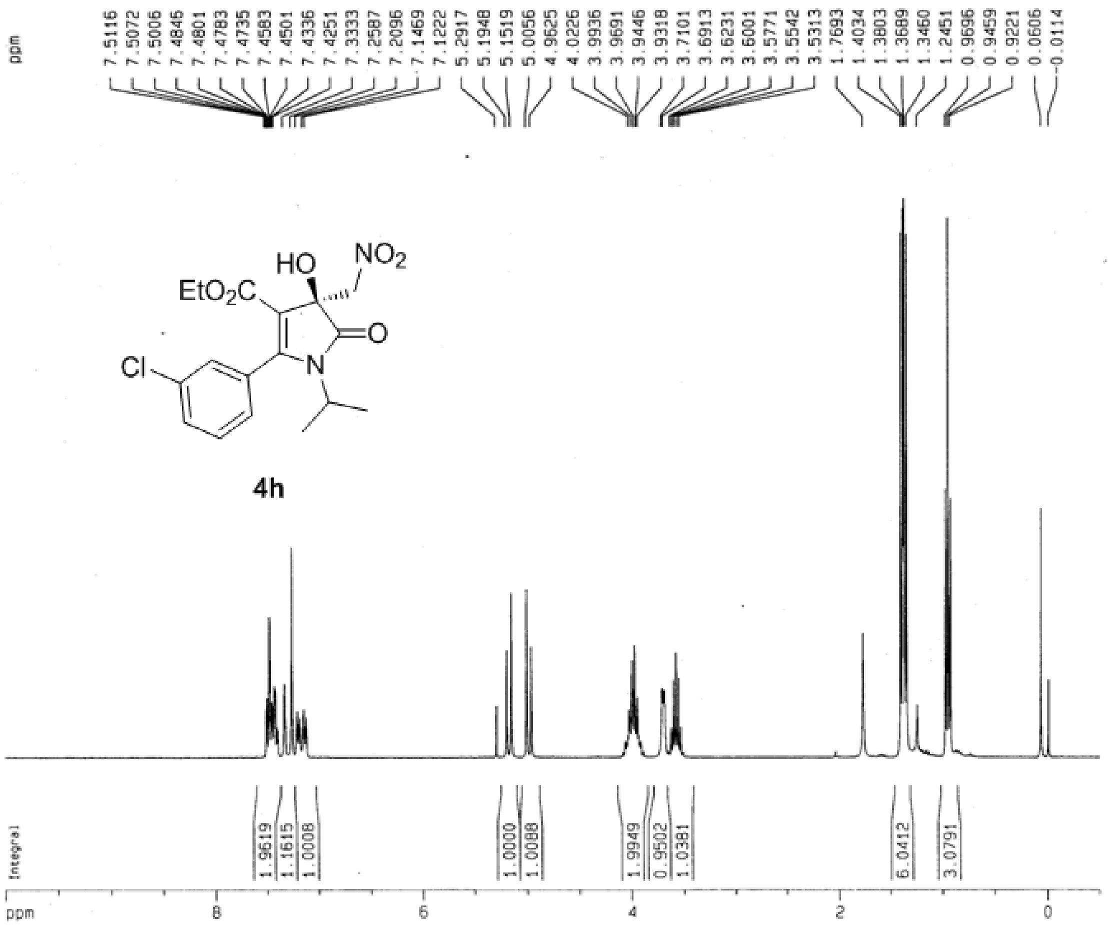
ppm
7.5116 7.5072 7.5006 7.4846 7.4801 7.4783 7.4735 7.4583 7.4501 7.4336 7.4251 7.3333 7.2587 7.2096 7.1469 7.1222 5.2917 5.1948 5.1519 5.0056 4.9625 4.0226 3.9936 3.9691 3.9446 3.9318 3.7101 3.6913 3.6231 3.6001 3.5771 3.5542 3.5313 1.7693 1.4034 1.3803 1.3689 1.3460 1.2451 0.9696 0.9459 0.9221 0.0606 -0.0114
HO
NO_2
EtO_2C
O
Cl
N
4h
Integral
1.9619 1.1615 1.0008 1.0000 1.0088 1.9949 0.9502 1.0381 6.0412 3.0791
ppm
8
6
4
2
0

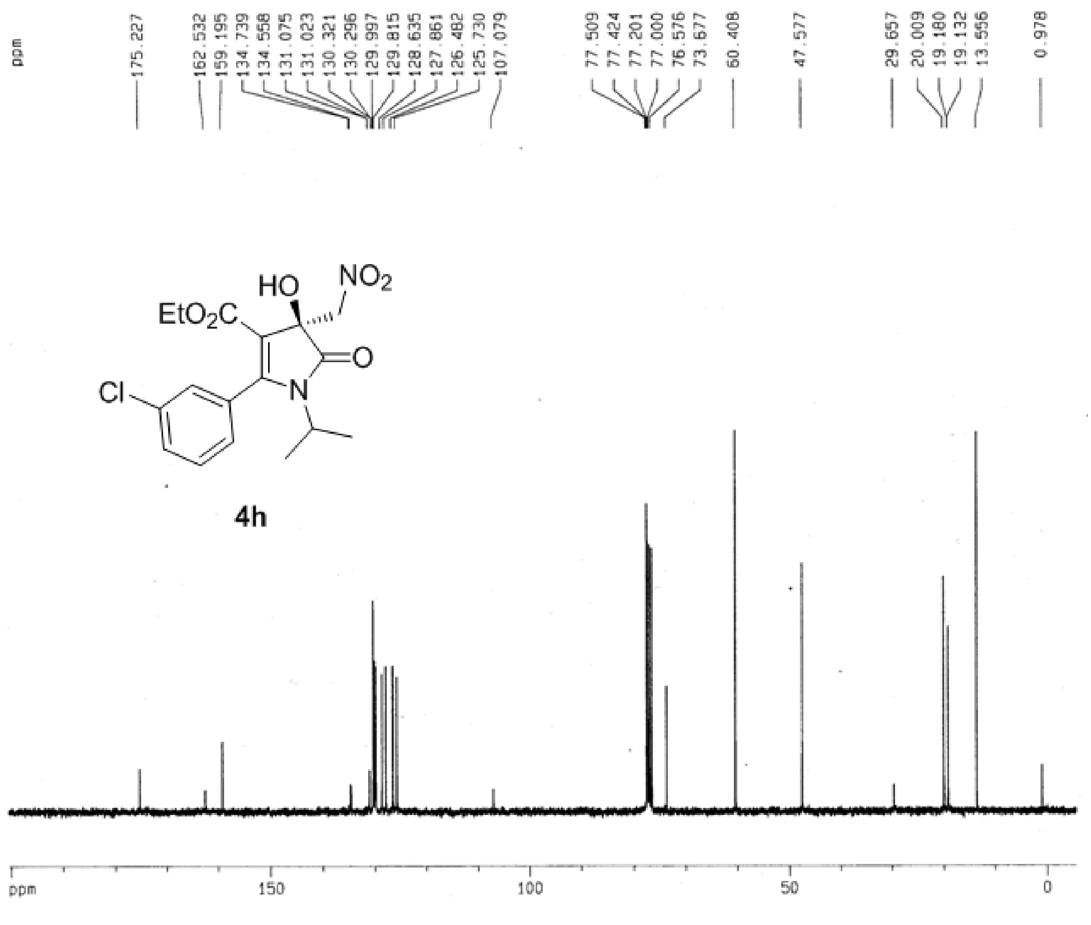
ppm
175.227 162.532 159.195 134.739 134.558 131.075 131.023 130.321 130.296 129.997 129.815 128.635 127.861 126.482 125.730 107.079 77.509 77.424 77.201 77.000 76.576 73.677 60.408 47.577 29.657 20.009 19.180 19.132 13.556 0.978
HO
NO_2
EtO_2C
O
Cl
N
4h
ppm
150
100
50
0

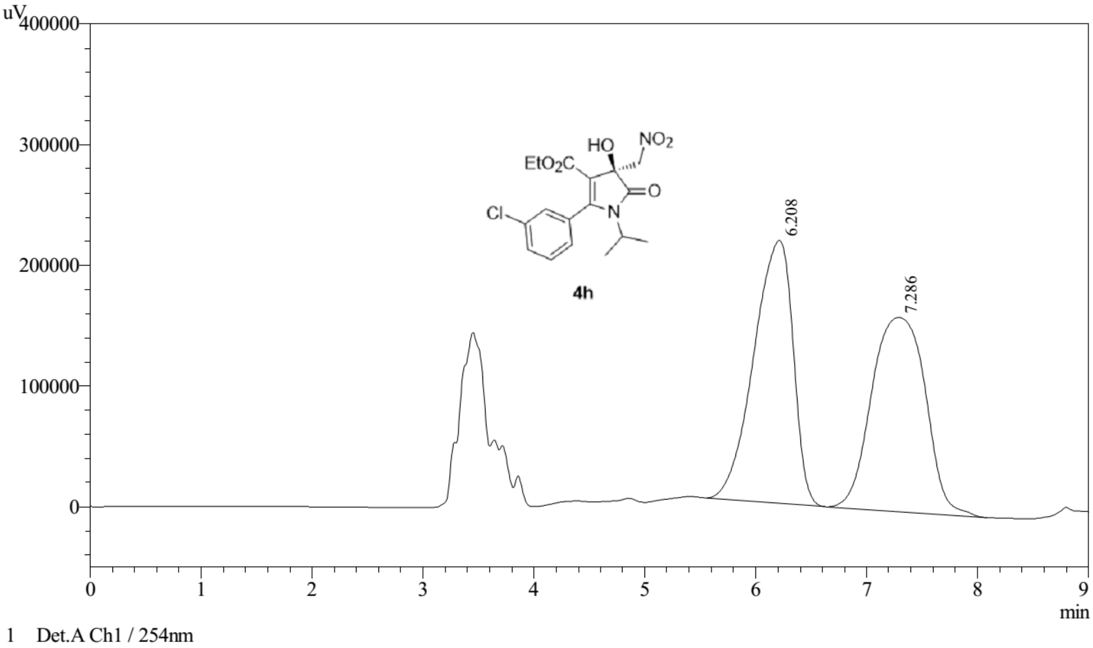

Detector A Ch1 254nm

Peak#	Ret. Time	Area	Height	Area %	Height %
1	6.208	5371811	217766	49.720	57.499
2	7.286	5432274	160962	50.280	42.501
Total		10804085	378728	100.000	100.000

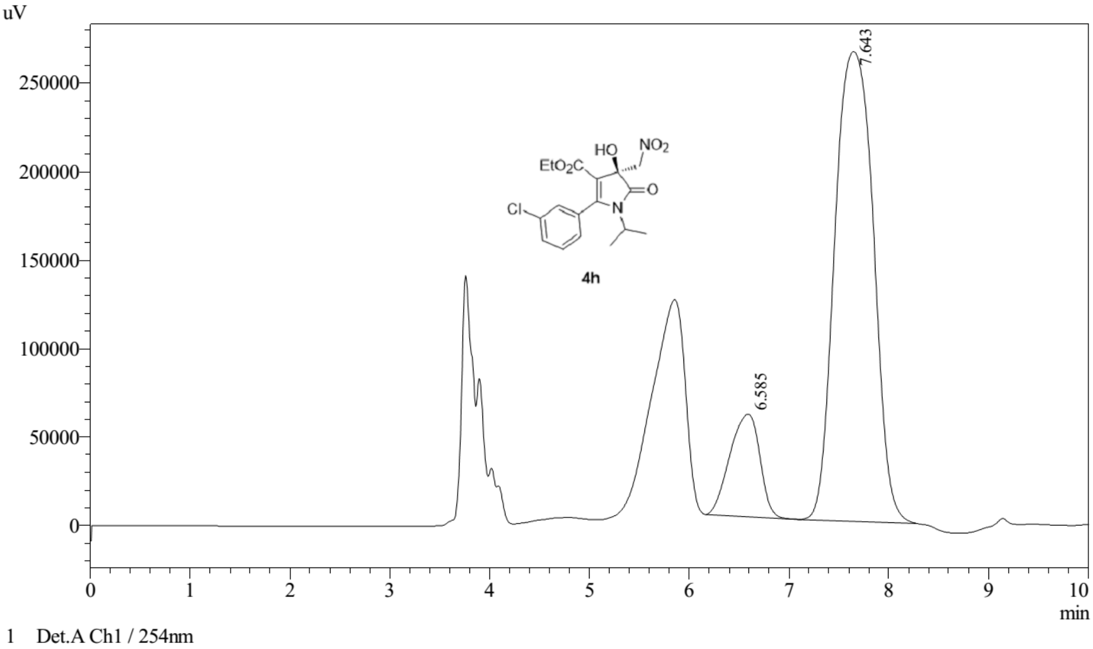

Detector A Ch1 254nm

Peak#	Ret. Time	Area	Height	Area %	Height %
1	6.585	1206559	57942	14.471	17.923
2	7.643	7131150	265347	85.529	82.077
Total		8337709	323288	100.000	100.000

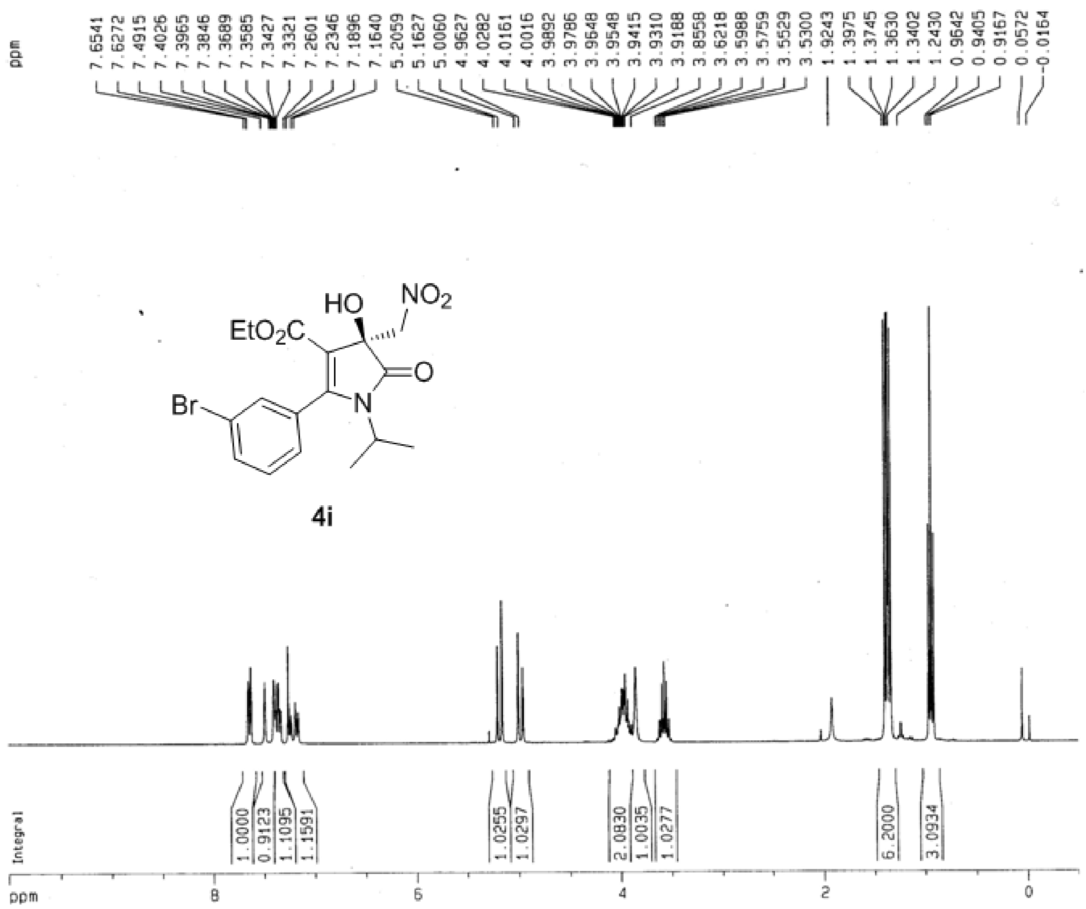
ppm
7.6541 7.6272 7.4915 7.4026 7.3965 7.3846 7.3689 7.3585 7.3427 7.3321 7.2601 7.2346 7.1896 7.1640
5.2059 5.1627 5.0060 4.9627
4.0282 4.0161 4.0016 3.9892 3.9786 3.9648 3.9548 3.9415 3.9310 3.9188 3.8558 3.6218 3.5988 3.5759 3.5529 3.5300
1.9243
1.3975 1.3745 1.3630 1.3402 1.2430
0.9642 0.9405 0.9167
0.0572 -0.0164
HO
NO_2
EtO_2C
O
Br
N
4i
Integral
1.0000 0.9123 1.1095 1.1591
1.0255 1.0297
2.0830 1.0035 1.0277
6.2000
3.0934
ppm
8
6
4
2
0

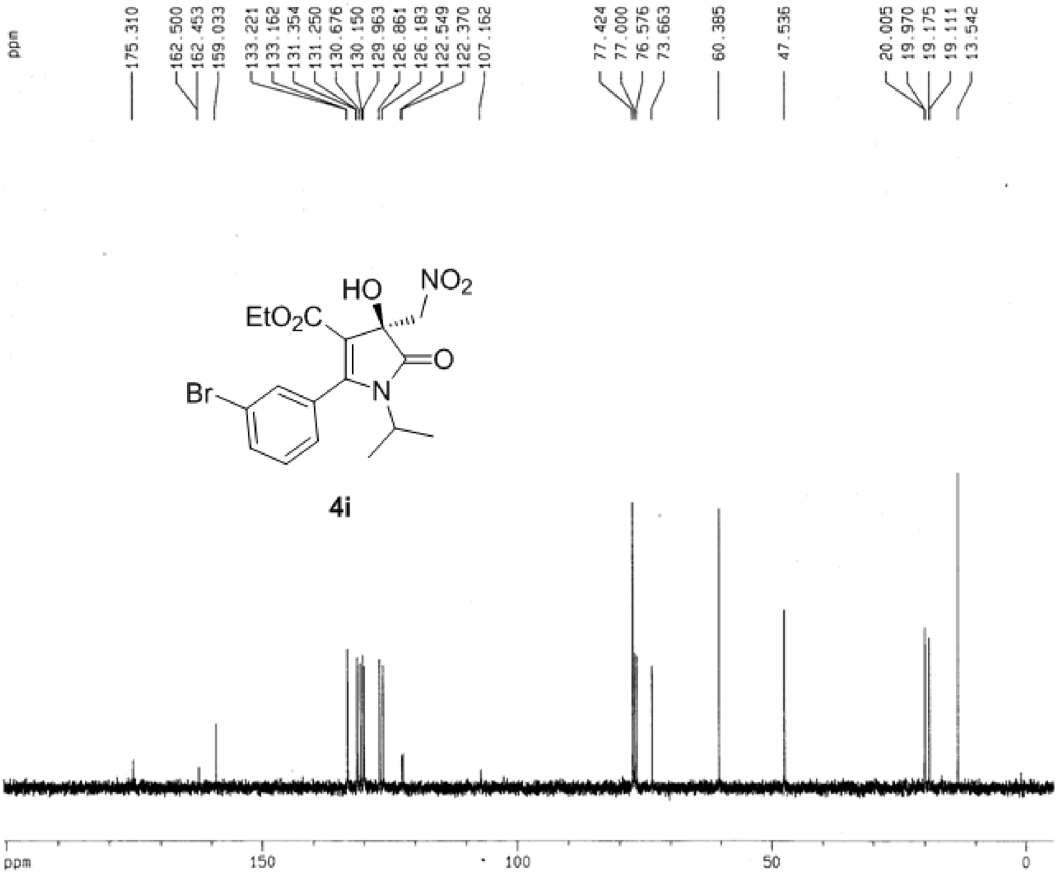
ppm
175.310
162.500 162.453 159.033
133.221 133.162 131.354 131.250 130.676 130.150 129.963 126.861 126.183 122.549 122.370 107.162
77.424 77.000 76.576 73.663
60.385
47.536
20.005 19.970 19.175 19.111 13.542
HO
NO_2
EtO_2C
O
Br
N
4i
ppm
150
100
50
0

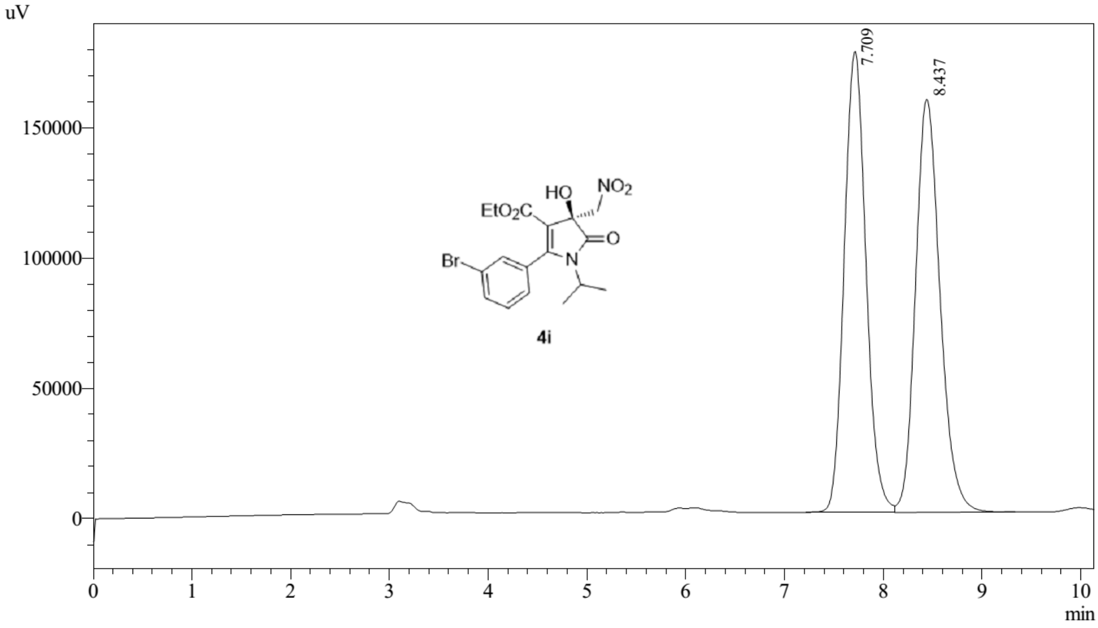

1 Det.A Ch1 / 254nm

Detector A Ch1 254nm

Peak#	Ret. Time	Area	Height	Area %	Height %
1	7.709	2709797	176841	49.728	52.725
2	8.437	2739413	158559	50.272	47.275
Total		5449210	335399	100.000	100.000

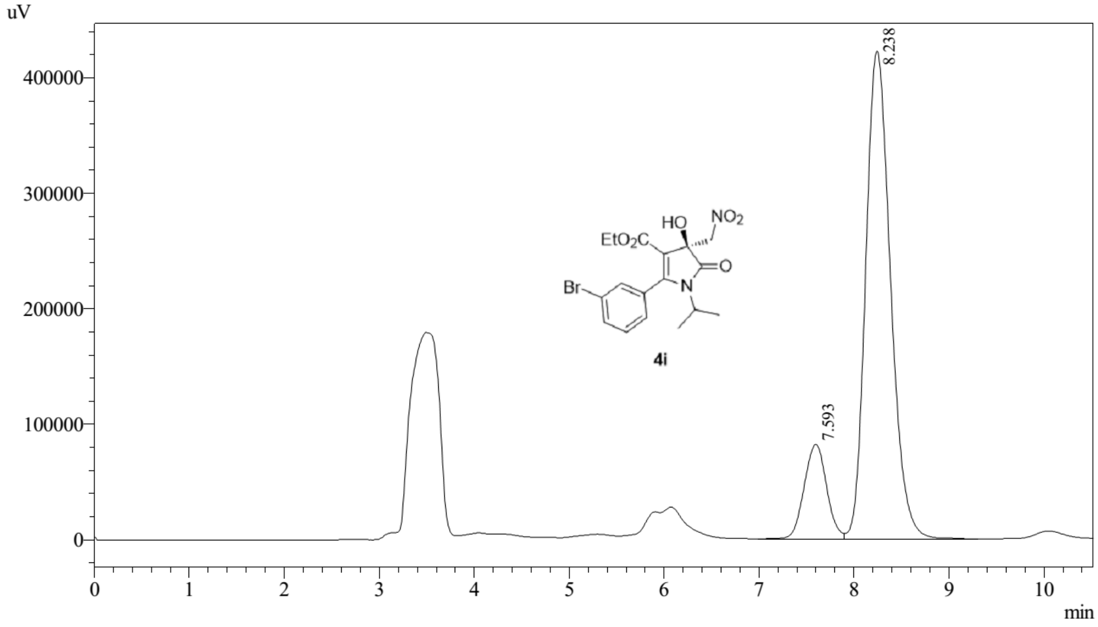

1 Det.A Ch1 / 254nm

Detector A Ch1 254nm

Peak#	Ret. Time	Area	Height	Area %	Height %
1	7.593	1352217	81845	14.994	16.228
2	8.238	7665886	422488	85.006	83.772
Total		9018103	504333	100.000	100.000

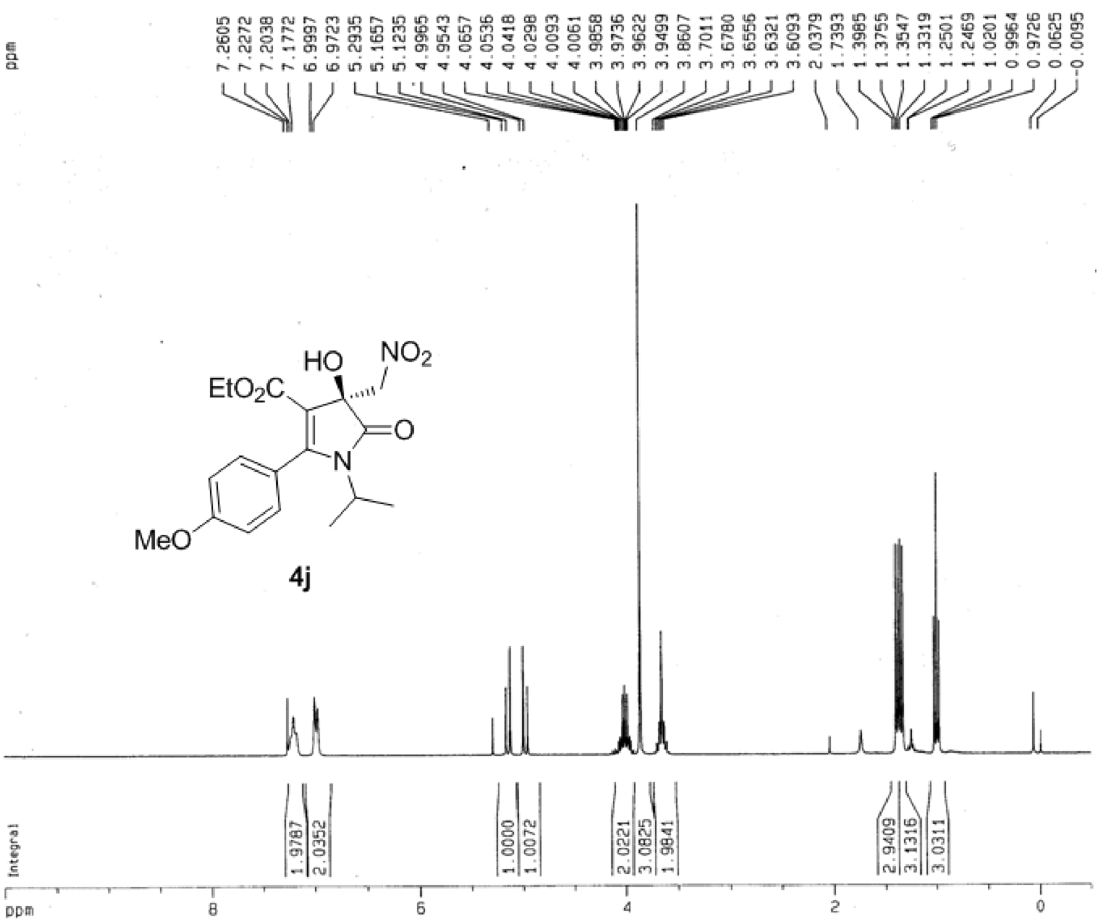
ppm
7.2605 7.2272 7.2038 7.1772 6.9997 6.9723 5.2935 5.1657 5.1235 4.9965 4.9543 4.0657 4.0536 4.0418 4.0298 4.0093 4.0061 3.9858 3.9736 3.9622 3.9499 3.8607 3.7011 3.6780 3.6556 3.6321 3.6093 2.0379 1.7393 1.3985 1.3755 1.3547 1.3319 1.2501 1.2469 1.0201 0.9964 0.9726 0.0625 -0.0095
HO
NO2
EtO2C
O
N
MeO
4j
Integral
1.9787 2.0352 1.0000 1.0072 2.0221 3.0825 1.9841 2.9409 3.1316 3.0311
ppm
8
6
4
2
0

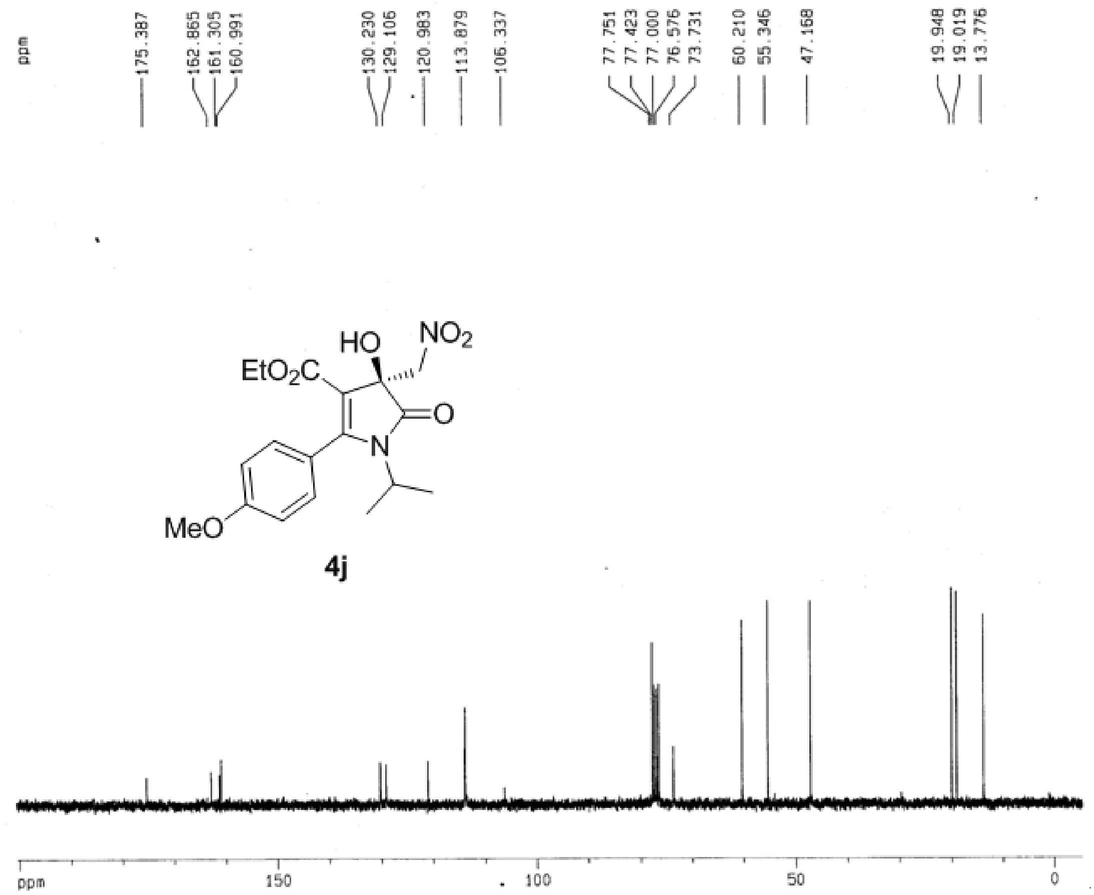
ppm
175.387 162.865 161.305 160.991 130.230 129.106 120.983 113.879 106.337 77.751 77.423 77.000 76.576 73.731 60.210 55.346 47.168 19.948 19.019 13.776
HO
NO2
EtO2C
O
N
MeO
4j
ppm
150
100
50
0

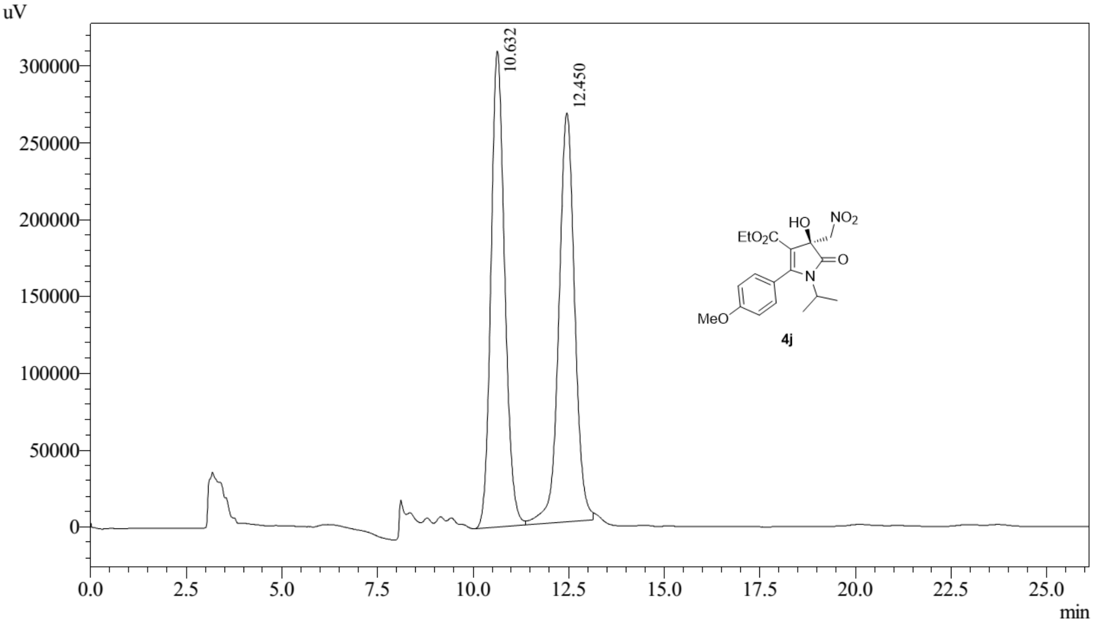

1 Det.A Ch1 / 254nm

Detector A Ch1 254nm

Peak#	Ret. Time	Area	Height	Area %	Height %
1	10.632	7754028	310076	50.194	53.785
2	12.450	7694145	266436	49.806	46.215
Total		15448173	576511	100.000	100.000

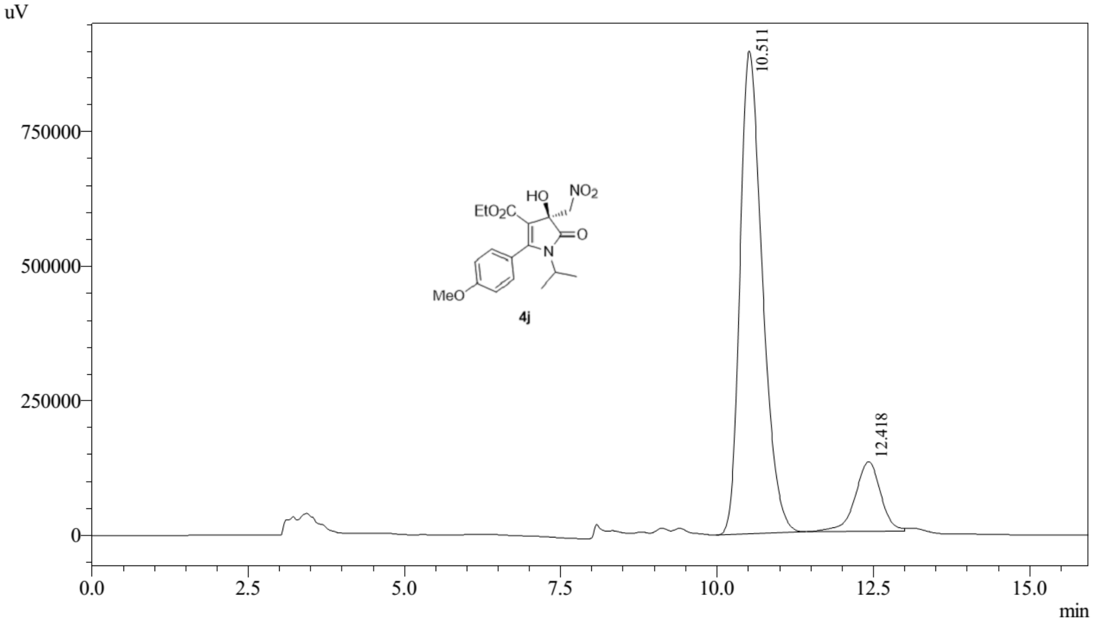

1 Det.A Ch1 / 254nm

Detector A Ch1 254nm

Peak#	Ret. Time	Area	Height	Area %	Height %
1	10.511	22399812	897656	85.775	87.356
2	12.418	3714925	129932	14.225	12.644
Total		26114737	1027588	100.000	100.000

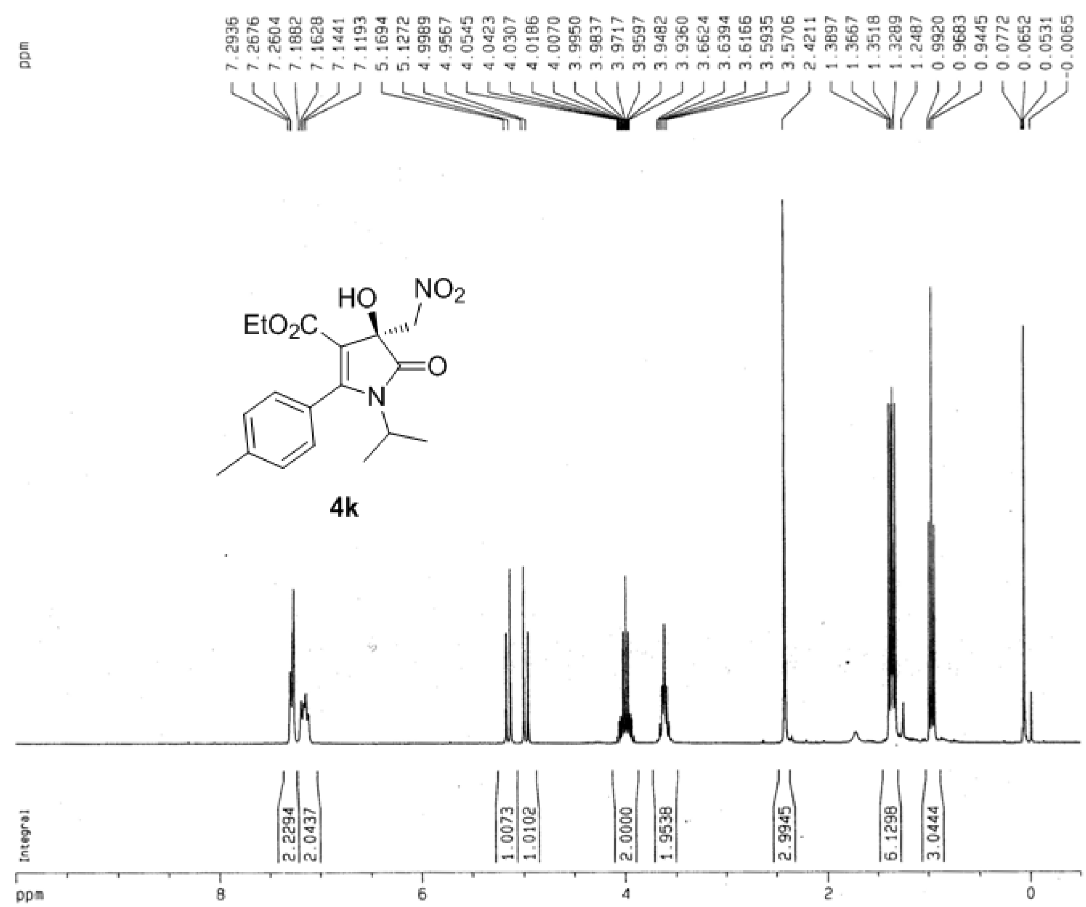
ppm
7.2936 7.2676 7.2604 7.1882 7.1628 7.1441 7.1193 5.1694 5.1272 4.9989 4.9567 4.0545 4.0423 4.0307 4.0186 4.0070 3.9950 3.9837 3.9717 3.9597 3.9482 3.9360 3.6624 3.6394 3.6166 3.5935 3.5706 2.4211 1.3897 1.3667 1.3518 1.3289 1.2487 0.9920 0.9683 0.9445 0.0772 0.0652 0.0531 -0.0065
EtO$_2$C
HO
NO$_2$
O
N
4k
Integral
2.2294 2.0437 1.0073 1.0102 2.0000 1.9538 2.9945 6.1298 3.0444
ppm
8
6
4
2
0

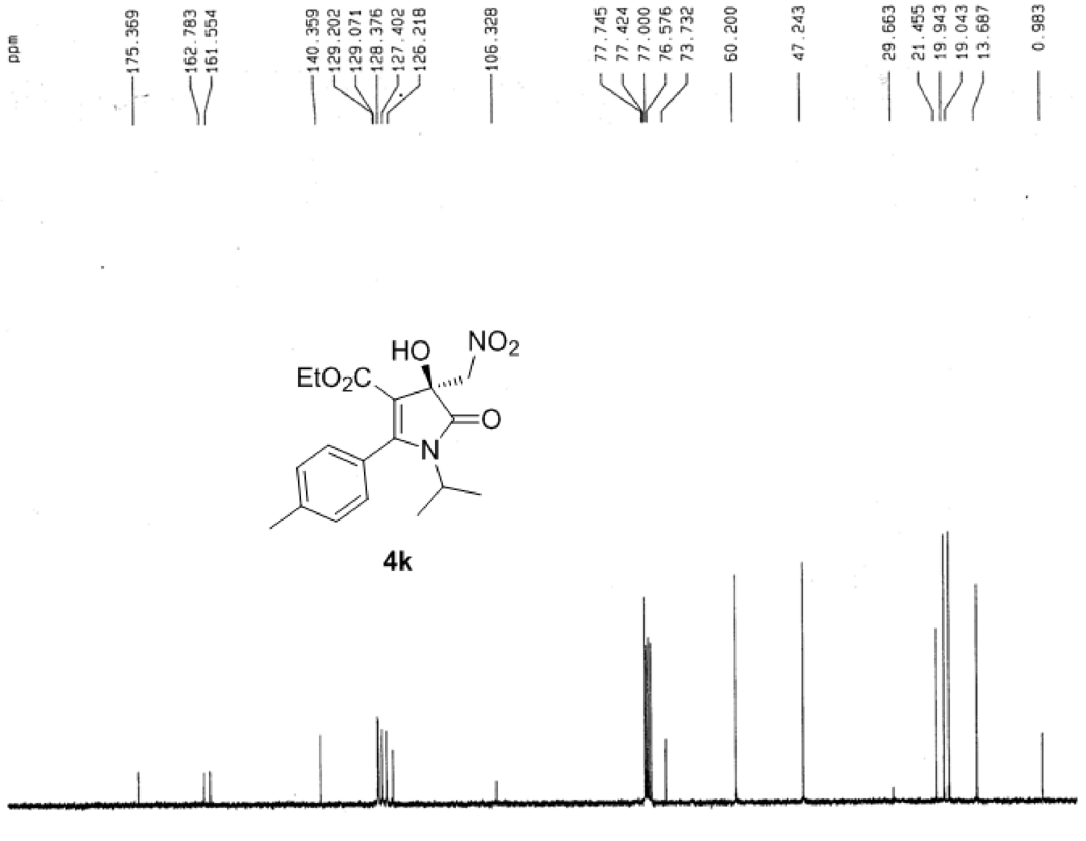
ppm
175.369 162.783 161.554 140.359 129.202 129.071 128.376 127.402 126.218 106.328 77.745 77.424 77.000 76.576 73.732 60.200 47.243 29.663 21.455 19.943 19.043 13.687 0.983
EtO$_2$C
HO
NO$_2$
O
N
4k
ppm
150
100
50
0

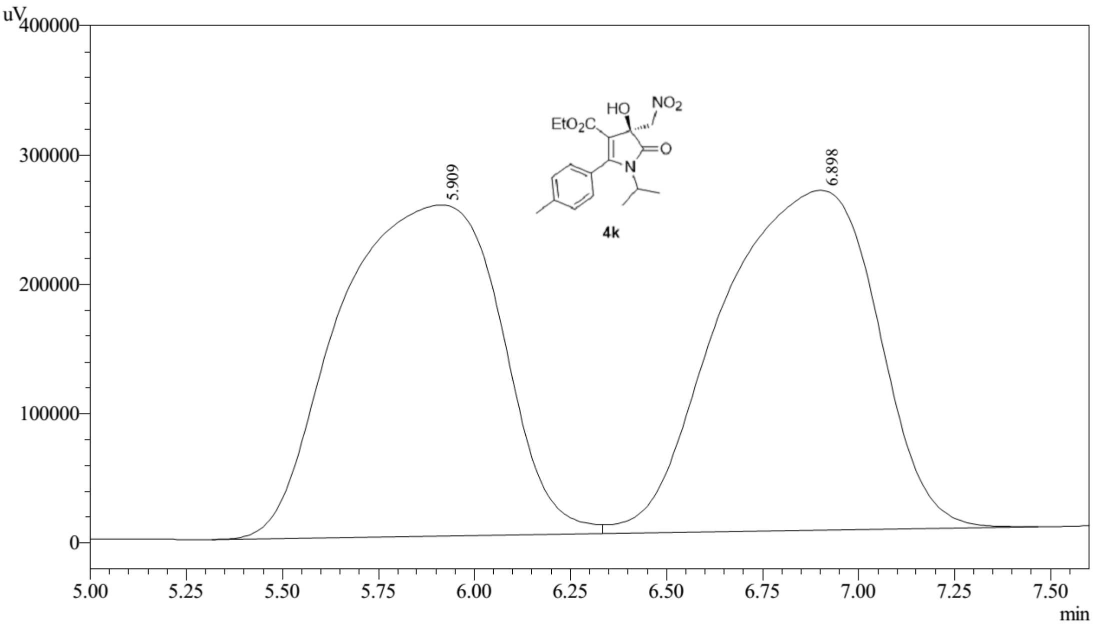

1 Det.A Ch1 / 254nm

Detector A Ch1 254nm

Peak#	Ret. Time	Area	Height	Area %	Height %
1	5.909	7536771	255969	50.169	49.344
2	6.898	7486076	262778	49.831	50.656
Total		15022846	518747	100.000	100.000

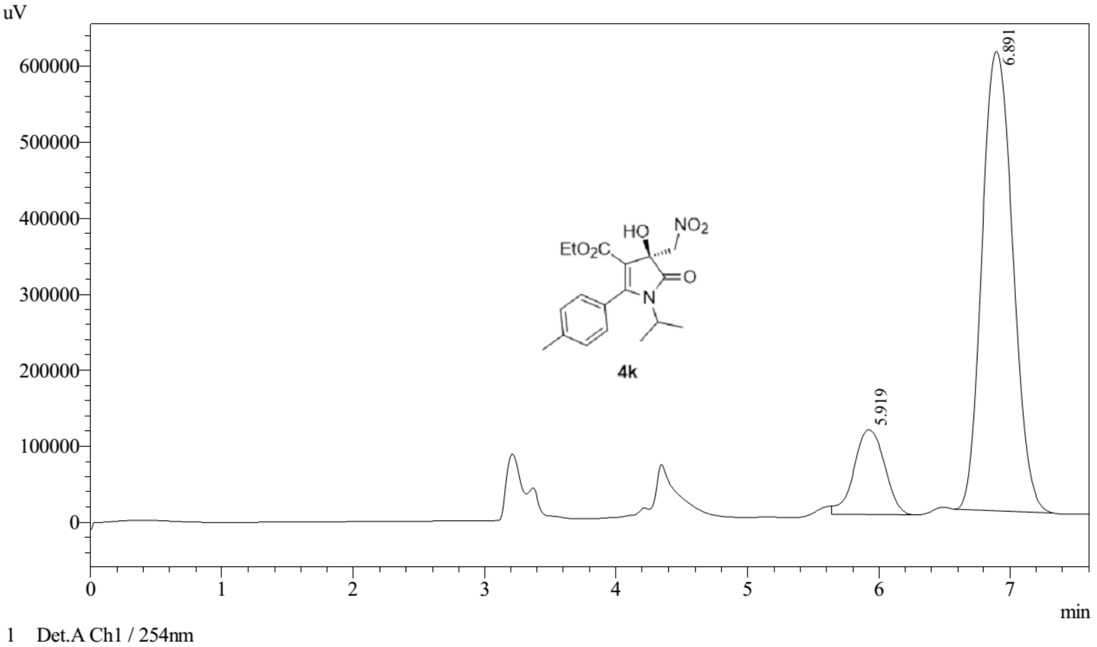

1 Det.A Ch1 / 254nm

Detector A Ch1 254nm

Peak#	Ret. Time	Area	Height	Area %	Height %
1	5.919	1808477	111332	15.474	15.557
2	6.891	9878533	604292	84.526	84.443
Total		11687010	715624	100.000	100.000

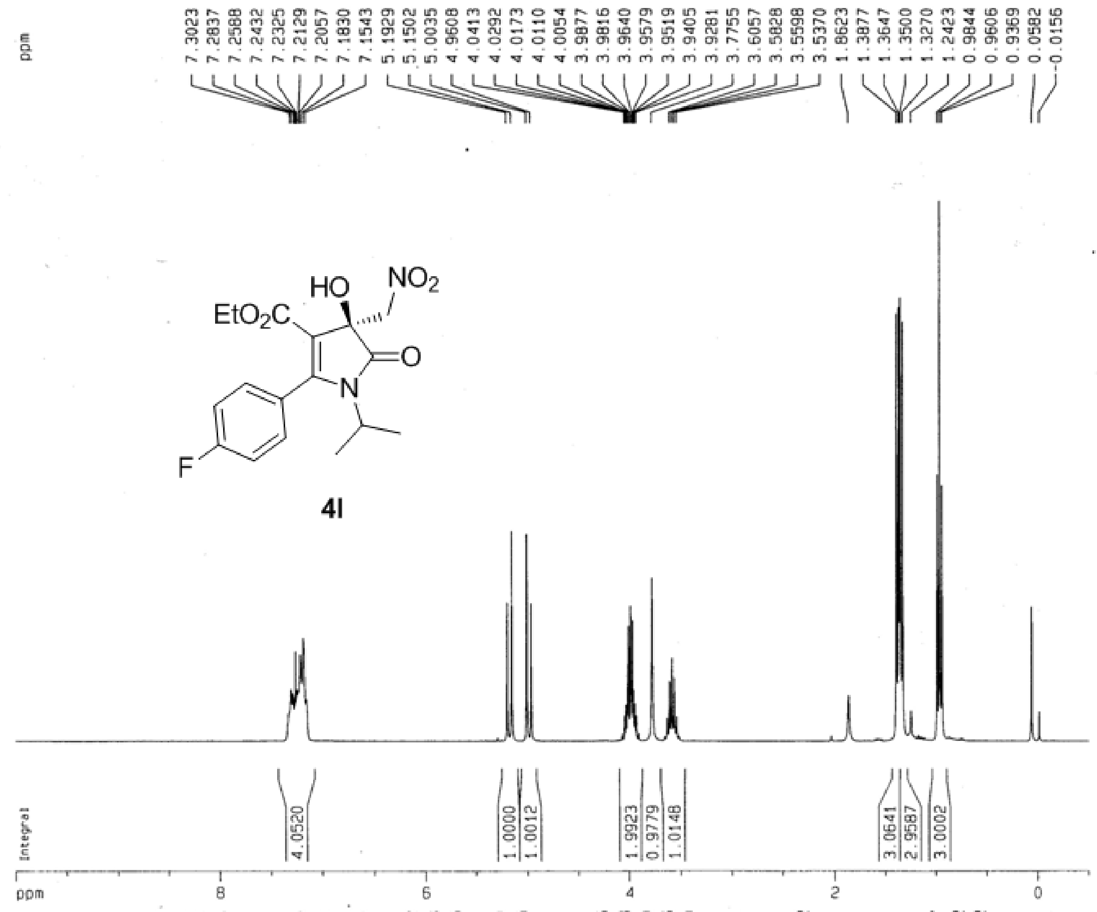
ppm
7.3023 7.2837 7.2588 7.2432 7.2325 7.2129 7.2057 7.1830 7.1543 5.1929 5.1502 5.0035 4.9608 4.0413 4.0292 4.0173 4.0110 4.0054 3.9877 3.9816 3.9640 3.9579 3.9519 3.9405 3.9281 3.7755 3.6057 3.5828 3.5598 3.5370 1.8623 1.3877 1.3647 1.3500 1.3270 1.2423 0.9844 0.9606 0.9369 0.0582 -0.0156
HO
NO2
EtO2C
O
N
F
4l
Integral
4.0520
1.0000
1.0012
1.9923
0.9779
1.0148
3.0641
2.9587
3.0002
ppm
8
6
4
2
0

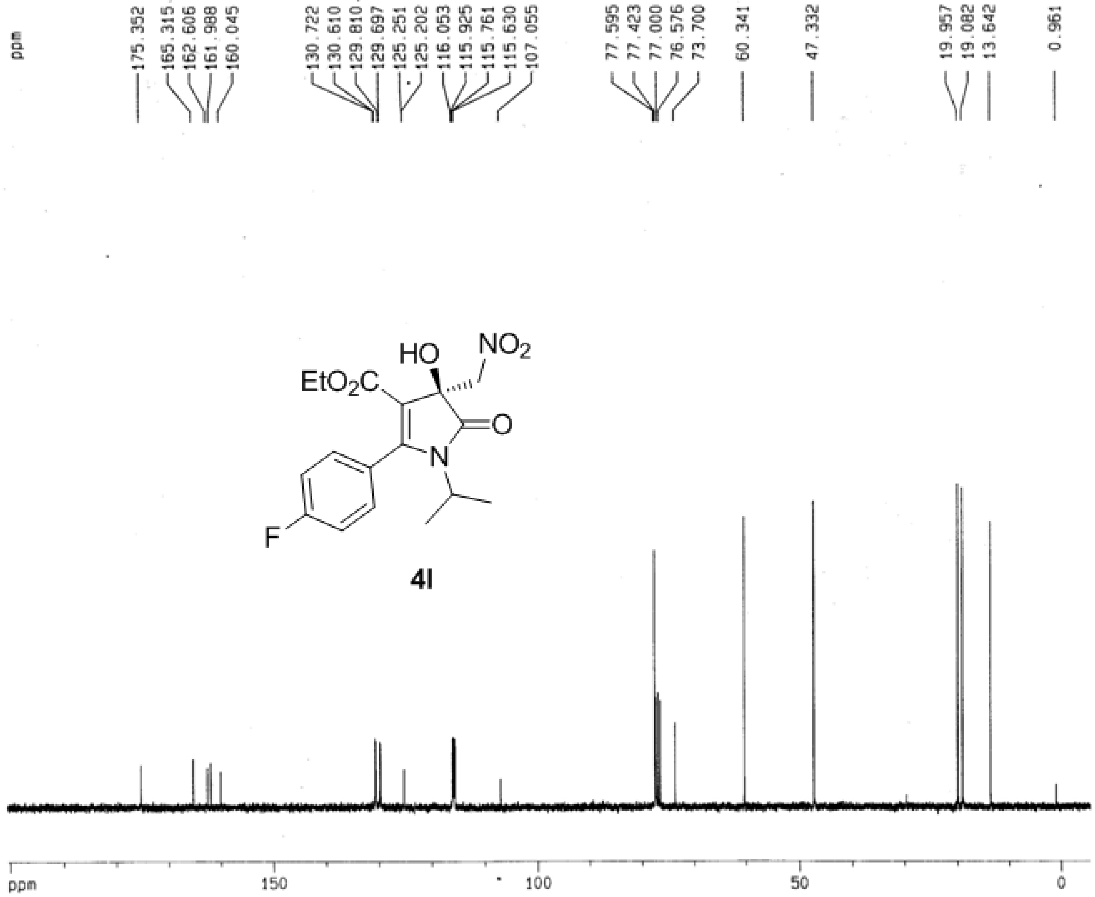
ppm
175.352 165.315 162.606 161.988 160.045 130.722 130.610 129.810 129.697 125.251 125.202 116.053 115.925 115.761 115.630 107.055 77.595 77.423 77.000 76.576 73.700 60.341 47.332 19.957 19.082 13.642 0.961
HO
NO2
EtO2C
O
N
F
4l
ppm
150
100
50
0

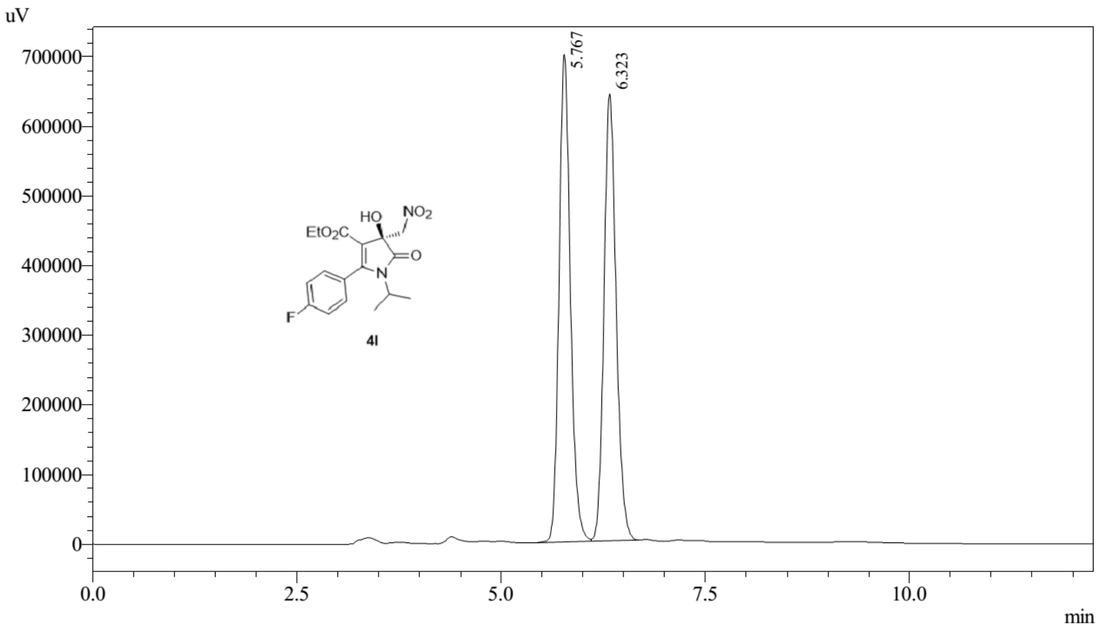

1 Det.A Ch1 / 254nm

Detector A Ch1 254nm

Peak#	Ret. Time	Area	Height	Area %	Height %
1	5.767	6796590	700281	50.453	52.174
2	6.323	6674490	641919	49.547	47.826
Total		13471080	1342200	100.000	100.000

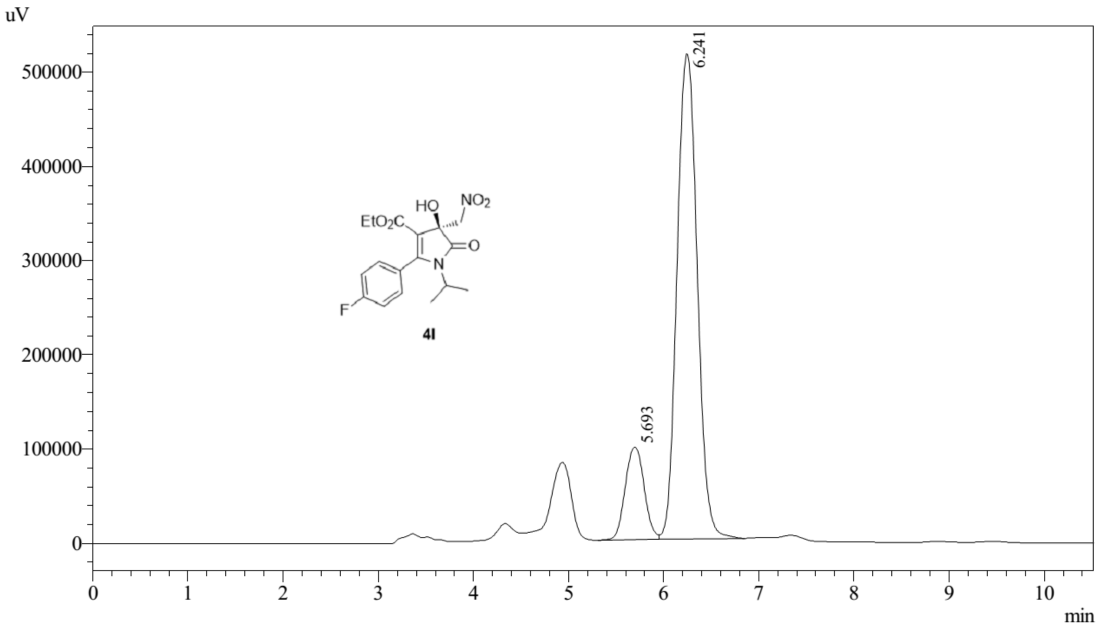

1 Det.A Ch1 / 254nm

Detector A Ch1 254nm

Peak#	Ret. Time	Area	Height	Area %	Height %
1	5.693	1367967	98399	15.242	16.038
2	6.241	7606727	515125	84.758	83.962
Total		8974694	613524	100.000	100.000

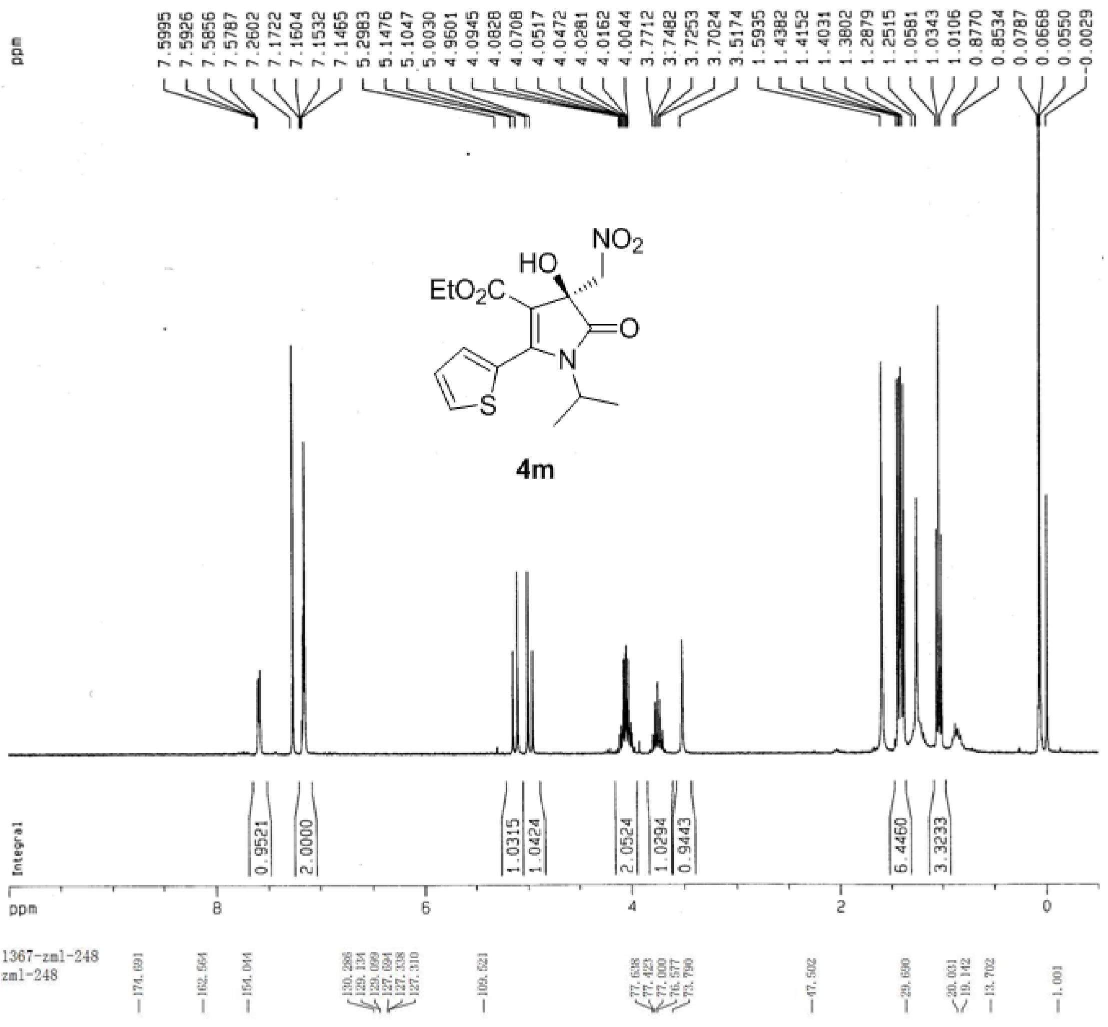

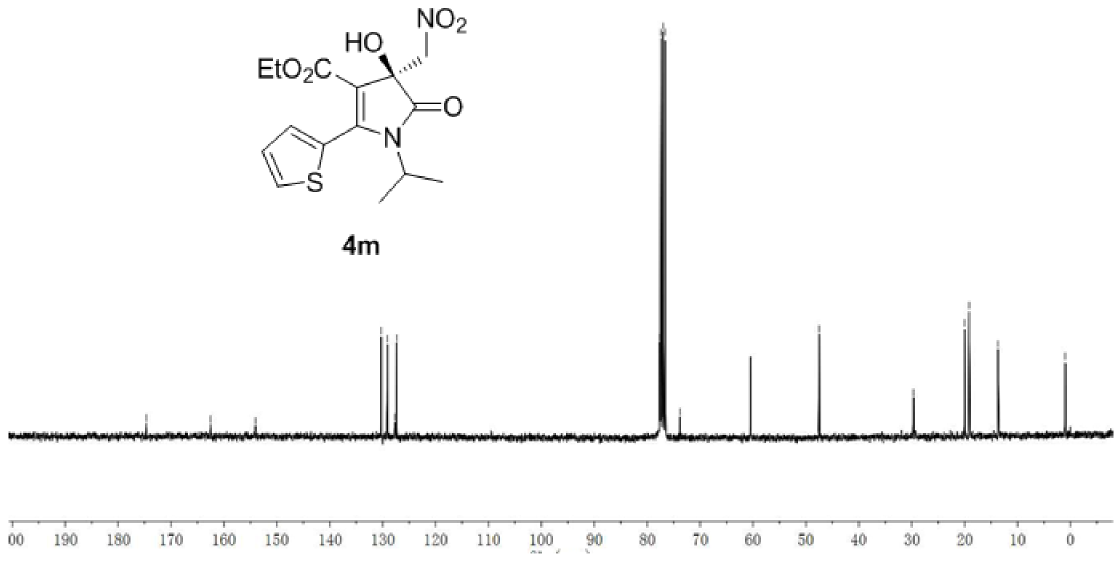

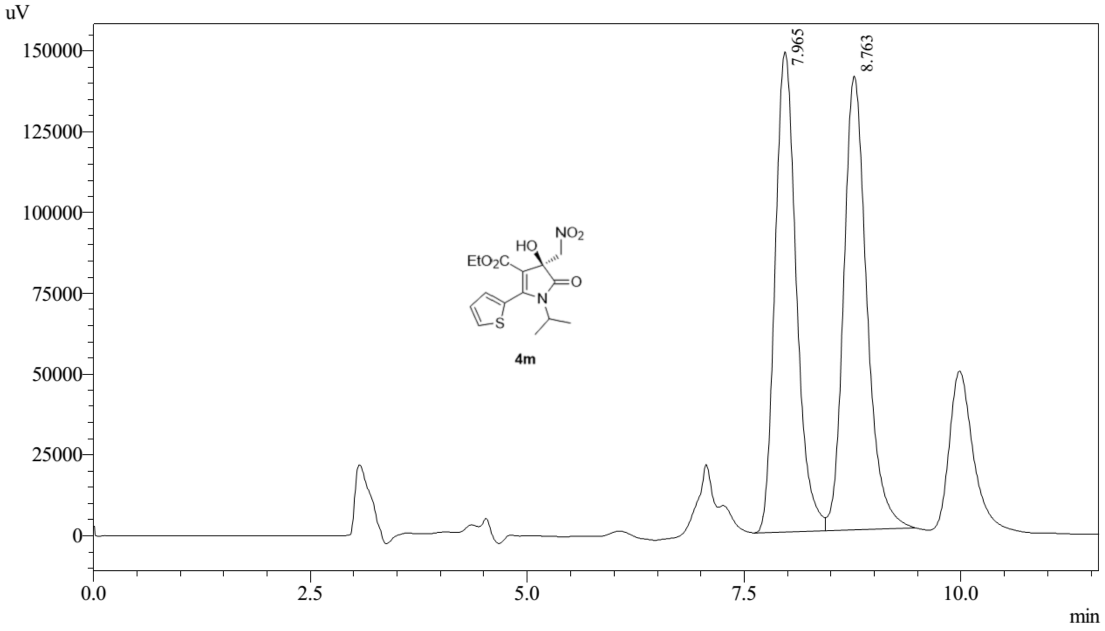

1 Det.A Ch1 / 254nm

Detector A Ch1 254nm

Peak#	Ret. Time	Area	Height	Area %	Height %
1	7.965	2533357	148632	49.230	51.413
2	8.763	2612614	140462	50.770	48.587
Total		5145971	289094	100.000	100.000

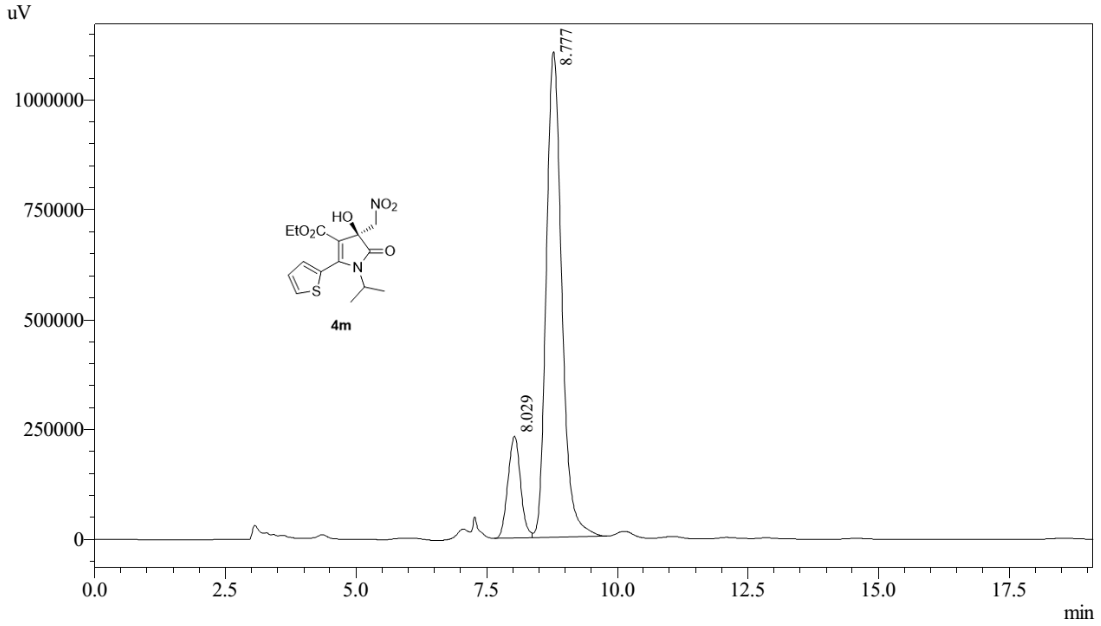

1 Det.A Ch1 / 254nm

Detector A Ch1 254nm

Peak#	Ret. Time	Area	Height	Area %	Height %
1	8.029	3921129	231770	14.682	17.338
2	8.777	22785352	1105014	85.318	82.662
Total		26706480	1336784	100.000	100.000

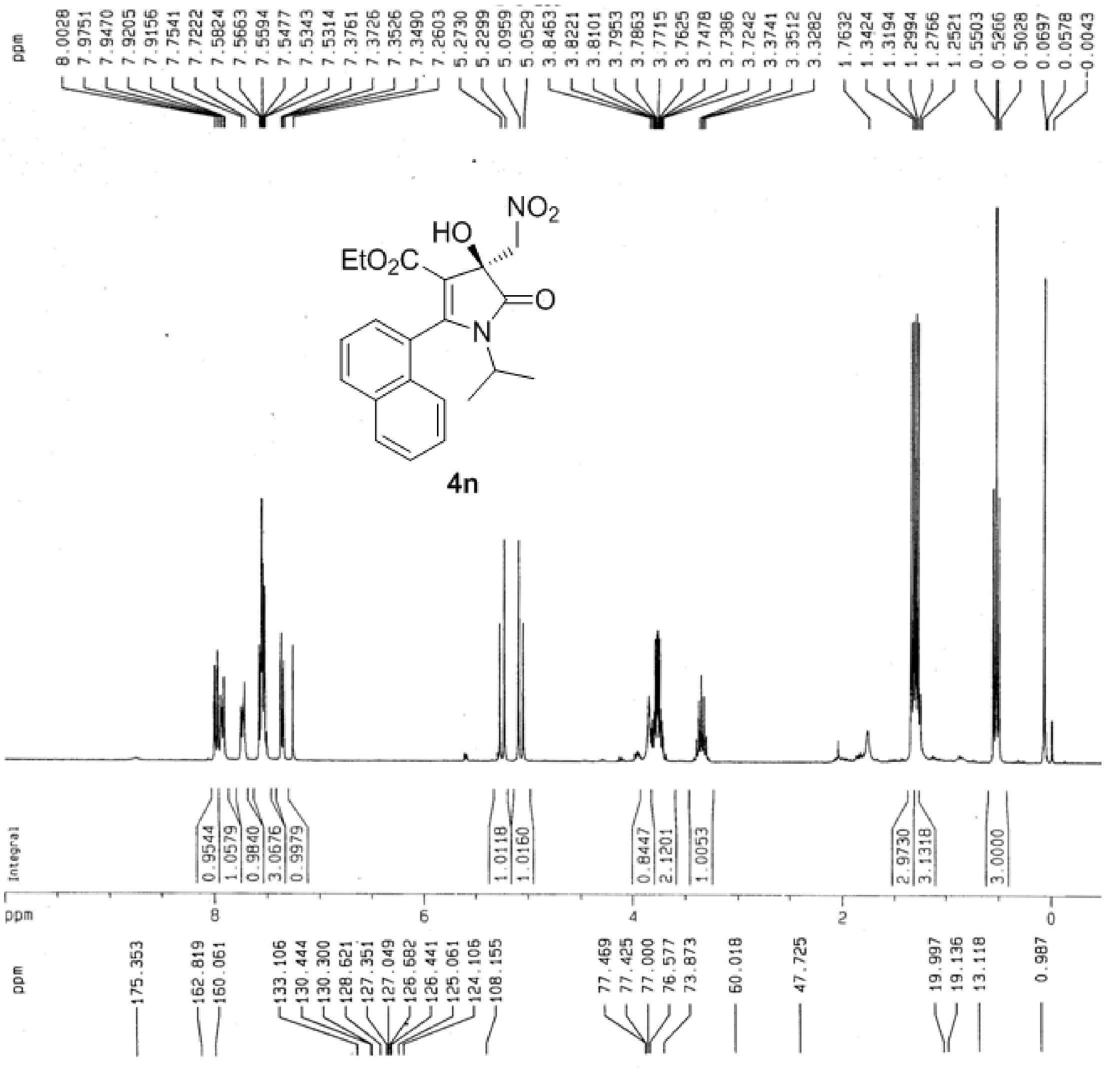
ppm
8.0028 7.9751 7.9470 7.9205 7.9156 7.7541 7.7222 7.5824 7.5663 7.5594 7.5477 7.5343 7.5314 7.3761 7.3726 7.3526 7.3490 7.2603 5.2730 5.2299 5.0959 5.0529 3.8463 3.8221 3.8101 3.7953 3.7863 3.7715 3.7625 3.7478 3.7386 3.7242 3.3741 3.3512 3.3282 1.7632 1.3424 1.3194 1.2994 1.2766 1.2521 0.5503 0.5266 0.5028 0.0697 0.0578 -0.0043
NO2
HO
EtO2C
O
N
4n
Integral
0.9544 1.0579 0.9840 3.0676 0.9979 1.0118 1.0160 0.8447 2.1201 1.0053 2.9730 3.1318 3.0000
ppm
8
6
4
2
0

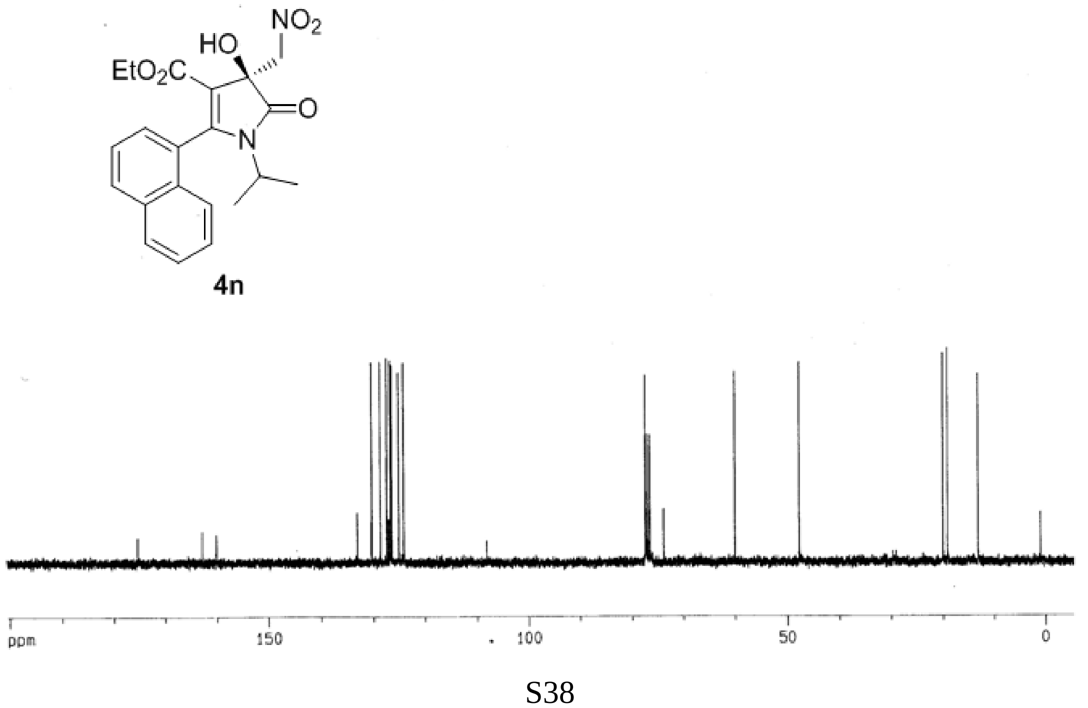
ppm
175.353 162.819 160.061 133.106 130.444 130.300 128.621 127.351 127.049 126.682 126.441 125.061 124.105 108.155 77.469 77.425 77.000 76.577 73.873 60.018 47.725 19.997 19.136 13.118 0.987
NO2
HO
EtO2C
O
N
4n
ppm
150
100
50
0

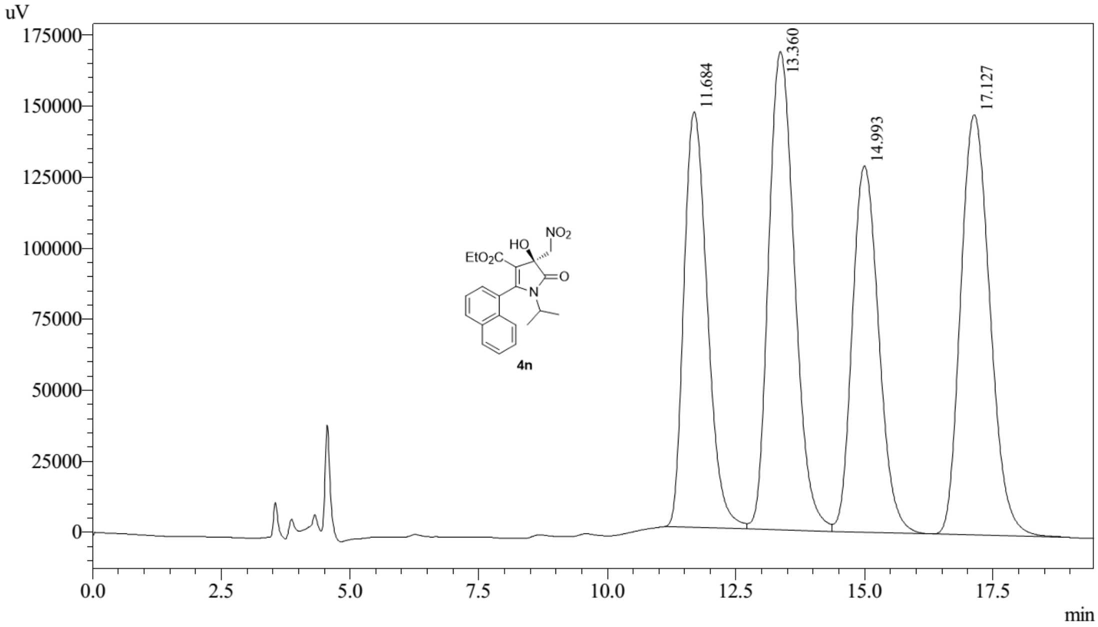

1 Det.A Ch1 / 254nm

Detector A Ch1 254nm

Peak#	Ret. Time	Area	Height	Area %	Height %
1	11.684	4639600	146291	22.025	24.735
2	13.360	5941227	168315	28.204	28.459
3	14.993	4628557	128940	21.972	21.801
4	17.127	5856135	147890	27.800	25.005
Total		21065518	591435	100.000	100.000

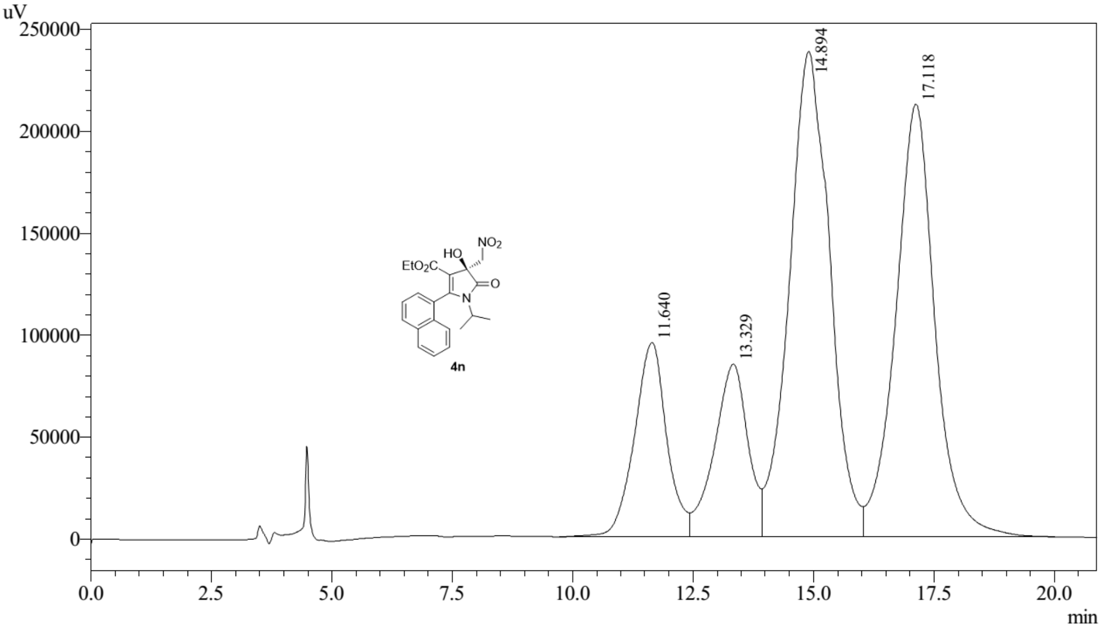

1 Det.A Ch1 / 254nm

Detector A Ch1 254nm

Peak#	Ret. Time	Area	Height	Area %	Height %
1	11.640	4484018	95360	12.930	15.121
2	13.329	4128815	84797	11.906	13.446
3	14.894	13942374	238210	40.203	37.771
4	17.118	12124412	212296	34.961	33.662
Total		34679618	630662	100.000	100.000

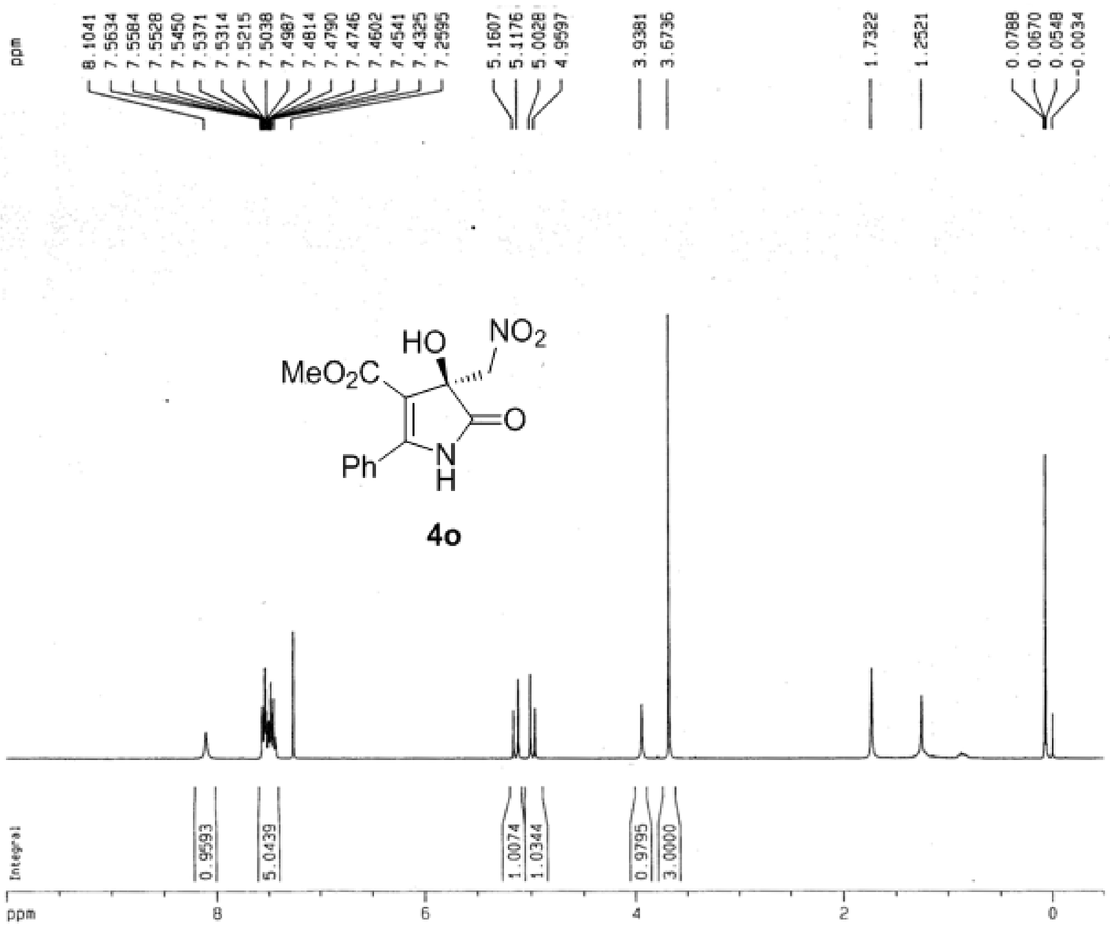
ppm
8.1041
7.5634
7.5584
7.5528
7.5450
7.5371
7.5314
7.5215
7.5038
7.4987
7.4814
7.4790
7.4746
7.4602
7.4541
7.4325
7.2595
5.1607
5.1176
5.0028
4.9597
3.9381
3.6736
1.7322
1.2521
0.0788
0.0670
0.0548
-0.0034
HO
NO2
MeO2C
O
Ph
N
H
4o
Integral
0.9593
5.0439
1.0074
1.0344
0.9795
3.0000
ppm
8
6
4
2
0

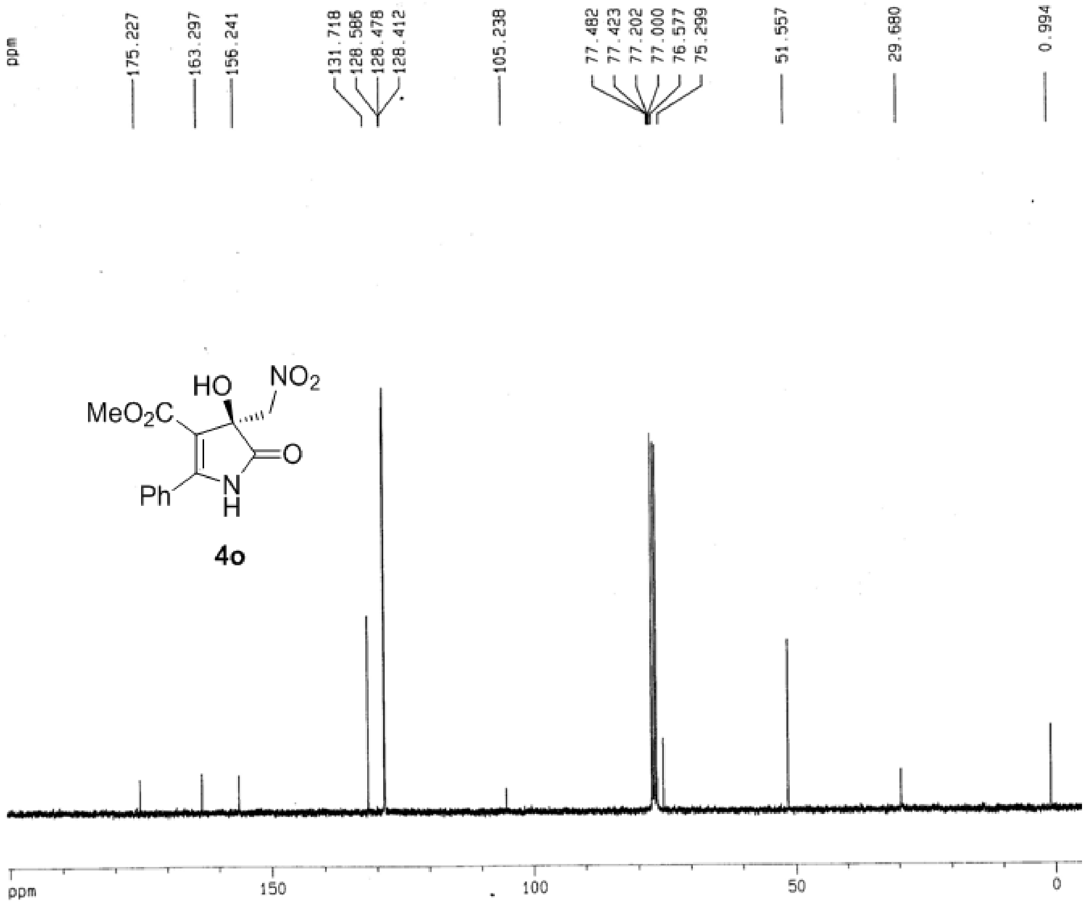
ppm
175.227
163.297
156.241
131.718
128.586
128.478
128.412
105.238
77.482
77.423
77.202
77.000
76.577
75.299
51.557
29.680
0.994
HO
NO2
MeO2C
O
Ph
N
H
4o
ppm
150
100
50
0

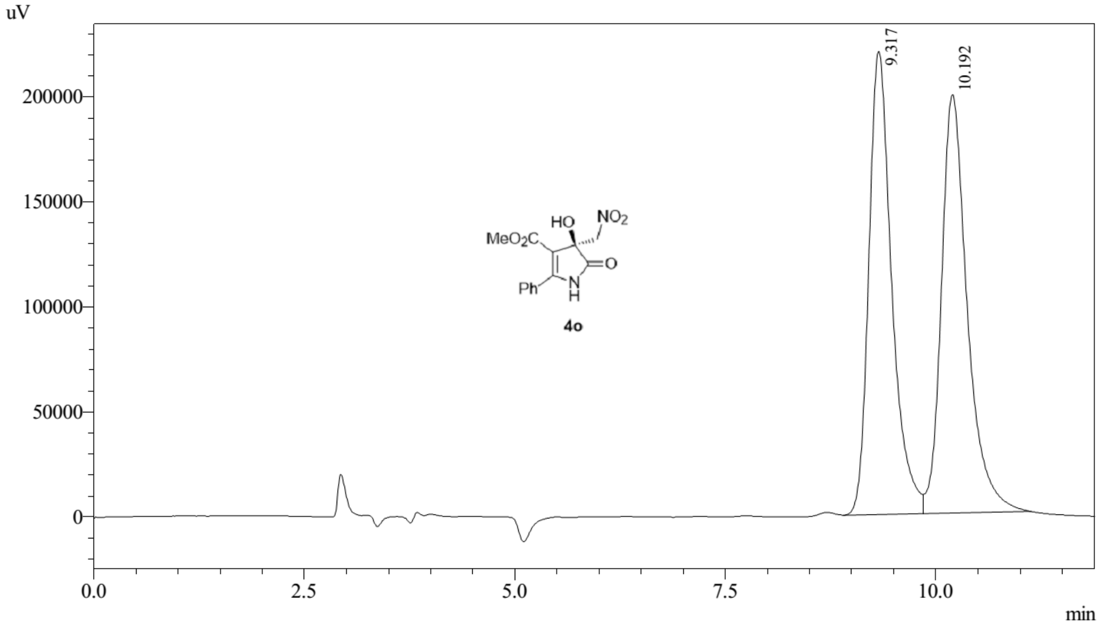

1 Det.A Ch1 / 254nm

Detector A Ch1 254nm

Peak#	Ret. Time	Area	Height	Area %	Height %
1	9.317	4168487	220420	49.448	52.539
2	10.192	4261578	199119	50.552	47.461
Total		8430065	419538	100.000	100.000

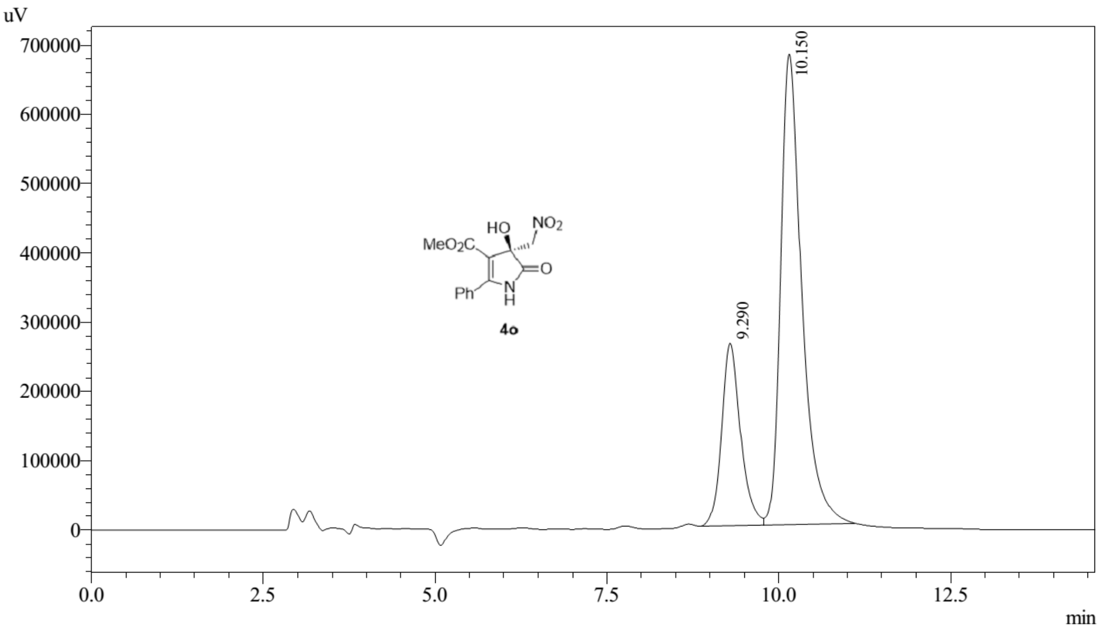

1 Det.A Ch1 / 254nm

Detector A Ch1 254nm

Peak#	Ret. Time	Area	Height	Area %	Height %
1	9.290	5072251	263148	25.759	27.946
2	10.150	14619258	678475	74.241	72.054
Total		19691508	941624	100.000	100.000

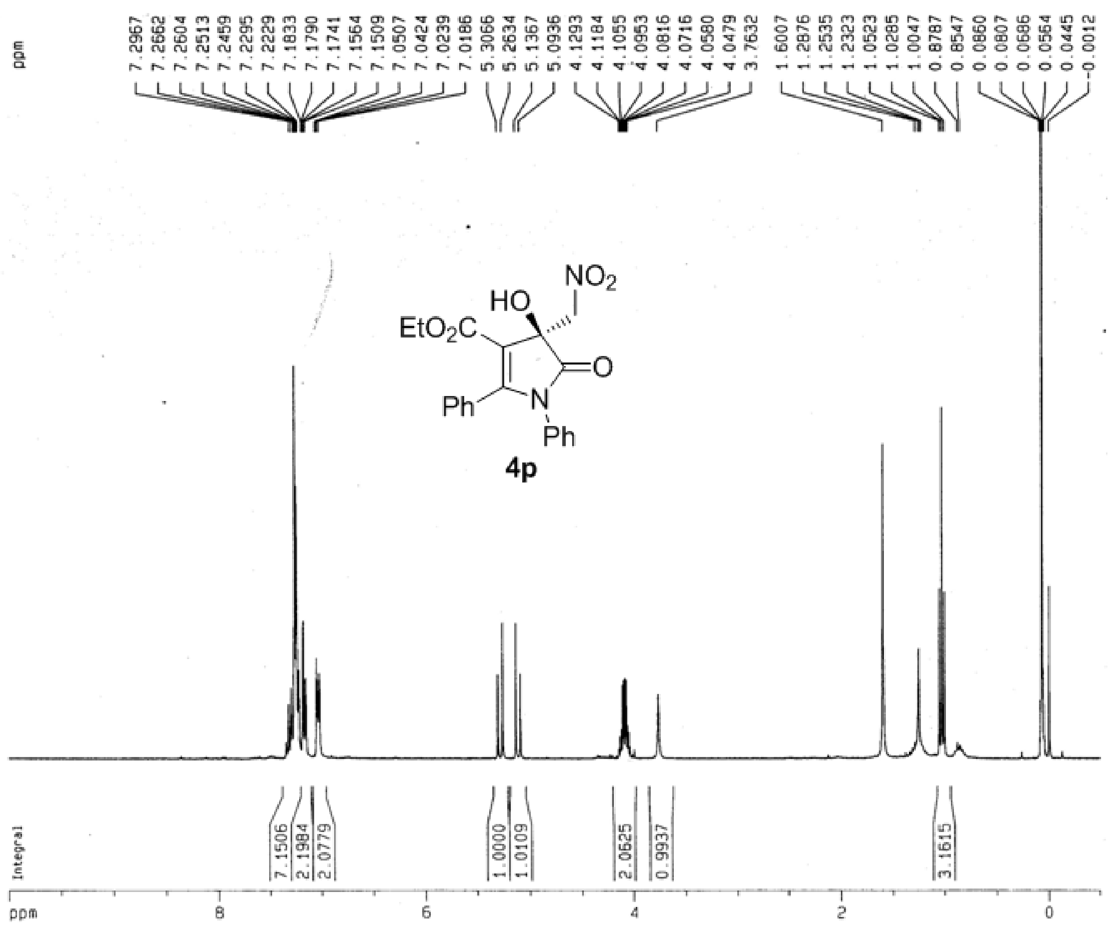
4p

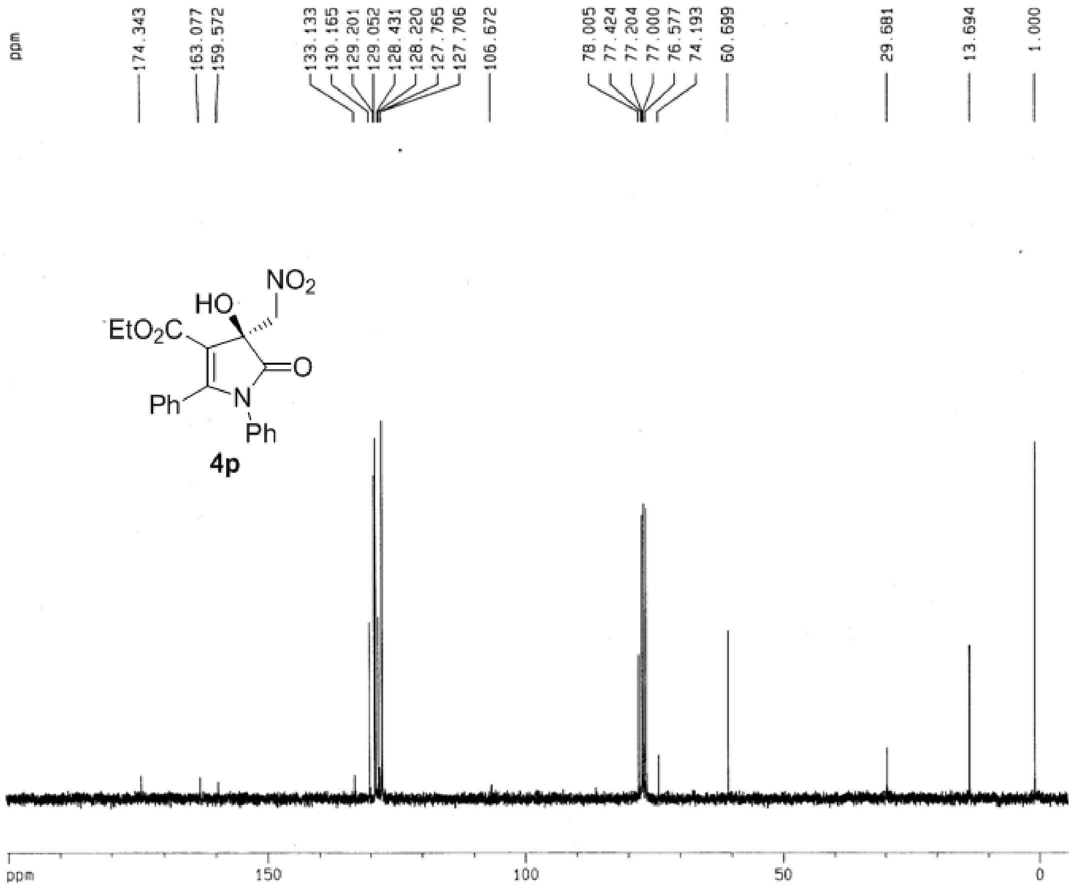
4p

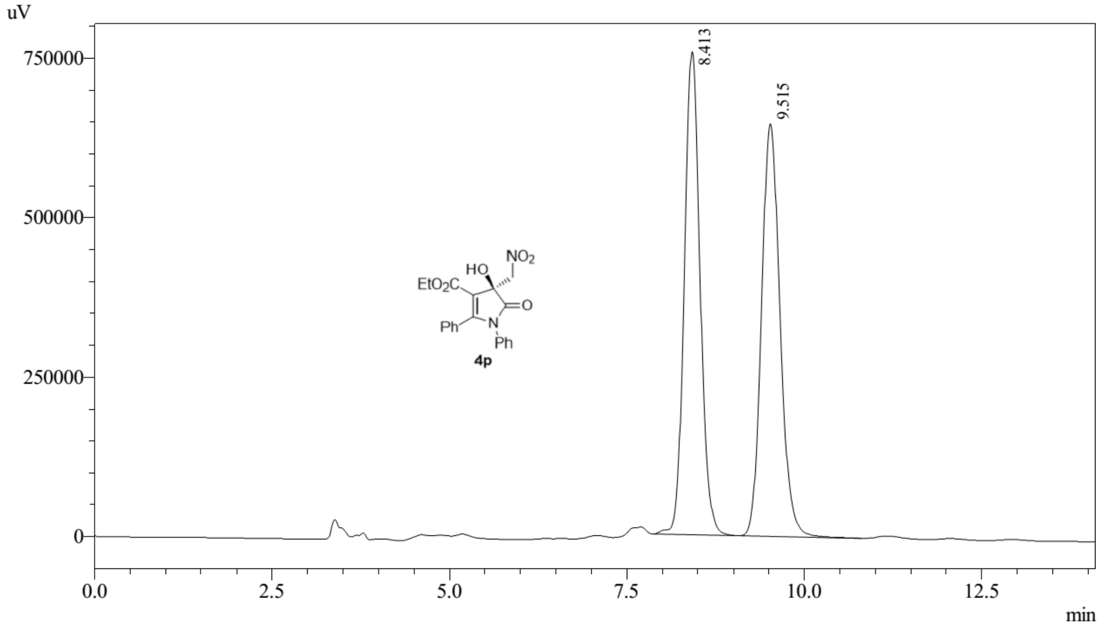

1 Det.A Ch1 / 254nm

Detector A Ch1 254nm

Peak#	Ret. Time	Area	Height	Area %	Height %
1	8.413	11754086	757832	49.938	53.951
2	9.515	11783436	646823	50.062	46.049
Total		23537523	1404654	100.000	100.000

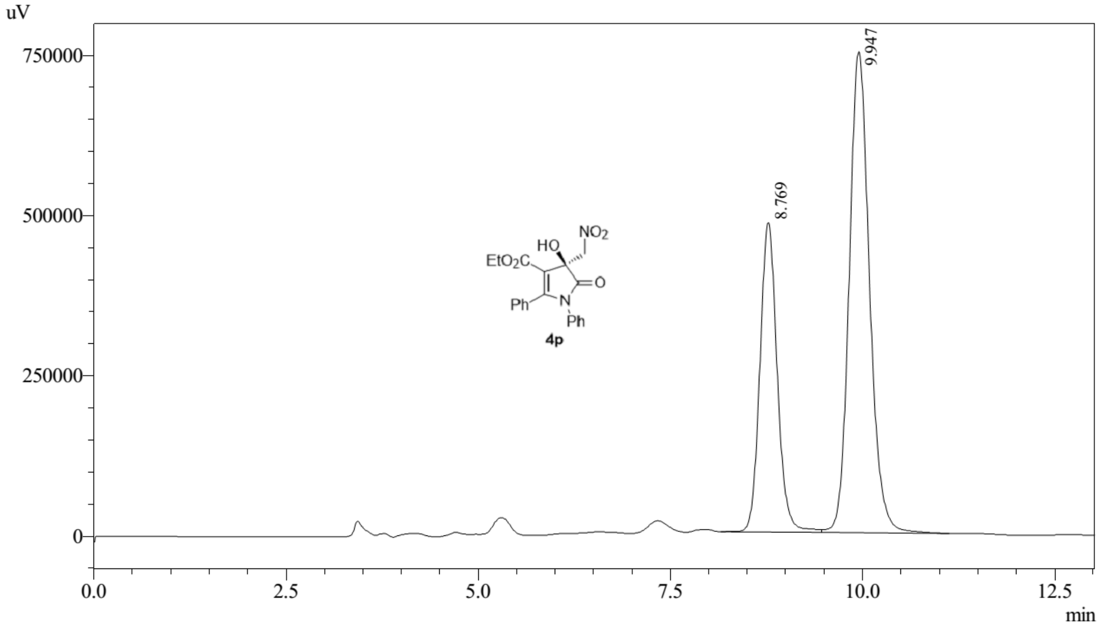

1 Det.A Ch1 / 254nm

Detector A Ch1 254nm

Peak#	Ret. Time	Area	Height	Area %	Height %
1	8.769	7540260	482303	35.341	39.120
2	9.947	13795492	750573	64.659	60.880
Total		21335753	1232876	100.000	100.000

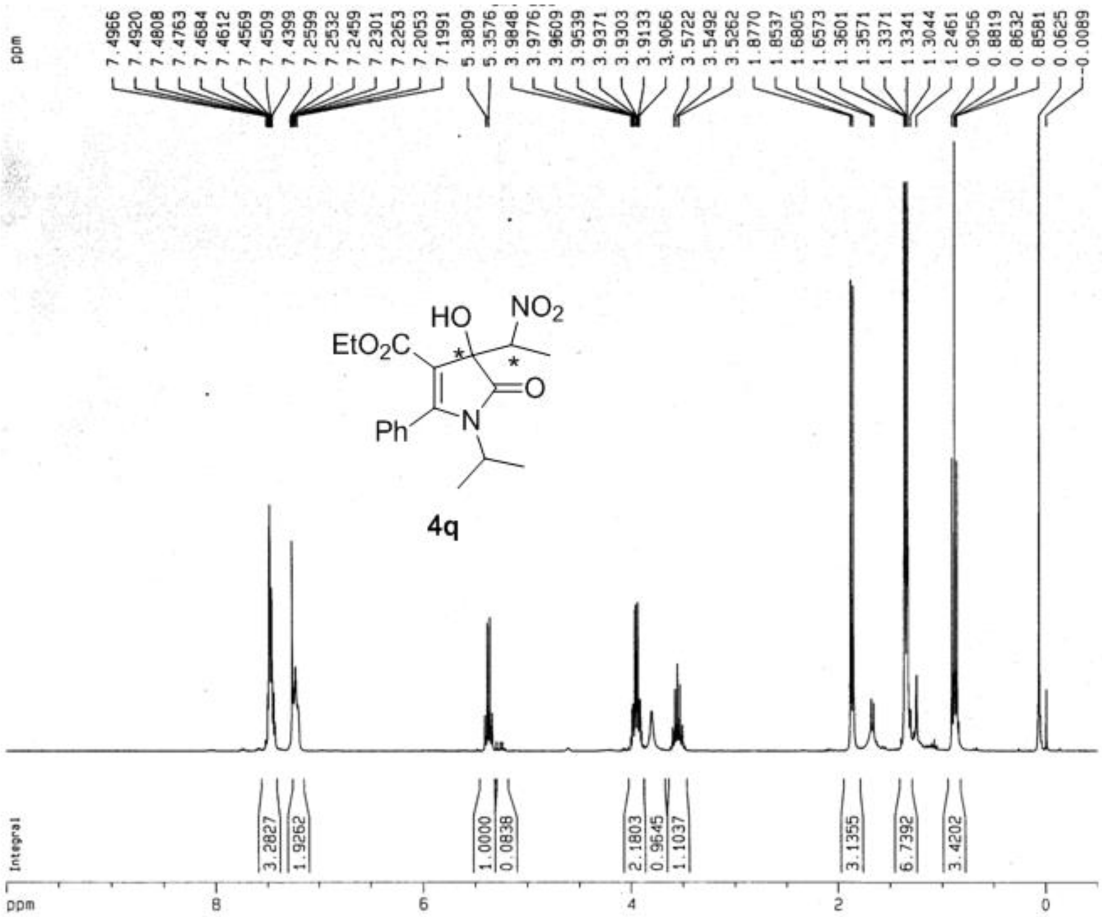
ppm
7.4966 7.4920 7.4808 7.4763 7.4684 7.4612 7.4569 7.4509 7.4399 7.2599 7.2532 7.2459 7.2301 7.2263 7.2053 7.1991 5.3809 5.3576 3.9848 3.9776 3.9609 3.9539 3.9371 3.9303 3.9133 3.9066 3.5722 3.5492 3.5262 1.8770 1.8537 1.6805 1.6573 1.3601 1.3571 1.3371 1.3341 1.3044 1.2461 0.9056 0.8819 0.8632 0.8581 0.0625 -0.0089
HO
NO2
EtO2C
Ph
N
O
4q
Integral
3.2827 1.9262 1.0000 0.0838 2.1803 0.9645 1.1037 3.1355 6.7392 3.4202
ppm 8 6 4 2 0

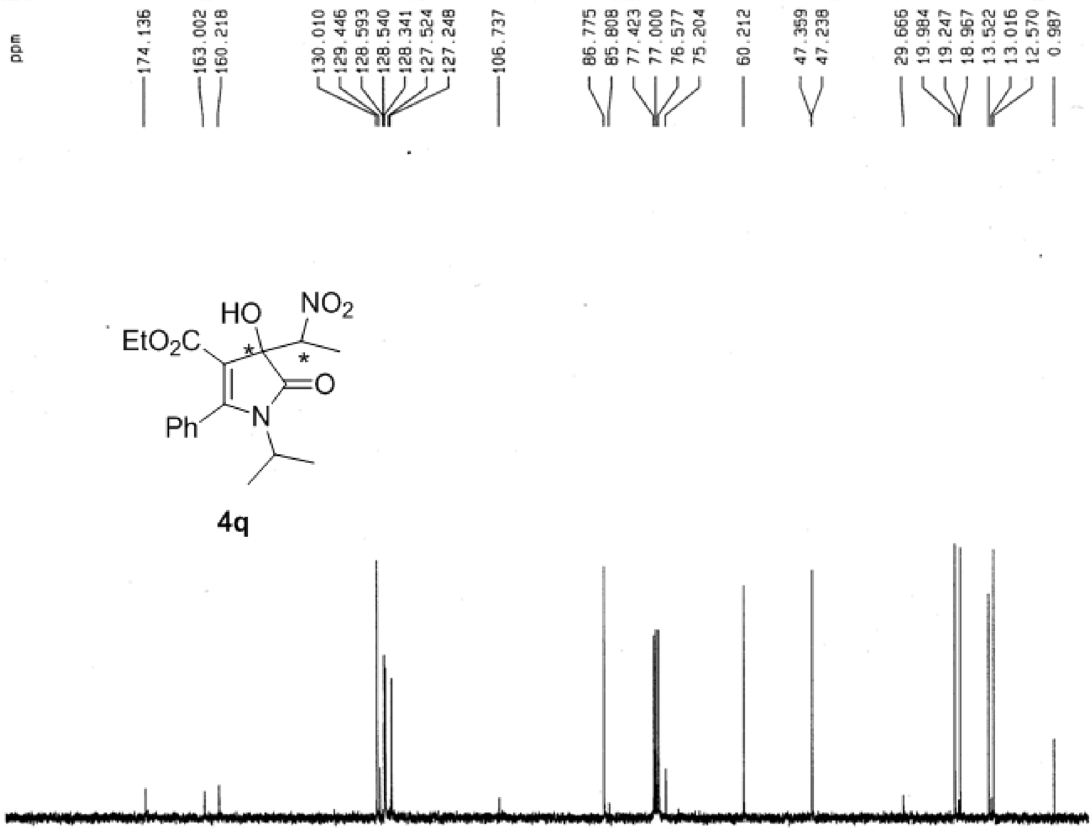
ppm
174.136 163.002 160.218 130.010 129.446 128.593 128.540 128.341 127.524 127.248 106.737 86.775 85.808 77.423 77.000 76.577 75.204 60.212 47.359 47.238 29.666 19.984 19.247 18.967 13.522 13.016 12.570 0.987
HO
NO2
EtO2C
Ph
N
O
4q
ppm 150 100 50 0

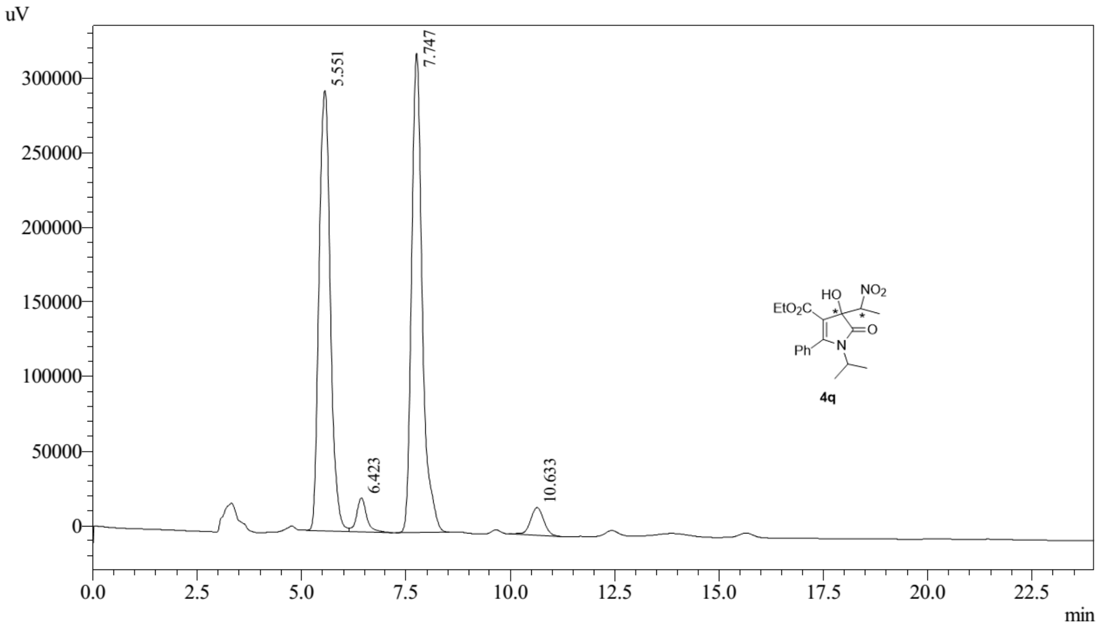

1 Det.A Ch1 / 254nm

Detector A Ch1 254nm

Peak#	Ret. Time	Area	Height	Area %	Height %
1	5.551	5557076	294892	46.076	44.861
2	6.423	373667	22666	3.098	3.448
3	7.747	5714429	321167	47.381	48.858
4	10.633	415383	18628	3.444	2.834
Total		12060555	657353	100.000	100.000

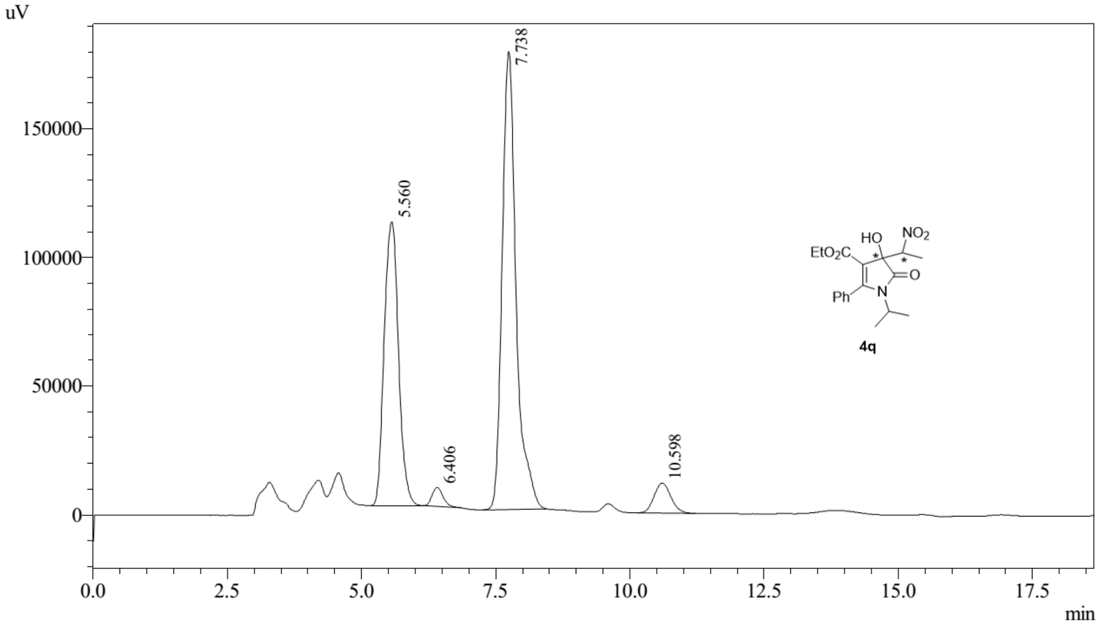

1 Det.A Ch1 / 254nm

Detector A Ch1 254nm

Peak#	Ret. Time	Area	Height	Area %	Height %
1	5.560	2044644	110373	36.488	35.905
2	6.406	105826	7392	1.889	2.405
3	7.738	3199376	177966	57.095	57.893
4	10.598	253724	11674	4.528	3.798
Total		5603570	307405	100.000	100.000

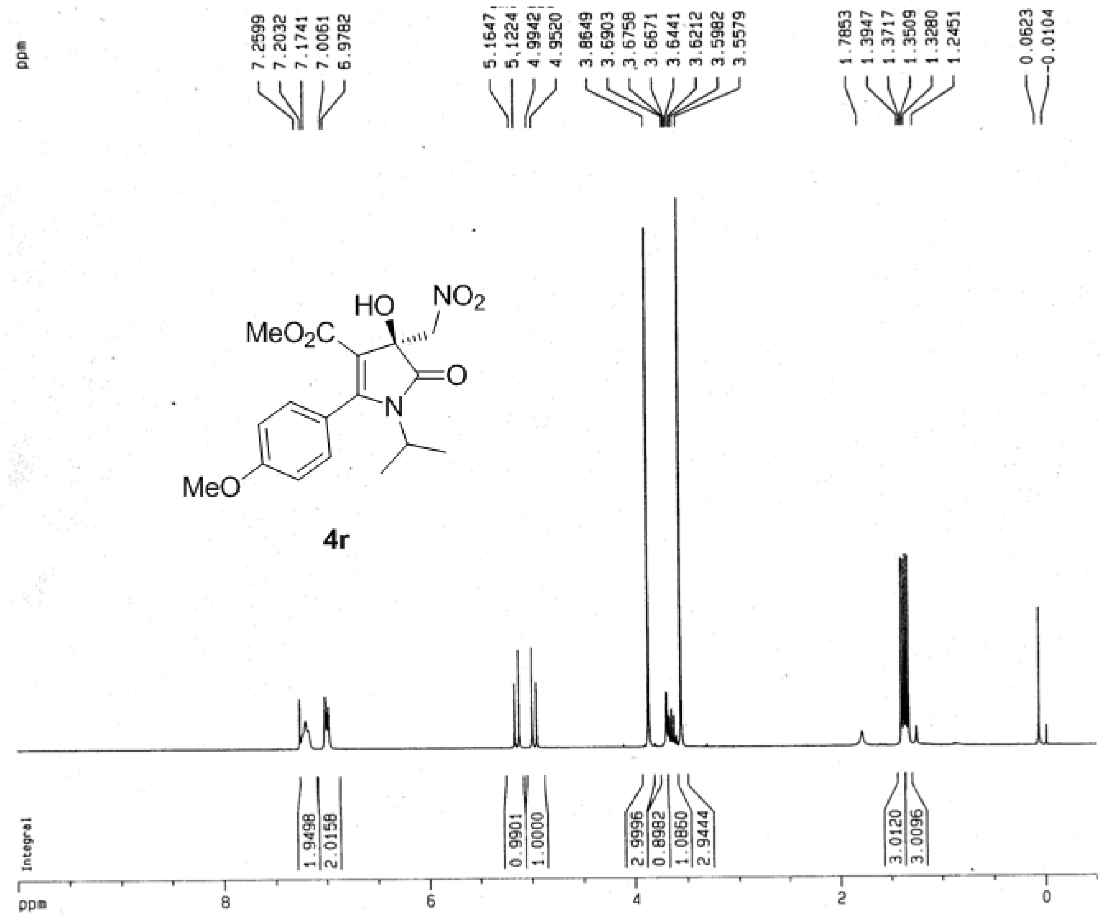
HO
NO2
MeO2C
O
N
MeO
4r
ppm
7.2599
7.2032
7.1741
7.0061
6.9782
5.1647
5.1224
4.9942
4.9520
3.8649
3.6903
3.6758
3.6671
3.6441
3.6212
3.5982
3.5579
1.7853
1.3947
1.3717
1.3509
1.3280
1.2451
0.0623
-0.0104
Integral
1.9498
2.0158
0.9901
1.0000
2.9996
0.8982
1.0860
2.9444
3.0120
3.0096
8
6
4
2
0

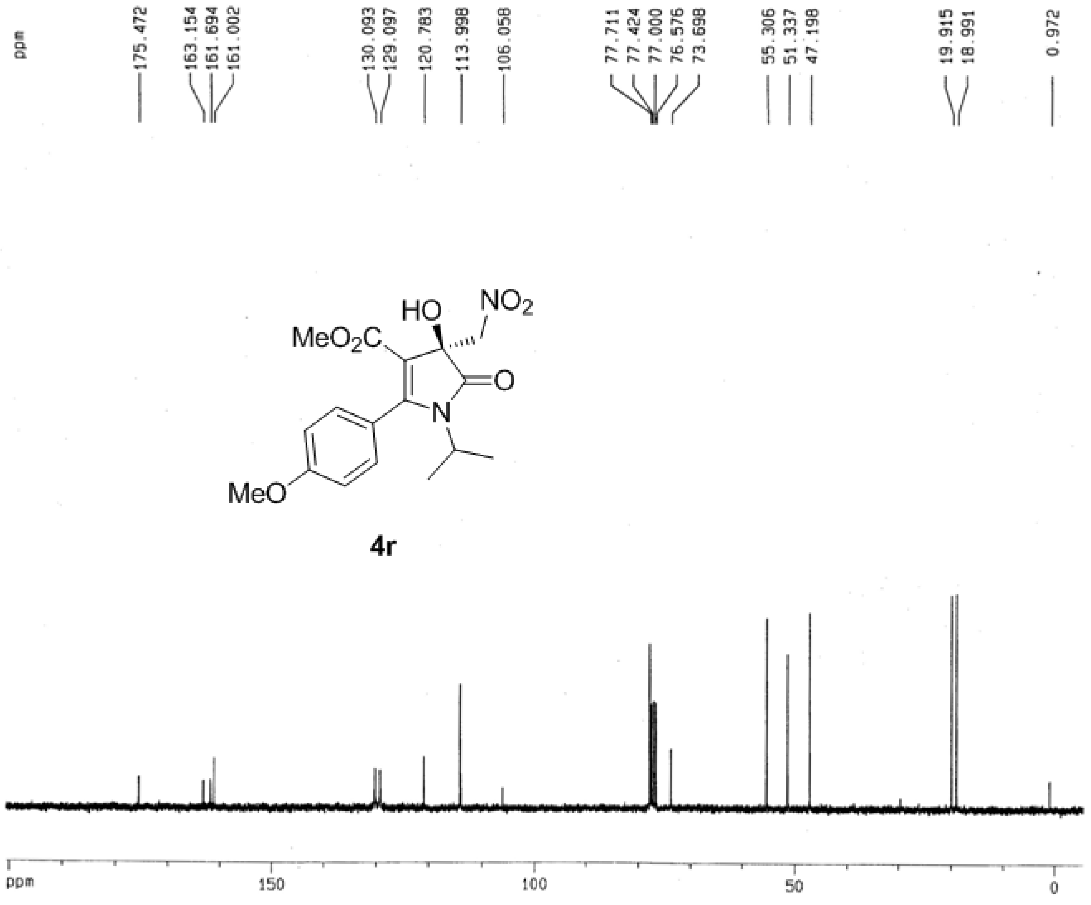
ppm
175.472
163.154
161.694
161.002
130.093
129.097
120.783
113.998
106.058
77.711
77.424
77.000
76.576
73.698
55.306
51.337
47.198
19.915
18.991
0.972
HO
NO2
MeO2C
O
N
MeO
4r
150
100
50
0

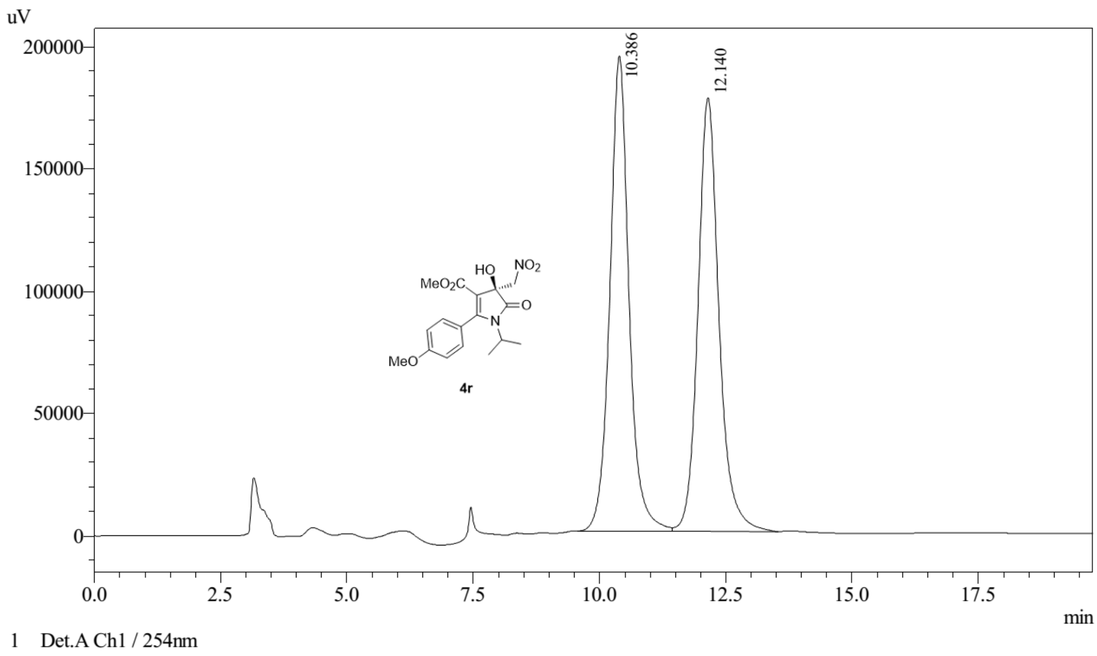

Detector A Ch1 254nm

Peak#	Ret. Time	Area	Height	Area %	Height %
1	10.386	5122338	194251	49.979	52.291
2	12.140	5126683	177233	50.021	47.709
Total		10249021	371485	100.000	100.000

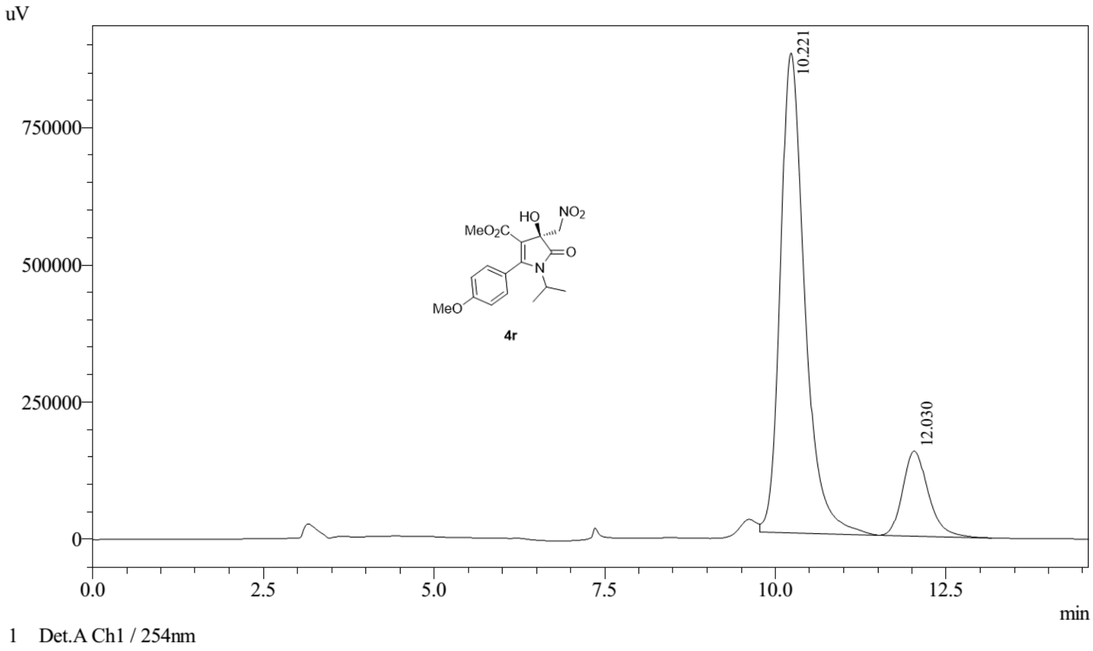

Detector A Ch1 254nm

Peak#	Ret. Time	Area	Height	Area %	Height %
1	10.221	21797879	873916	84.357	84.965
2	12.030	4042184	154645	15.643	15.035
Total		25840063	1028560	100.000	100.000

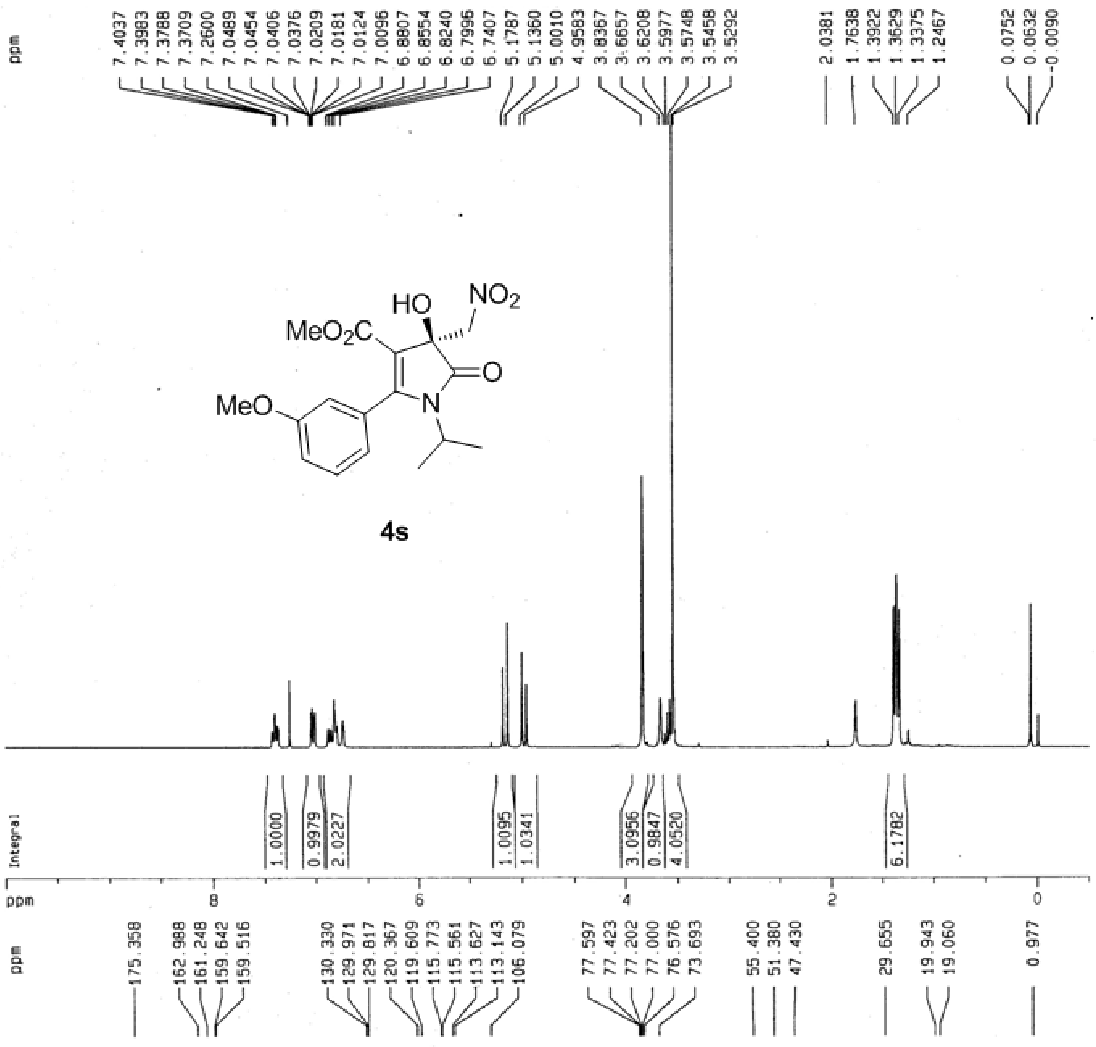
4s

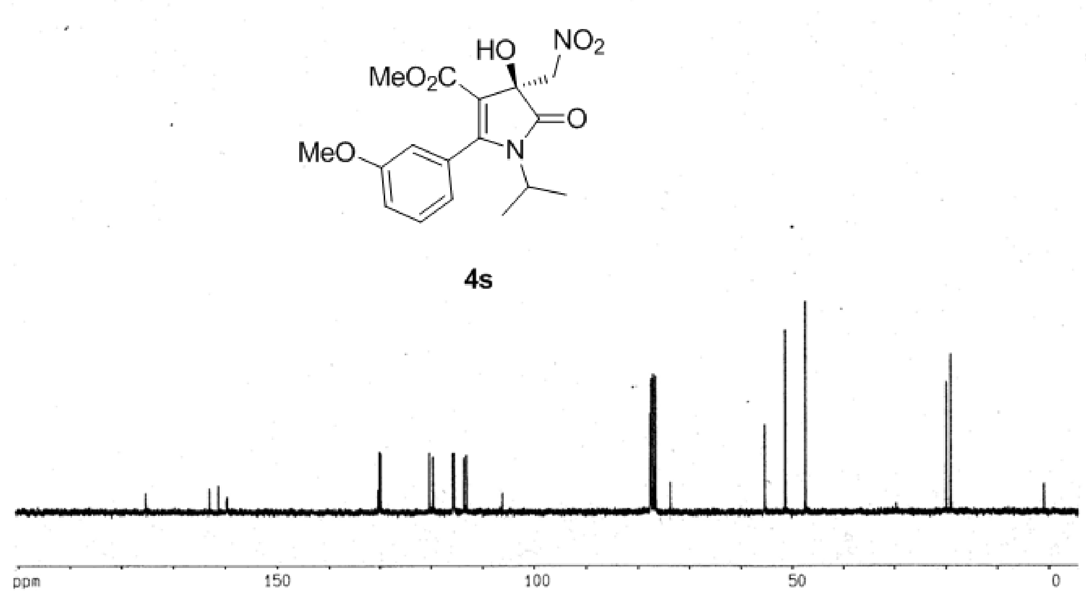
4s

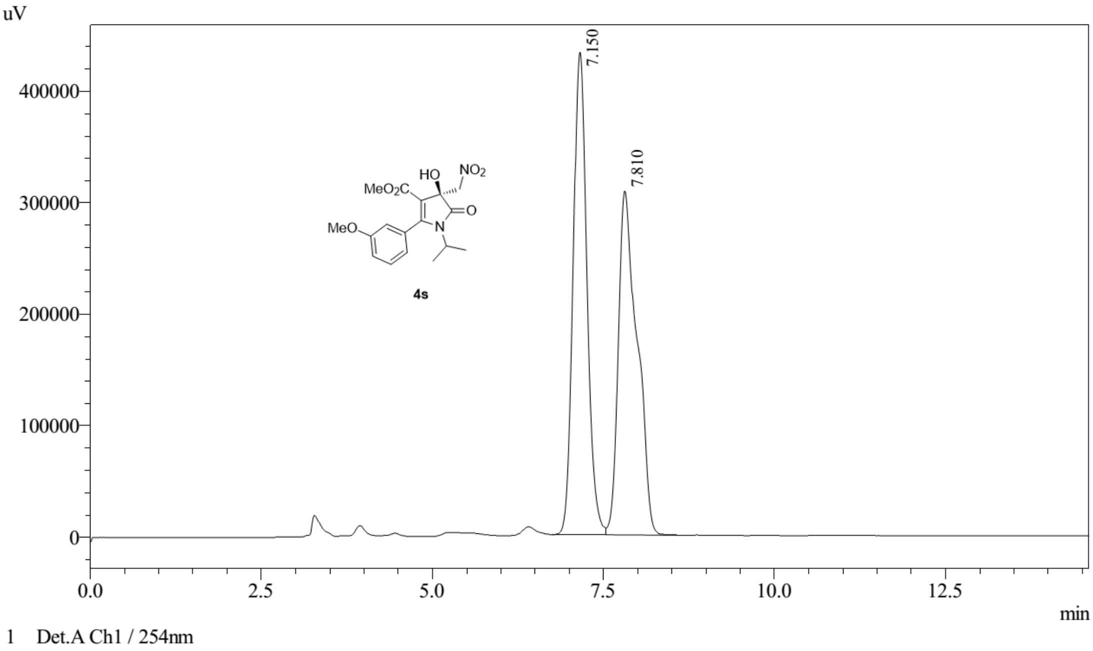

Detector A Ch1 254nm

Peak#	Ret. Time	Area	Height	Area %	Height %
1	7.150	6076688	432784	50.611	58.372
2	7.810	5929991	308639	49.389	41.628
Total		12006678	741422	100.000	100.000

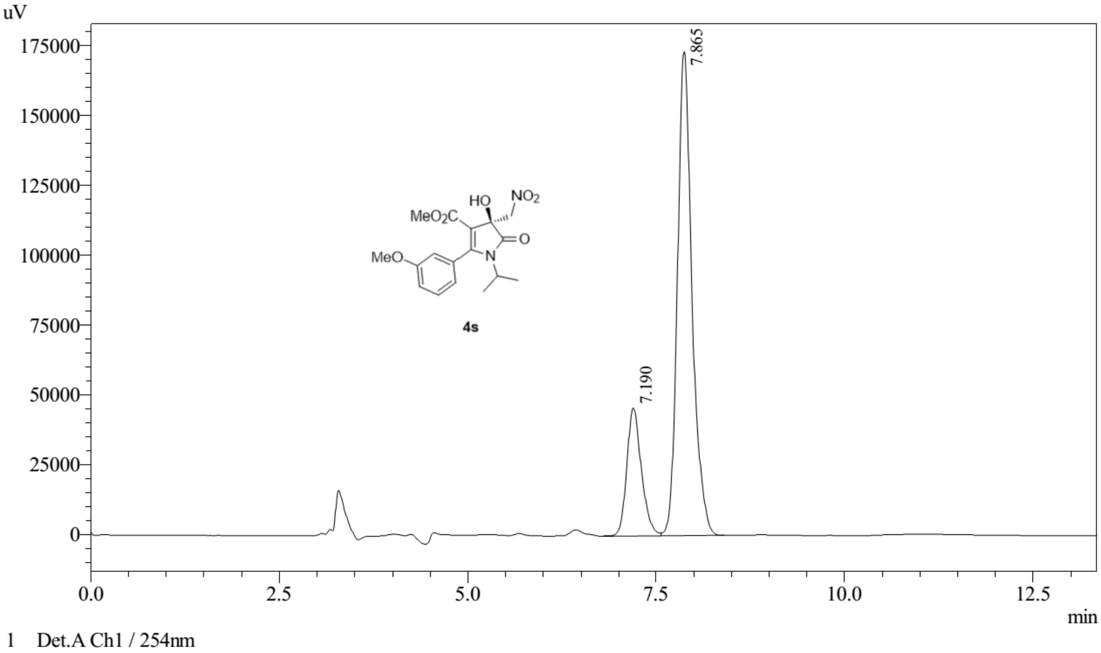

Detector A Ch1 254nm

Peak#	Ret. Time	Area	Height	Area %	Height %
1	7.190	621115	45812	21.023	20.918
2	7.865	2333303	173194	78.977	79.082
Total		2954418	219005	100.000	100.000
